# Synthetic Flavonoid Analogues as CatB/CatL Inhibitors with Potential Anti-SARS-CoV‑2 Activity

**DOI:** 10.1021/acsomega.6c07800

**Published:** 2026-10-06

**Authors:** Estela M. G. Lourenço, Marli de F. C. Emiliano, Silvana Ribeiro, Felipe A. Clarindo, Luan D. S. Vianna, Thaís de F. S. Moraes, Erik V. de S. Reis, Aline A. de Souza, Luiza V. Barreto, Mauricio F. M. Machado, Adilson Beatriz, Letícia G. de Pontes, Mariana O. de Godoy, Glaucius Oliva, Rafael V. C. Guido, Jordana G. A. Coelho-dos-Reis, Wagner A. de S. Judice, Dênis P. de Lima, Rafaela S. Ferreira

**Affiliations:** † Laboratório de Modelagem Molecular e Planejamento de Fármacos (LMMPF), Departamento de Bioquímica e Imunologia, Instituto de Ciências Biológicas, Universidade Federal de Minas Gerais, Av. Antônio Carlos, 6627, Pampulha, Belo Horizonte, Minas Gerais 31270-901, Brazil; ‡ Interdisciplinary Center for Biochemical Research (CIIB), 133647 University of Mogi das Cruzes (UMC), Av. Dr. Cândido Xavier de Almeida Souza, 200, Centro Cívico, Mogi das Cruzes, São Paulo 08780-911, Brazil; § ProsPeRa Research Group, Grupo de Prospecção e Pesquisas Em Resposta Imune e Antivirais, Departamento de Microbiologia, Instituto de Ciências Biológicas, Universidade Federal de Minas Gerais, Av. Antônio Carlos, 6627, Pampulha, Belo Horizonte, Minas Gerais 31270-901, Brazil; ∥ Laboratório de Síntese e Transformação de Moléculas Orgânicas (SINTMOL), 54534 Instituto de Química, Universidade Federal de Mato Grosso do Sul, Avenida Senador Filinto Müller, 1555 Cidade Universitária, Campo Grande, Mato Grosso do Sul 79074-460, Brazil; ⊥ Laboratório de Biodados, Departamento de Bioquímica e Imunologia, Instituto de Ciências Biológicas, Universidade Federal de Minas Gerais, Av. Antônio Carlos, 6627, Pampulha, Belo Horizonte, Minas Gerais 31270-901, Brazil; # Instituto de Física de São Carlos (IFSC), 117186 Universidade de São Paulo (USP), Av. Trabalhador São-Carlense, 400, São Carlos, São Paulo 13566-590, Brazil

## Abstract

The COVID-19 pandemic profoundly affected global public health. Intensive efforts have led to the development of vaccines and antiviral drugs. However, the emergence of SARS-CoV-2 variants has raised concerns about resistance to therapies, making the exploration of alternative molecular pathways a promising strategy. Cathepsins stand out in this context due to their crucial role in mediating SARS-CoV-2 entry via endosomes. Structurally, these enzymes share high similarity with proteases from other organisms such as *Leishmania* CPB. Previously, we reported the synthesis and activity of 17 flavonoid derivatives as CPB inhibitors with robust antimicrobial activity. Motivated by the high resemblance between the active sites of CPB and CatB, we evaluated the anti-SARS-CoV-2 activity of these compounds and validated cathepsins as their main molecular target. Among them, nine flavonoid derivatives exhibited potent simple-linear noncompetitive dual CatB/CatL inhibition, with K_*i*_ values ranging from 3.20 to 29.05 μM for CatB and from 2.41 to 12.44 μM for CatL. Chalcones **1c**, **2a**, and **2b** translated this enzymatic activity into significant reductions in SARS-CoV-2 titers (IC_50_ = 12.06, 1.05, and 2.12 μM, respectively), while demonstrating low toxicity in both Vero-CCL81 and L929 cells. Their potential direct effects on viral replication were also evaluated using SARS-CoV-2 main protease assays and virucidal tests. No compound showed activity on viral targets, suggesting that cathepsin inhibition is their potential antiviral mechanism. Although additional mechanistic assays are still required, these data provide initial support for the involvement of dual CatB/CatL as antiviral targets and offer insights for the development of chalcone-based leads against COVID-19.

## Graphical abstract

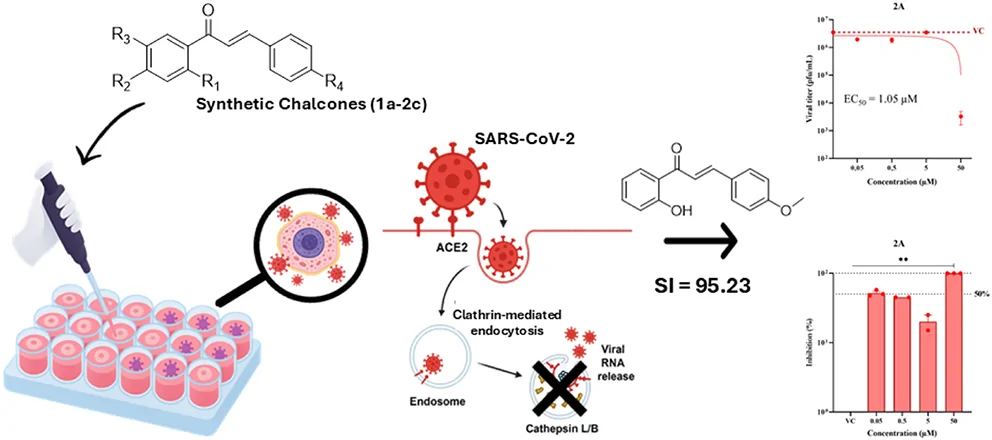

## 1 Introduction

In early 2020, the World Health Organization (WHO) declared COVID-19 a pandemic, marking the beginning of a pivotal period in public health history.^1^ By that time, the disease caused by the highly infectious β-coronavirus, SARS-CoV-2, had already resulted in 2,977 deaths.^2^ An intense race among scientists to enhance understanding of disease outcomes and develop new treatments quickly began. Scientific efforts and public financial investments have led to significant progress: over three years, approved vaccines and antiviral therapies have been developed and successfully applied in the prevention and treatment of COVID-19.^3,4^ In May 2023, following 6.9 million deaths since the onset of the pandemic, the WHO declared that COVID-19 was no longer classified as a public health emergency.^5^ Despite significant advances in controlling the spread of the disease, the risk of SARS-CoV-2 infection and severe outcomes is a persistent threat.^6^ To date, over 7 million deaths have been officially reported, although the actual numbers may be 2 to 4 times higher due to underreporting,^7,8^ highlighting the urgent need for further studies to develop novel, safe, and effective treatments.

However, the discovery and development of novel antiviral agents for the treatment of COVID-19 remains an ongoing challenge. SARS-CoV-2, an RNA virus, is marked by discontinuous transcription that promotes frequent recombination and mutations, leading to the emergence of variants such as Alpha, Beta, Gamma, Delta, Lambda, and Omicron.^9^ These mutant strains have been linked to an increase in COVID-19 cases, raising significant concerns about the potential for resistance to current antiviral therapies.^7–10^ Indeed, strains isolated from immunocompromised patients were found to carry resistance mutations to the SARS-CoV-2 Main Protease (M^pro^) inhibitors nirmatrelvir and ensiltrevir, and RNA-dependent RNA polymerase (RdRp) inhibitor remdesivir.^11,12^ As a result, the effectiveness of approved anti-COVID-19 drugs is reduced, and these patients often require prolonged or repeated treatment courses.^13^ Given these limitations, exploring alternative molecular pathways may offer a strategy to overcome the rapid emergence of resistance to drugs targeting viral proteins.

Cathepsins have emerged as promising targets for drug development in this context. It is well established that these proteases, particularly cathepsin L (CatL), play a crucial role in SARS-CoV-2 entry into host cells via endosomes.^14^ Gallinamide A and K777, both potent inhibitors of CatL, effectively reduced SARS-CoV-2 infections in various cell lines. Neither of these compounds exhibited activity against M^pro^, suggesting that their antiviral effects are directly linked to the inhibition of cathepsin proteases.^15,16^ Another inhibitor of several cysteine proteases, including cathepsins, fluoromethylketone Z-FA-FMK demonstrated excellent anti-SARS-CoV-2 activity both *in vitro* and *in vivo*.^17^ Similarly, targeting CatL with the synthetic compound Calpeptin effectively reduced *in vivo* viral infection in SARS-CoV-2-infected hamsters.^14–18^ While CatL is the primary protease mediating SARS-CoV-2 Spike protein cleavage within endosomes, other cathepsins also play important roles in viral infection. Cathepsin S (CatS) is involved in inflammatory and immune-regulatory processes that are amplified during viral infection and has been associated with acute lung injury, underscoring its relevance in respiratory disease pathology.^18,19^ Cathepsin B (CatB) may also contribute to Spike protein activation, particularly under hyperinflammatory conditions associated with poor clinical outcomes.^20^ Consequently, a substantial number of anti-SARS-CoV-2 compounds have been reported to exhibit inhibitory activity against more than one cathepsin, including CatL, CatB, and CatS.^18–21^

Cysteine cathepsins were first characterized after the discovery of the lysosome in 1955.^22^ Considered the most abundant proteases in endo/lysosomal compartments, cathepsins are involved in several cellular processes, including intracellular protein degradation, energy metabolism, and immune responses.^23^ The discovery that cathepsins are secreted and retain functional activity outside the lysosome marked a paradigm shift, increasing their appeal as molecular targets for drug development in recent decades.^24^ They are also involved in cell death and inflammation signaling in the cytoplasm, cell cycle regulation in the nucleus, and play degradative and signaling roles in the extracellular environment when secreted by cells.^23^ Structurally, cathepsins contain a catalytic triad in their active site, featuring a cysteine residue that can act as a nucleophile.^25,26^ Based on their structure and enzymatic properties, cathepsins belong to the C1 family of papain-like enzymes, the largest and best-characterized family of cysteine peptidases.^22^ The structural and functional properties of cathepsins are similar to cysteine proteases from other organisms, including parasites, helminths, plants, and viruses. Importantly, these conserved structural and functional features also extend to their interactions with glycosaminoglycans (GAGs), such as heparin and heparan sulfate, suggesting a conserved regulatory mechanism across this family of proteases. Seminal studies on papain, the archetype of papain-like proteases, demonstrated that GAG binding induces significant conformational changes, alters substrate affinity, and protects the enzyme from alkaline pH-induced inactivation.^27^ This GAG-mediated allosteric modulation is a hallmark of the family and is also observed in human cathepsins, where GAG interactions contribute to anchoring the enzymes to the extracellular matrix and cell surfaces,^28^ while also modulating their stability and activity during tissue remodeling and hyperinflammatory responses.^29,30^ Collectively, these conserved structural and functional features have contributed to the classification of related proteases as cathepsin-like enzymes.^31,32^

*Leishmania* cysteine proteases (CPs) are examples of proteins that closely resemble human cathepsins. Among them, cysteine protease B (CPB) is extensively studied due to its high expression in the amastigote form and its importance in parasite survival within mammalian host cells.^33,34^ Several synthetic compounds, as well as natural products, have been shown to inhibit CPB and display significant activity against different isoforms of this enzyme.^35,36,37,38^ In a recent study conducted by our research group, the potential of natural products targeting CPB was confirmed, with 17 synthetic flavonol and chalcone analogues identified as potent inhibitors of this *Leishmania* protease.^39^ CPB is a cathepsin B-like enzyme, as its N-terminal amino acid sequence closely resemble and exhibit high homology with the mammalian cysteine protease, CatB.^33^ The active site is also strongly conserved between these enzymes, sharing up to 80% sequence identity.^40^ These structural similarities suggest that CatB may be a potential target for *Leishmania* CPB inhibitors, a hypothesis reinforced by several studies demonstrating that both natural products and synthetic compounds identified as human CatB inhibitors also exhibit effective inhibition against CPB.^36,41,42^ In addition, the scalable, easy-to-replicate, and cost-effective method used to synthesize flavonol and chalcone derivatives makes them affordable compounds.^34,39^ This is a crucial characteristic for molecules intended to treat highly infectious diseases, such as COVID-19, as they can be incorporated into a mass treatment strategy.

In the present study, we describe the investigation of the antiviral effects associated with the inhibition of cathepsin proteins by a series of synthetic chalcone and flavonol analogues. Enzymatic assays with cathepsins B and L were performed, followed by analysis of the inhibition mechanism of the most promising inhibitors. Antiviral activity was evaluated in SARS-CoV-2 infected cells, and additional potential mechanisms were investigated through enzymatic assays on viral targets and virucidal tests. The toxicity of the tested compounds against Vero and L-929 cells was also assessed to determine the selectivity index (SI).

## 2 Results and Discussion

### 2.1 Flavonoid Analogues Are Potent Simple Linear Noncompetitive Inhibitors of Cathepsins B and L

Among the 17 synthesized flavonoid derivatives previously characterized as *Leishmania* CPB inhibitors, nine compounds exhibited significant inhibitory activity against CatB. Compounds **2a** and **2b** stood out among chalcone analogues due to their low half-maximal inhibitory concentration (IC_50_ = 1.33 ± 0.06 and 0.95 ± 0.08 μM, respectively). Meanwhile, **f12a** and **f12b** (IC_50_ = 1.30 ± 0.05 and 0.78 ± 0.01 μM, respectively) were the most active flavonol derivatives (Table 1). Compared to synthetic flavonoids reported in the literature, these results represent a relevant advance in exploring these compounds as potential CatB inhibitors. In a previously reported series of 15 synthetic chalcones, the most potent analogue displayed 63.6 ± 4.7% inhibition at 125 μM, with no IC_50_ value determined.^43^ A subsequent study by Jeon et al. (2016) described two competitive chalcone-based inhibitors with IC_50_ values of 6.34 ± 1.60 and 11.05 ± 0.85 μM.^44^ Another study reporting a bioactivity-guided fractionation identified the flavonols myricetin and quercetin as the most active secondary metabolites from Cerrado plant extracts, showing promising CatB inhibition (IC_50_ = 4.9 and 8.2 μM, respectively).^41^ Notably, all three flavonol analogues tested in the present work exhibited lower IC_50_ values, highlighting the relevance of these results.

**Table 1:** Half-Maximal Inhibitory Concentration (IC_50_) Values of Synthetic Flavonol and Chalcone Analogues against Cathepsins B and L in the Presence or Absence of Heparin

	IC_50_ (μM)^a^			
Compounds	CatB	CatB + heparin^b^	CatL	CatL + heparin^b^
1a	4.31 ± 0.23	3.13 ± 0.16	1.01 ± 0.05	5.62 ± 0.18
1b	1.56 ± 0.04	3.37 ± 0.15	4.96 ± 0.45	0.71 ± 0.07
1c	4.69 ± 0.20	6.87 ± 0.40	1.52 ± 0.10	0.54 ± 0.02
2a	1.33 ± 0.06	3.65 ± 0.11	5.22 ± 0.33	2.92 ± 0.29
2b	0.95 ± 0.08	2.08 ± 0.04	2.61 ± 0.22	2.64 ± 0.10
2c	1.75 ± 0.04	2.51 ± 0.09	1.20 ± 0.09	4.19 ± 0.19
F12a	1.30 ± 0.05	1.79 ± 0.05	1.12 ± 0.06	1.00 ± 0.05
F12b	0.78 ± 0.01	1.88 ± 0.06	1.29 ± 0.07	0.77 ± 0.25
F12c	1.70 ± 0.02	2.32 ± 0.10	4.60 ± 0.25	1.32 ± 0.03

a Data are presented as the mean ± standard error of the mean (SEM) from two independent experiments, each performed in triplicate.

b Assays were performed in the presence of 10 μM heparin.

CatB and CatL possess highly similar active sites, differing primarily by the presence of the occluding loop in CatB and by amino acid residues in the S2 subsite, which alters its substrate specificity. The absence of this loop in CatL enables the accommodation of larger hydrophobic side chains within its S1 pocket, as shown by structural studies.^45,46^ Due to the strong resemblance between their active sites, we also evaluated the nine CatB inhibitors against CatL, and all compounds exhibited promising inhibitory activity against this enzyme (Table 1). Particularly, compounds **1a** and **f12a** (IC_50_ = 1.01 ± 0.05 and 1.12 ± 0.06 μM, respectively) stood out due to their high inhibitory potency. Consistent with the CatB inhibition results, the data on CatL inhibition by our synthetic flavonoids are noteworthy compared with reported IC_50_ values for different flavonoid subclasses. A recent study, using machine learning methods, has identified three chalcones and one flavanone as CatL inhibitors, with IC_50_ values ranging from 36.3 to 100 μM, which are considerably higher than those of the compounds evaluated in our work.^47^

Additionally, an important aspect in the development of dual CatB/CatL inhibitors is the potential impact of heparin on their inhibitory potency. Widely known as an anticoagulant, heparin is a highly sulfated glycosaminoglycan (GAG) capable of interacting with various proteins, including CatB and CatL.^29,48,49^ Data accumulated over the past decades shows that, beyond modulating enzymatic activity, heparin may also influence inhibitor binding, protein stability, and cellular uptake.^50,51,52^ Concentrations in the micromolar range, such as 10 μM, are commonly used in *in vitro* studies of heparin–protein interactions, as heparin can serve as an in vitro surrogate for heparan sulfate (HS) and other glycosaminoglycans (GAGs) that interact with cysteine cathepsins in cellular and extracellular environments.^27^ In the context of SARS-CoV-2 infection, CatB and CatL contribute to Spike protein processing within endosomal/lysosomal compartments, where interactions with GAGs may influence protease conformation and activity.^53^ The selected concentration of 10 μM is within the range of the apparent dissociation constant reported for the heparin–CatB interaction (*K*_d_ ≈ 18 μM),^29^ supporting its relevance for studying enzyme–GAG interactions. Moreover, under inflammatory or pathological conditions, GAG concentration is elevated, enhancing its effects on proteases and their inhibitors. During SARS-CoV-2 infection, immune cells, such as mast cells, become activated, leading to the release of inflammatory mediators, including heparin.^54^ Consequently, compounds that maintain or increase inhibitory activity against CatB and CatL in the presence of high levels of heparin are desirable for the treatment of inflammatory diseases, including COVID-19.

The modulation of the inhibitory activity of the tested compounds against CatB and CatL by heparin was found to be dependent on the enzyme and its structural architecture (Table 1). Heparin effect on CatB inhibition was more pronounced, as all synthetic compounds, except chalcone **1a**, showed a decrease in potency, with up to a 5-fold increase in IC_50_. Compounds **2b** and **f12b**, the most potent CatB inhibitors, exhibited a 2-fold increase in their IC_50_ and only compound **1a** showed a modest gain in potency (4.31 → 3.13 μM). These results are consistent with the well-characterized conformational dynamics of CatB, whose occluding loop binds strongly to heparin, heparan sulfate, and other polyanionic ligands.^55^ Such interactions induce a conformational stabilization that reduces loop flexibility and protects active-site integrity under destabilizing conditions.^30^ By favoring these less accessible conformations, heparin hinders the optimal positioning of several synthetic inhibitors, thereby reducing their inhibitory potency, as observed for the flavonoid-based compounds described in this work.^52^

In contrast, the presence of heparin produced distinct effects on CatL inhibition. The polysaccharide enhanced the inhibitory potency of compounds such as **1b** (IC_50_: 4.96 → 0.71 μM) and **f12c** (4.60 → 1.32 μM), while attenuating the activity of others, including chalcones **1a** and **2c** (1.01 → 5.62 μM and 1.20 → 4.19 μM, respectively). Previous studies have demonstrated that CatL is intrinsically more sensitive to polyanionic modulators than CatB.^55^ Heparin has been shown to promote the formation of stable ternary enzyme–heparin–inhibitor complexes, thereby increasing inhibitory potency, as described for endogenous serpins.^56^ In combination with the absence of an occluding loop in CatL, which results in a more open, accessible, and permissive active site, these features allow the polysaccharide to enhance potency for structurally compatible compounds.^55,56^ Conversely, inhibitors whose binding depends on specific electrostatic interactions may experience competitive interference in the presence of heparin, leading to reduced potency. This behavior has been previously reported for synthetic ligands lacking geometric compatibility with the heparin-stabilized conformation of the CatL active site.^55^ Accordingly, depending on the chemical nature of the inhibitor, heparin may act either as a facilitator or as a competitor of CatL binding.^55,57^

The dual inhibition observed by our synthesized compounds raises an important discussion regarding selectivity. For many therapeutic purposes, higher selectivity is desirable to minimize adverse effects. On the other hand, compounds with dual or multiple inhibitory properties, known as multitarget drugs, have gained increasing attention in recent years due to their ability to treat complex diseases.^58^ For COVID-19 treatment, simultaneous inhibition of CatB and CatL may lead to synergistic or complementary pharmacological effects and have been shown to be important for the development of effective prototypes.^21,59,60^ In a previous study, a nitroxoline derivative that inhibits both CatB and CatL exhibited a low EC_50_ value (4.5 ± 2.1 μM) in dose–response assays, whereas GCV-5, a specific CatL inhibitor, showed no antiviral activity against SARS-CoV-2 infected Vero cells,^60^ highlighting the relevance of dual cathepsin inhibition. Given their high inhibitory potency against CatB and CatL, all nine flavonoid derivatives evaluated in this study are considered promising scaffolds for COVID-19 drug development.

Similar to its effects on IC_50_ values, heparin can modulate compound potency by altering their apparent K_*i*_. Investigating CatL inhibition by SCCA-1, Higgins et al. (2009) confirmed the direct influence of heparin on the inhibition mechanism, demonstrating that it promotes ternary complex formation and increases the second-order rate constant by more than 180-fold.^56^ Based on this evidence, we conducted a mechanistic analysis of all nine flavonol and chalcone derivatives, in the presence or absence of heparin, to elucidate their inhibitory mechanisms and assess how the GAGA-supplemented condition influences their K_*i*_ values.

The inhibition constants for the free enzyme (K_*i*_), calculated for CatB and CatL using the Michaelis–Menten model, confirmed the high inhibitory potency of the nine evaluated synthetic compounds (Table 2). For all chalcone and flavonol derivatives, the Lineweaver–Burk plots showed intersecting lines on the *x*-axis (Supporting Information, Figures S1–S18), and the K_*i*_/αK_*i*_ ratios were approximately equal to 1 (Table 2). These kinetic observations indicate a noncompetitive inhibition mechanism, which was unaffected by 10 μM heparin, with only minimal changes (always lower than 2-fold) in K_*i*_ for some compounds.

**Table 2:** Inhibition Constants (K_i_ and αKi) of Cathepsins B and L by Flavonoid Derivatives in the Absence and Presence of 10 μM Heparin

	CATHEPSIN B^a^			
	Without heparin		Heparin 10 μM	
Compounds	K_*i*_	αK_*i*_	K_*i*_	αK_*i*_
1a	10.11 ± 0.88	10.07 ± 0.39	9.13 ± 0.16	9.14 ± 0.17
1b	7.16 ± 0.24	7.83 ± 0.26	10.46 ± 0.17	10.62 ± 0.47
1c	6.39 ± 0.21	6.87 ± 0.36	8.83 ± 0.26	8.58 ± 0.16
2a	3.20 ± 0.07	3.29 ± 0.11	2.12 ± 0.01	2.15 ± 0.05
2b	22.08 ± 0.71	22.69 ± 0.32	34.31 ± 1.17	33.97 ± 0.93
2c	29.05 ± 0.20	29.36 ± 1.98	18.42 ± 0.64	18.41 ± 0.69
f12a	27.52 ± 1.38	26.93 ± 0.76	ND^b^	ND^b^
f12b	19.35 ± 0.64	19.62 ± 0.49	ND^b^	ND^b^
f12c	26.42 ± 1.12	25.96 ± 1.12	23.11 ± 0.55	22.75 ± 0.40

	CATHEPSIN L^a^			
	Without heparin		Heparin 10 μM	
Compounds	K_*i*_	αK_*i*_	K_*i*_	αK_*i*_
1a	2.41 ± 0.08	2.44 ± 0.11	3.08 ± 0.16	3.05 ± 0.10
1b	11.29 ± 0.41	11.56 ± 0.30	15.17 ± 0.72	14.84 ± 0.67
1c	3.35 ± 0.22	3.46 ± 0.11	4.60 ± 0.25	4.65 ± 0.29
2a	12.44 ± 0.35	13.18 ± 0.45	18.63 ± 0.45	18.30 ± 0.82
2b	6.34 ± 0.14	6.06 ± 0.15	3.49 ± 0.18	3.48 ± 0.12
2c	3.11 ± 0.03	3.16 ± 0.15	3.68 ± 0.10	3.28 ± 0.12
f12a	2.69 ± 0.13	2.81 ± 0.11	3.34 ± 0.05	3.40 ± 0.09
f12b	3.31 ± 0.21	2.66 ± 0.06	2.69 ± 0.13	2.66 ± 0.06
f12c	10.36 ± 0.29	10.74 ± 0.62	12.11 ± 0.25	12.00 ± 0.04

a Data are presented as mean ± standard error of the mean (SEM) in micromolar, obtained from experiments performed in duplicates.

b ND: not determined.

### 2.2 Electron-Donating and Polar Substituents Are Crucial for Cathepsin Inhibition by Flavonoid Derivatives

In our study, we investigated flavonol and chalcone analogues bearing substituents with diverse chemical properties at multiple positions on rings A and B to assess their potential impact on the structure–activity relationship against CatB and CatL (Figure 1).

**Figure 1:**
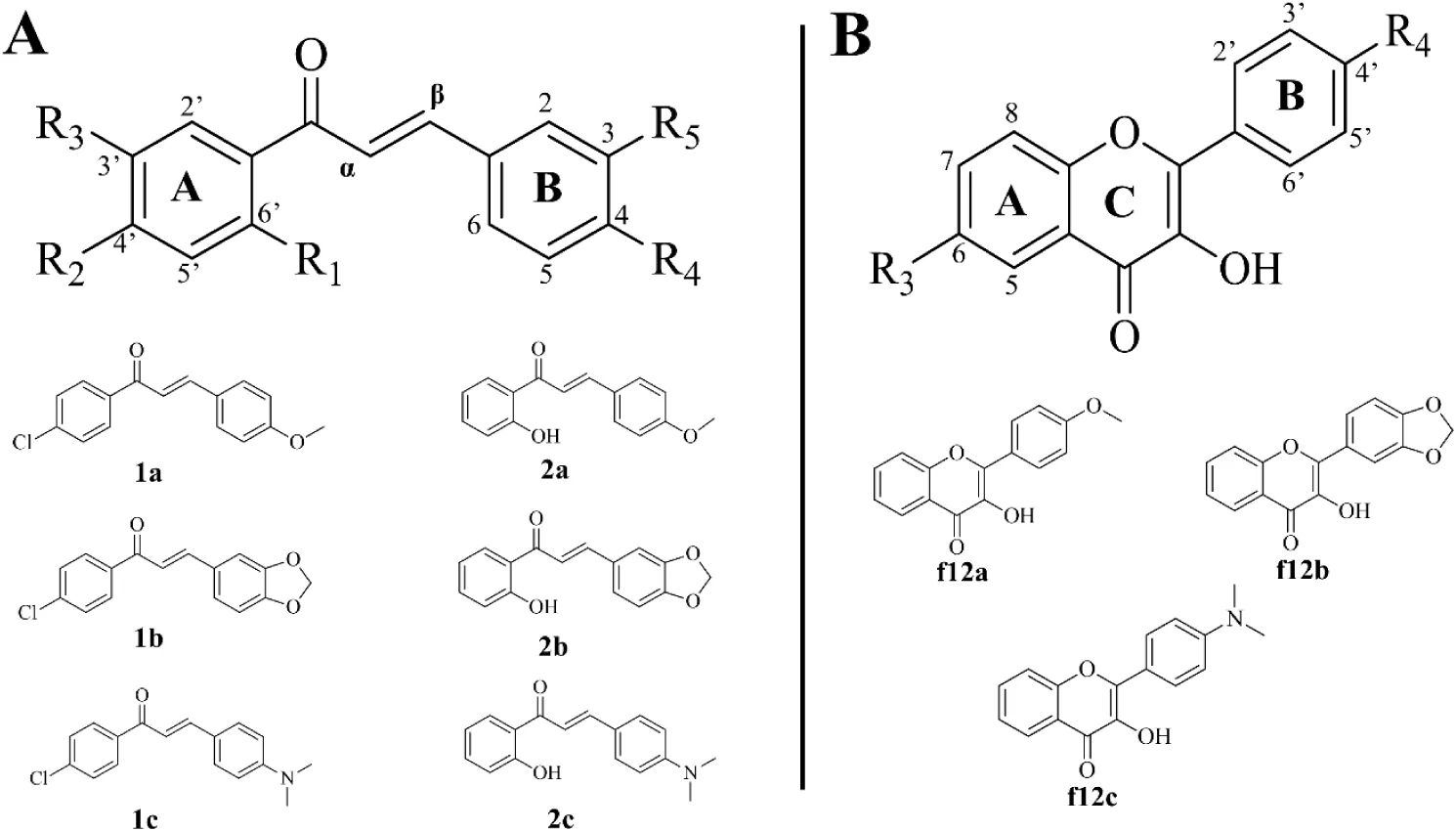
Chemical structures of the chalcone (**A**) and flavonol (**B**) scaffolds and their corresponding synthetic derivatives evaluated as inhibitors of cathepsins B and L.

The most potent CatB inhibitors, **2b** and **f12b**, feature a methylenedioxy moiety. This functional group can form π–π stacking interactions and/or hydrogen bonds. Such interactions are frequently observed in complexes with CatB and other enzymes overexpressed in tumors. Thus, the presence of a methylenedioxy group has been associated with enhanced inhibitory potency of both isolated and synthetic flavonoid derivatives targeting these enzymes.^61,62^ Despite the rare presence of flavonoids containing a methylenedioxy substituent in plants, a flavone bearing this functional group on the A ring was isolated from the leaves of *Dulacia egleri* and reported as a potent CatB inhibitor (IC_50_ = 14.88 ± 0.18 μM).^62^ This isolated flavone closely resembles our synthesized flavonol derivative **f12b**; however, the flavone and flavonol subclasses differ in a key aspect: flavonols possess a hydroxyl group at C3 of the C-ring. Since **f12b** is approximately 19-fold more potent, this phenolic substituent appears to contribute significantly to its high CatB inhibitory potency.

For certain flavonol activities, the hydroxyl group at C3 of the C-ring is recognized as part of the pharmacophore.^63,64^ In a previous study, Ramalho et al. (2015) evaluated flavonoids isolated from seven Cerrado plants for their inhibitory activity against CatB. The results indicated that quercetin was approximately three times more potent than luteolin.^41^ Later, molecular dynamics simulations with the flavonol gossypetin suggested the formation of a hydrogen bond between its C3 hydroxyl and Gly125 in the S2 subsite of CatB, supporting the hypothesis that its removal may reduce the inhibitory activity of compounds against this cysteine protease.^65^

The presence of hydroxyl substituents can also significantly influence the inhibitory activity of chalcones against CatB. Among a series of synthetic chalcones reported by Ramalho et al. (2013), compound (*E*)-1-(2H-1,3-benzodioxol-5-yl)-3-phenylprop-2-en-1-one (**4**) is particularly relevant for this analysis. Structurally analogous to chalcone **2b**, it also contains a methylenedioxy group on ring B, differing only by a hydrogen atom replacing the hydroxyl group at C6′.^43^ Notably, compound **4** is inactive against CatB, exhibiting only 29.6 ± 0.5% inhibition at 125 μM, thereby confirming the critical role of the hydroxyl group at this position. Although less pronounced, this observation is also evident among our compounds, as **2a**–**2c** exhibit higher potency than **1a** and **1c**, which contain a hydrogen atom at C6′, both in the presence and absence of heparin. While the methylenedioxy substituent on ring B exerts only a minor effect among **2a**–**2c**, the enhanced activity of **1b** (IC_50_ = 1.56 ± 0.04 μM), which carries this group, underscores the combined importance of the hydroxyl and methylenedioxy groups for CatB inhibition.

However, the limited number of flavonoid analogues evaluated in this study and reported in the literature hampers the establishment of a robust structure–activity relationship (SAR). This limitation is clearly reflected in the CatL inhibition data. The high inhibitory potency displayed by compounds **1a** and **f12a** (IC_50_ = 1.01 ± 0.05 and 1.12 ± 0.06 μM, respectively) suggests that the presence of a methoxy group on ring B, in both chalcone and flavonol derivatives, may contribute to enhanced CatL inhibition. On the other hand, the variability observed in the calculated IC_50_ values does not reveal a clear trend regarding the influence of A- and C-ring substituents on inhibitory activity. Therefore, further studies evaluating additional analogues bearing these substituents are required to determine a more definitive SAR for flavonoid derivatives.

### 2.3 Chalcone Analogues Reveal Significant Antiviral Activity against SARS-CoV-2 and Noncytotoxic Profile

The potent inhibitory activity of our compounds against CatB and CatL prompted us to also investigate their antiviral activity. Protocols to determine anti-SARS-CoV-2 activity require a high-biosafety BSL-3 research laboratory, which increases costs and depends on expert researchers with specialized training.^66^ Thus, alternative antiviral models have been developed for early stage screening prior to SARS-CoV-2 assays. Similar to SARS-CoV-2, Mouse Hepatitis Virus strain A59 (MHV-A59) belongs to the *Beta-coronavirus* genus. The close structural and replication features shared by these two coronaviruses make screening against MHV-A59 a valuable antiviral model for initial evaluation.^67,68^ As MHV-A59 is nonpathogenic to humans, antiviral assays can be conducted in BSL-2 laboratories, ensuring safety for analyzing larger libraries of compounds.^69^ The most effective candidates identified in this model can then be prioritized for confirmatory SARS-CoV-2 assays. Considering these advantages, we employed the MHV-based antiviral model as an initial screening platform.

Before conducting antiviral screening, solubility assays were performed for all nine chalcone and flavonol derivatives.^39,70^ After 48 h of incubation in aqueous medium with Vero CCL-81 cells, all three flavonol derivatives crystallized at 100 μM (Supporting Information, Figure S19A–C). Because of their poor aqueous solubility, the flavonol derivatives were therefore not considered for further antiviral evaluations, and the subsequent assays focused on compounds **1a**–**2c**.

Chalcones **2a** and **2b** consistently preserved L929 cell viability against MHV-A59 infection at all tested concentrations, indicating promising antiviral activity. At 100 μM, compounds **1c** and **2c** showed a similar protective effect, suggesting dose-dependent antiviral potential. In contrast, **1a** and **1b** did not fully maintain cell viability even at 100 μM, reflecting their lower antiviral efficacy compared to the other flavonoid derivatives (Figure 2A). Thus, we selected compounds **1c** and **2a**–**c** for subsequent experiments. However, the addition of the MHV-A59 viral suspension introduced additional challenges that were not evident in the preliminary solubility tests. Microscopic examination of the wells after the assays revealed that chalcone **2c** formed crystals at 100 μM (Supporting Information, Figure S19D).^78^ To ensure that all compounds were tested under the same experimental conditions, we maintained 100 μM as the highest concentration for cytotoxicity tests, while establishing 50 μM as the cutoff concentration for SARS-CoV-2 assays.

**Figure 2:**
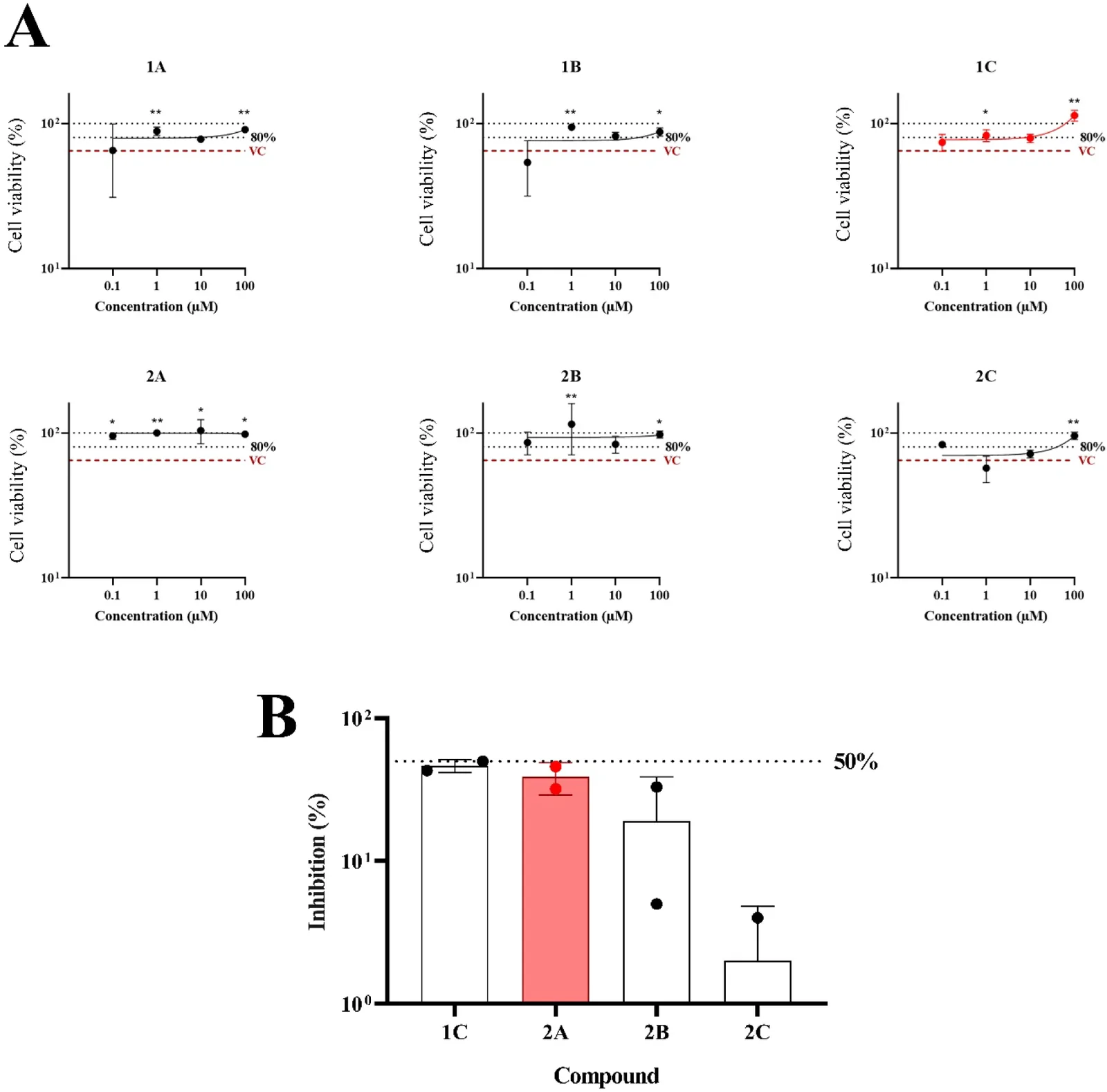
Antiviral screening of compounds **1a**–**2c** in L929 cells infected with MHV-A59 (A) and Vero CCL-81 cells infected with SARS-CoV-2 (B). (**A**) Displays the cell protection against MHV-A59 infection by compounds **1a**–**2c** performed at concentrations ranging from 100 to 0.1 μM. Data are shown as line graphs representing the mean ± standard error for each concentration (*x*-axis), with cell viability expressed as a percentage relative to untreated control cells (VC) on the *y*-axis. Statistical analyses were performed using Kruskal–Wallis test, with significance considered * *p* < 0.05 and ** *p* < 0.01. (**B**) Inhibition of SARS-CoV-2 viral load in Vero CCL-81 cells treated with chalcone derivatives (50 μM). Antiviral activity was determined by plaque reduction assay: viral titers (PFU/mL) were quantified by plaque counting, the untreated control cells (VC) titer was defined as 0% inhibition, and percentage inhibition was calculated as (1 – titer compound/titer untreated control cells) × 100. The dotted line marks the 50% inhibition threshold used to define hits. Data are shown as the mean ± SD. Compounds exhibiting the most promising antiviral effects are highlighted by the red line or red-colored bars. Both panels were performed as two independent experiments (*n* = 2), with three (A) and two (B) technical replicates per experiment.

Confirming their potential antiviral effects, **1c** and **2a** considerably reduced SARS-CoV-2 viral load. The most potent chalcone in inhibiting cathepsins B and L, compound **2b**, also showed a substantial ability to decrease viral titers. In contrast, compound **2c**, which exhibited a dose-dependent effect against MHV-A59, demonstrated lower efficacy against SARS-CoV-2 (Figure 2B).

However, despite the relevant antiviral results of **1c** and **2a**, the cytotoxicity of cathepsin-targeting compounds remains a crucial factor in identifying viable prototypes, since molecules that interact with these proteins generally exhibit low selectivity and may present unfavorable safety profiles.^71^ Assessment of viability in the Vero CCL-81 cell line, used as a model for SARS-CoV-2 infection, revealed that **2a** and **2b** had the safest profiles, even at the highest tested concentrations. A dose-dependent reduction in viability was observed for **1c** and **2c**, although it remained above 50% at 100 μM (Supporting Information, Figure S20A). Additionally, the compounds were tested against L929 cells, which were used for MHV-A59 infection during the primary antiviral screening and have been reported to be more sensitive and reliable than other cell lines for rigorous cytotoxicity assessment.^72,73^ Similarly to what was observed for Vero CCL-81 cells, **2a** exhibited the lowest cytotoxic effect against L929 cells at concentrations ranging from 0.1 to 100 μM (Supporting Information, Figure S20B). Due to the potent antiviral activity against SARS-CoV-2 and favorable cytotoxicity profile, compound **2a** emerged as the most promising candidate in the preliminary screening.

To further characterize the antiviral properties of the flavonoid derivatives, dose–response assays were carried out to quantitatively determine the concentration required for each compound to achieve a 50% reduction in viral titer (IC_50_). The results confirmed compound **2a** as the most potent anti-SARS-CoV-2 agent (IC_50_ = 1.05 μM) and highlighted **2b** (IC_50_ = 2.12 μM) as another promising bioactive molecule (Figure 3). These compounds presented high Selectivity Index (SI) values, with both **2a** (SI = 95.23) and **2b** (SI = 47.17) exhibiting selective antiviral activity relative to cytotoxic effects on host cells. This index value was lower for compound **1c** (SI = 8.29), despite its good potency against SARS-CoV-2 (IC_50_ = 12.06 μM) (Supporting Information, Figures S20A and S20B). Chalcone **2c** exhibited the weakest antiviral response, as it did not reduce the viral titer by 50% even at the highest tested concentration of 50 μM (Figure 3). Although this result was expected based on preliminary screening results, it remains noteworthy, as this chalcone is structurally analogous to **2a** and **2b**. To explain these differences in potency, water solubility appears to be a key factor. Compound **2c**, obtained as a crystalline solid during synthesis, exhibited an ordered three-dimensional packing that may contribute to higher thermodynamic stability and reduced solubility compared with the amorphous solids **2a** and **2b**.^74^ Although no crystal formation was observed by microscopic analysis at a concentration of 50 μM, the presence of undetected insoluble particles in the medium cannot be excluded. Therefore, the lower antiviral activity observed for **2c** may be related to limited compound availability in the assay medium; however, additional studies will be required to experimentally confirm this hypothesis. Considering that strategies such as amorphous dispersions and nanoformulations can improve the solubility and bioactivity of compounds with biopharmaceutical limitations,^75^ further optimization approaches may be explored for this compound in future studies.

**Figure 3:**
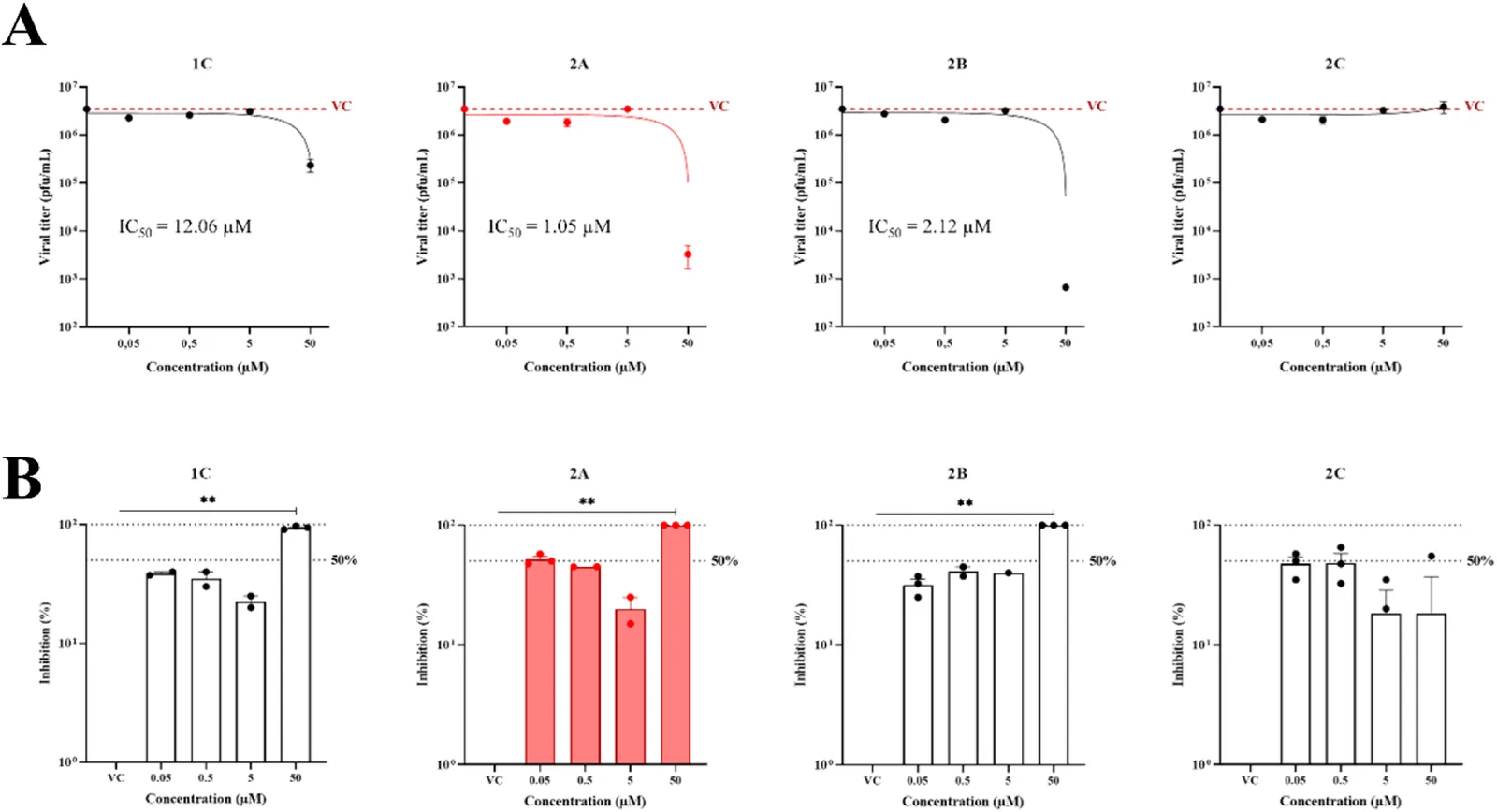
Dose–response assays of compounds **1c**–**2c** in Vero CCL-81 cells infected with SARS-CoV-2. (**A**) Displays the SARS-CoV-2 viral titer reduction at concentrations ranging from 50 to 0.05 μM. Data are shown as line graphs representing the mean ± standard error of the mean (SEM) for each concentration (*x*-axis), with viral titer expressed as a percentage relative to untreated control cells (VC) on the *y*-axis. (**B**) Results are displayed as bar graphs representing mean and standard errors of the mean (SEM) for cells treated chalcone derivatives after 48 h postinfection. Statistical analyses were performed using the Kruskal–Wallis test followed by Dunn’s multiple comparisons test. Differences were considered statistically significant at *p* < 0.05. In the graphs, ** indicates *p* < 0.01. The compound with lowest IC_50_ value is highlighted by the red line or red-colored bars. These experiments were performed in three independent replicates (*n* = 3).

### 2.4 Inhibition Data on Viral Enzymes Suggest CatB/CatL Inhibition as a Potential Mechanism Underlying the Anti-SARS-CoV-2 Activity of Chalcones **2a** and **2b**

As observed for compound **2c**, high enzymatic potency does not always translate into the desired pharmacological effect. In a series of 21 peptidomimetics, Flury et al. (2024) identified two compounds with high inhibitory potency against cathepsins, exhibiting nanomolar K_*i*_ values. Unexpectedly, *in vitro* evaluation of these molecules against SARS-CoV-2 revealed that the most potent cathepsin inhibitors had no antiviral activity, whereas another peptidomimetic analogue with over 100-fold lower enzymatic potency exhibited an EC_50_ of 38.4 nM.^18^ Flavonoid compounds, widely studied for their antiviral effect, have been shown to inhibit SARS-CoV-2 through two distinct mechanisms: interference with viral entry into host cells and inhibition of viral replication.^76^ Therefore, considering other antiviral mechanisms that could potentially be targeted by flavonoid-based compounds is essential to better understand their anti-SARS-CoV-2 effects and to guide future structural optimization.

The SARS-CoV-2 main protease (M^pro^) is critical for viral replication and one of the most studied molecular targets for COVID-19 drug development. Its unique substrate specificity and lack of close human homologues make it an attractive target, validated by the development of drugs currently in the clinic.^77,78^ Several M^pro^ inhibitors, including flavonoid derivatives, have been reported.^79,80,81^ In particular, synthetic chalcones bearing a fluorine atom on the A-ring have shown potent M^pro^ inhibition and significant efficacy in reducing SARS-CoV-2 replication.^82^ However, growing concern about resistance to its current inhibitors has motivated recent structural and biochemical studies to identify multitarget compounds. Among them, one study demonstrated that cathepsin inhibitors can also interact with M^pro^, as evidenced by the cocrystallization of synthetic compounds with this cysteine protease.^83^ This diverse evidence in the literature prompted us to investigate the SARS-CoV-2 M^pro^ as an additional antiviral target for the potent compounds **1c**, **2a**, and **2b**.

Enzymatic assays against M^pro^ revealed that none of the four chalcones (**1c**–**2c**) were effective, with inhibition percentages ranging from 15 to 34% (Table 3). SAR studies of chalcones as M^pro^ inhibitors indicate that electronegative substituents, particularly fluorine on the A-ring, mediate interactions with amino acid residues in the P1′/P1 pockets. The presence of these electronegative substituents also led some chalcones to be recognized as covalent inhibitors of M^pro^. When both the A- and B-rings bear electronegative substituents, an electron-withdrawing effect is induced, increasing the electrophilicity of the β-carbon in the chalcone’s Michael acceptor and, consequently, its reactivity in nucleophilic addition with Cys145.^82^ On chalcones **2a**–**2c**, the phenolic hydroxyl on the A-ring functions as an electron-donating group via π-delocalization, whereas the chloride on the A-ring of **1c**, despite its electronegativity, exerts only a modest electron-withdrawing effect compared to fluorine due to its larger atomic radius.^84,85^ The structural differences of compounds **1c**–**2c** compared to previously reported chalcones as M^pro^ inhibitors may account for their low inhibitory activity against this protease.

**Table 3:** Inhibitory Effect of Chalcone Derivatives on SARS-CoV-2 Main Protease

Compounds	% inhibition of M^a^ ^pro^
1a	19 ± 3
2a	27 ± 3
2b	15 ± 3
2c	34 ± 2

a Percentages were calculated at 20 μM, and data are presented as mean ± standard error of the mean (SEM) from two independent experiments, each performed in triplicate, following preincubation of the compounds with the enzyme.

The low inhibitory potency against M^pro^ does not exclude the possibility that these chalcones may target other viral proteins. To validate cathepsin inhibition as the main mechanism responsible for their anti-SARS-CoV-2 activity, virucidal tests were performed. This analysis, performed without a cellular model, directly exposes the virus to the compounds. If an anti-SARS-CoV-2 compound does not reduce viral titers under these conditions, it suggests that its action may involve viral entry pathways through interactions with host proteins, rather than interfering with the viral life cycle. For all four chalcones (**1c**–**2c**), the reduction in viral load was not statistically significant (Figure 4). This nonvirucidal effect is consistent with the hypothesis that the antiviral activity of compounds **1c**, **2a**, and **2b** may involve dual CatB/CatL inhibition, while the low dose–response observed for **2c** may be related to an unfavorable pharmacokinetic profile, although further studies are needed to confirm this possibility. Collectively, these findings provide initial insights that may guide the development of more effective chalcone-based leads against COVID-19.

**Figure 4:**
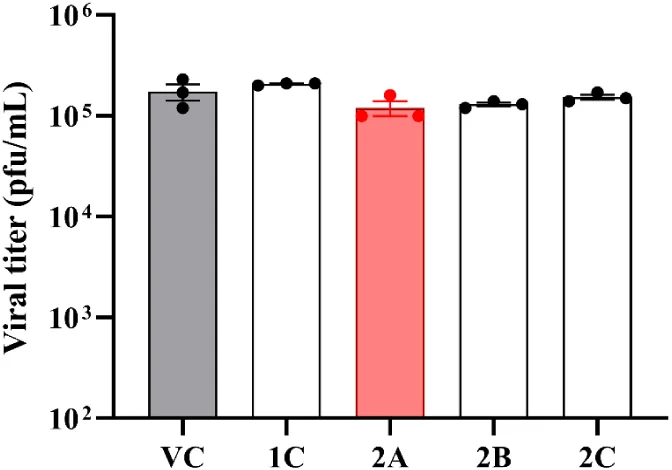
Compounds **1c**–**2c** do not exhibited virucidal activity against SARS-CoV-2 infection. Results are shown as bar graphs representing the mean ± standard error of the mean (SEM) of viral titers, expressed in plaque-forming units (PFU). Statistical analyses were performed using the Kruskal–Wallis test followed by Dunn’s multiple comparisons test. No significant differences were observed (*p* > 0.05). The virucidal effect of the compound with the lowest IC_50_ value against SARS-CoV-2, chalcone **2a**, is highlighted by the red-colored bar. These experiments were performed in three independent replicates (*n* = 3).

## 3 Conclusion

Enzymatic assays against CatB and CatL identified nine compounds with potent inhibitory activity against both cysteine proteases, which were subsequently characterized as linear noncompetitive inhibitors. Structure–activity relationship analysis indicated that electron-donating and polar substituents on the phenolic rings of the flavonol and chalcone derivatives were crucial for activity. Through initial screening against MHV-A59, compounds **1c**–**2c** were revealed as potential antiviral agents, preserving cell viability after 48 h of infection. This evidence was confirmed in antiviral assays against SARS-CoV-2, in which compound **2a** demonstrated strong potency in reducing viral load (IC_50_ = 1.05 μM), while **2b** (IC_50_ = 2.12 μM) also proved to be a promising antiviral prototype. On the other hand, no antiviral effect was observed for **2c**, which was likely associated with its low water solubility. Cell viability assays in Vero CCL-81 and L929 cells showed low cytotoxicity, resulting in high selectivity indices for **2a** (SI = 95.23) and **2b** (SI = 47.17). Additionally, evaluation of alternative molecular targets demonstrated that the chalcones did not inhibit SARS-CoV-2 M^pro^ and did not exhibit virucidal activity. These findings support the involvement of CatB/CatL inhibition in the antiviral activity of the chalcone derivatives, although further mechanistic studies, including rescue experiments and comparisons with selective CatB and CatL inhibitors, are required to confirm their mechanism of antiviral activity. Furthermore, although the present study evaluated the antiviral activity of the compounds against the SARS-CoV-2 Wuhan strain, their activity against genetically distinct variants, including Omicron, remains to be experimentally confirmed. Given the greater contribution of the endosomal cathepsin-mediated entry pathway reported for Omicron, evaluation of these compounds against such variants may provide insight into their antiviral spectrum. Therefore, studies involving circulating and emerging SARS-CoV-2 variants are warranted to assess the spectrum of their antiviral activity.

## 4 Experimental Section

### 4.1 Chemistry

The procedure described by Lourenço et al. (2023)^39^ was followed to obtain all synthetic flavonol and chalcone derivatives used in this study. To confirm the structures of the synthesized compounds, experimental NMR data, retention factor (R_*f*_) values and melting points were compared with reported literature.^34,39^ All reagents were obtained from Sigma-Aldrich and used as received unless otherwise noted while solvents used in reactions and recrystallization were purified and dried according to literature procedures. Reaction progress was monitored by thin-layer chromatography (TLC) on silica gel 60 F254 precoated plates (Merck), with visualization under UV light (254 or 360 nm) and staining with molybdophosphoric acid (10% w/v) or sulfuric vanillin solution (0.5 g vanillin in 100 mL of H_2_SO_4_/MeOH, 40:10 v/v). Final compounds were purified by recrystallization from ethanol (EtOH). ^1^H and ^13^C NMR spectra were recorded on Bruker Avance DPX-300 or Ascend 500 spectrometers. Chemical shifts (δ) are reported in ppm relative to residual solvent signals (CDCl_3_, δ 7.24; DMSO-*d*_6_, δ 2.50). Signal multiplicities are designated as s (singlet), d (doublet), dd (doublet of doublets), tp (triplet of doublets), t (triplet), dt (doublet of triplets), or m (multiplet). Coupling constants (*J*) are reported in Hz. Mass spectra were acquired using a Bruker Autoflex MALDI-TOF/TOF mass spectrometer equipped with a smartbeam-III laser operating at a repetition rate of up to 2 kHz. Spectra were recorded in reflector mode in positive ion mode over the *m*/*z* range 200–260. The number of laser shots was not specified. No MS/MS fragmentation was performed. Instrument parameters were controlled using FlexControl 3.3 software. All final compounds were characterized by NMR spectroscopy, MALDI-TOF, and TLC analysis. These analyses were consistent with the expected structures and showed no detectable impurities under the evaluated conditions. Spectroscopic data and corresponding ^1^H and ^13^C NMR spectra for all compounds are provided in the Supporting Information.

### 4.2 Expression and Purification of Cathepsins B and L

Cathepsins B and L were expressed in *Pichia pastori* *s* containing the pPICZ9 vector, according to the methodology previously described by Linnevers et al. (1997).^86^ Culture media were prepared according to the manufacturer’s protocol (EasySelect Pichia Expression Kit, Invitrogen Life Technologies) under a laminar flow hood. The expression products (supernatants) were concentrated by tangential filtration process on a 10 kDa DMEMbrane in Millipore Labscale TFF System equipment. The concentrates were dialyzed using Spectrum – SpectraPor 2 DMEMbrane (0.22 μM) in 25 mM sodium acetate buffer for zymogen activation (pH = 5.5). Dialysis was performed overnight, changing the buffer every hour for the first 3 h. The material resulting from the dialysis was subjected to ion exchange chromatography on a HiTrapTM SP FF column (5 mL) connected to an AKTA Purifier (GE LifeSciences). The material was applied to the HiTrapTM SP FF column previously equilibrated with 100 mM sodium acetate buffer (pH 5.5). The adsorbed proteins were eluted in 100 mM sodium acetate buffer in a linear gradient of NaCl (0–1 M) at constant flow of 1 mL/min for 50 min. Aliquots of 1 mL were collected and stored under refrigeration for subsequent monitoring of catalytic activity. Measurements were performed on a Molecular Devices plate reader after incubating the enzymes for 5 min in 100 mM sodium acetate buffer (pH 5.5) containing 2 mM DTT and Z-FR-AMC (18.5 μM and 9.25 μM for cathepsins B and L, respectively). The hydrolysis of Z-FR-AMC was monitored at λ_Ex_ = 360 nm and λ_Em_ = 480 nm.

### 4.3 Enzyme Titration

The molar concentration of human recombinant cathepsins B and L was determined by titration of the active site with the irreversible inhibitor of cysteine peptidases E-64, in a Hitachi F-2700 spectrofluorometer, thermostated at 37 °C, with excitation and emission slits adjusted to 5 and 5 nm (700 V), λ_Ex_ = 360 nm and λ_Em_ = 480 nm. Cathepsins B and L were incubated for 5 min in 100 mM sodium acetate buffer (5 mM EDTA, 20% glycerol (v/v), pH 5.5, and 2 mM DTT). The incubation was performed in a 1 mL quartz cuvette placed in the spectrofluorometer chamber under constant stirring. Subsequently, the Z-FR-MCA substrate was added (in concentrations of 18.5 μM for cathepsin B and 9.25 μM for cathepsin L), for monitoring the enzymatic activity, followed by the addition of increasing concentrations of the E-64 inhibitor. The enzymatic activity velocity values obtained in UAF/min (Arbitrary Fluorescence Units per minute) were analyzed using Grafit 5.0.13 software.^87^

### 4.4 Determination of IC_50_ Values for Chalcone and Flavonol Derivatives against CatB and CatL

Cathepsin L was assayed in a reaction buffer of 100 mM sodium acetate, containing 100 mM NaCl, 5 mM EDTA and 20% glycerol (v/v) at pH 5.5. Cathepsin B was assayed in same buffer supplemented with 0.0.1% Triton X-100. The enzymes were preincubated in the presence of 3 mM DTT for 5 min at 37 °C in a 1 mL final volume with constant stirring. The enzyme activities were monitored using fluorogenic probe Z-FR-AMC (18.5 μM and 9.25 μM for cathepsin B and L, respectively), and the fluorescence was determined by spectrofluorometry using fluorometer RF6000 (Shimadzu, Tokyo, Japan) set to λ_Ex_ = 360 nm and λ_Em_ = 480 nm. The enzymes were analyzed under two conditions: in the absence and presence of 10 μM heparin. Heparin was utilized as a well-established in vitro surrogate for heparan sulfate and extracellular matrix glycosaminoglycans. This specific concentration was chosen based on previous mechanistic studies that employed similar micromolar levels to investigate the modulation of papain-family cysteine proteases by heparin.^29,88^ Importantly, 10 μM aligns with the reported dissociation constants (K_d_ ≈ 17–18 μM) for heparin–cathepsin B and heparin–cathepsin L-like protease interactions,^29,88^ ensuring a physiologically meaningful fractional occupancy of the enzyme by the polysaccharide under our assay conditions. The inhibitory potential (IC_50_) was performed by progressively increasing the concentration of the compounds and the values were calculated by nonlinear regression. The data were analyzed by Grafit 5.0.13 software^87^ using the equation below (eq 1).

1 $$y=\frac{100\%}{1+{\left(\frac{x}{IC_{50}}\right)}^{S}}$$

### 4.5 Enzyme Kinetics and Determination of the Mechanism of Inhibition

Assays with CatB and CatL were performed using the same reaction buffer described in **4.4**. Both enzymes were preincubated in the presence of 3 mM DTT for 10 min at 37 °C in a final volume of 1 mL with constant stirring. Studies of the inhibition kinetics of cathepsins B and L were performed in the absence and presence of 10 μM heparin. As detailed in Section 4.4, this concentration was previously employed in mechanistic studies of cathepsin–heparin interactions^56,88^ and is sufficient to promote stable, physiologically relevant enzyme–heparin complex formation based on reported affinities.^29^ Concentrations of the substrate Z-FR-AMC ranged from 2 to 85.5 μM. The assays were performed in the absence and presence of compounds in 4 different concentrations. Fluorescence was monitored by spectrofluorometry using an RF6000 fluorometer (Shimadzu, Tokyo, Japan) set to λ_Ex_ = 360 nm and λ_Em_ = 480 nm. All kinetic assays were performed in duplicate. Inhibition constants were determined by using different equations depending on the inhibition mechanism.^89,90^ Activity rate and substrate concentration data generated rectangular hyperbolic profiles, which were linearized using Lineweaver–Burk (double-reciprocal) plots. Secondary replots of slope vs inhibitor concentration and intercept vs inhibitor concentration provided the K_*i*_ and αK_*i*_ parameters, respectively.^89^

### 4.6 Cells and Viruses

Mouse (*Mus musculus*) clone 929 connective tissue L cells (L-929, derivative of Strain L) NCTC ATCC were used for the initial antiviral screening. African green monkey (*Cercopithecus aeothiops*) kidney cells ATCC CCL-81 were also used. Both cells were cultured and maintained in Minimum Essential Medium Eagle (MEM) (Sigma-Aldrich, USA) (Sigma-Aldrich, USA), containing 5% fetal bovine serum (FBS) (Cultilab, Brazil) and a cocktail of antibiotics streptomycin (Sigma-Aldrich, USA [100 μg/mL]), potassium penicillin (Sigma-Aldrich, USA [100 U/mL]), and amphotericin B (Bristol-Myers Squibb, Brazil, [2.5 μg/mL]). The cultures were incubated in a humidified oven at 5% CO_2_ and 37 °C. Murine hepatitis virus strain MHV-A59 used for the initial antiviral screening was purchased from ATCC (Manassas, Virginia, USA) while the SARS-CoV-2 Wuhan-Hu-1 isolate was used in this study. Antiviral activity assays were performed in the biosafety level 3 (BSL-3) laboratory at the Institute of Biological Sciences of UFMG, Brazil.

### 4.7 Viral Propagation

SARS-CoV-2 and MHV-A59 were propagated in Vero CCL-81 and L-929 monolayers, respectively, using 175 cm^2^ culture flasks. Cells were infected with a multiplicity of infection (MOI) of 0.01 and incubated at 37 °C in a 5% CO_2_ atmosphere until cytopathic effect was observed. The supernatant was collected and centrifuged (Multifuge centrifuge, X3R, Thermo Scientific, USA) for 15 min at 2,500 rpm at 25 °C and stored at −80 °C until use.^91^ Viral titration was performed using the Dulbecco plate assay.^92^

### 4.8 Evaluation of the Antiviral Activity of Synthetic Flavonoids against *In Vitro* Infection by MHV-A59

L-929 cells were seeded in 96-well plates with MEM 5% FBS medium. After 24 h of incubation (37 °C and 5% CO_2_), the cells were infected with an MHV-A59 inoculum with an MOI of 0.01. The infected cells were incubated at 37 °C with 5% CO_2_ atmosphere for 1 h for adsorption. The supernatants were then discarded, and the compounds previously solubilized in culture medium were added. The treatments were made at concentrations ranging from 100 μM to 0.1 μM. Cell controls, cell death control (Triton X-100 10% v/v), and vehicle control (DMSO 0.5% v/v) were included in the same assay plate. Subsequently after a period of 16 h of incubation all the supernatant was removed and 100 μL of a 5% MEM, 10% alamarBlue solution was added to each well. The plates were protected from light and incubated for 4 h at 37 °C with 5% CO_2_. A Thermo Scientific Multiskan GO spectrophotometer was used to read the absorbance (λ = 577 nm). The results, expressed as a percentage of cell viability, were analyzed and graphs plotted in GraphPad Prism 9.0. The assays were performed in triplicate.

### 4.9 Evaluation of the Antiviral Activity of Synthetic Flavonoids against *In Vitro* Infection by SARS-CoV-2

Vero CCL-81 cells were seeded in 24-well plates at a density of 1 × 10^5^ cells per well and cultured until they reached approximately 90% confluence. The cell monolayers were infected with 100 plaque-forming units (PFU) of SARS-CoV-2 and 50 μM of each compound for 1 h at 37 °C. After 1 h of adsorption, the cell monolayers were covered with medium 199 containing 2% SFB and 1% CMC. After 72 h of incubation at 37 °C, the cells were fixed with 10% formaldehyde for 40 min and stained with 1% crystal violet.^93^ The plates were visualized and counted manually using a white light transilluminator, and the percentage of viral inhibition was calculated following eq 2:

2 $$\%viral\;inhibition:100-\frac{\left(PFU\;number\;of\;treated\;cells\;\times 100\right)}{PFU\;number\;of\;viral\;control}$$

### 4.10 Dose–Response Assay for SARS-CoV-2 Infection

Vero CCL-81 cells were plated in 24-well plates at a density of 1 × 10^5^ cells/well and incubated at 37 °C for 24 h. The cells were then infected with SARS-CoV-2 at an MOI of 0.01. For viral adsorption, the cells were kept in a humidified incubator at 37 °C and 5% CO_2_ for 1 h. After adsorption, the supernatant was removed, and the cells were immediately treated with different concentrations of the tested compounds, ranging from 50 to 0.05 μM, for 48 h. Following the treatment period, cell suspensions were collected to carry out viral titration.^94^ The curves for evaluating the antiviral dose–response activity of the compounds were plotted using the viral titer results, and the calculation of the absolute IC_50_ was based on the ideal nonlinear regression for interpolating sigmoidal curves. The calculations were carried out using GraphPad Prism software (version 9.0).

### 4.11 Cytotoxicity on Vero CCL-81 and L-929 Cells

The cytotoxicity assays were performed based on the protocol reported by Reis et al. (2025), with minor modifications.^91^ L929 and Vero CCL-81 cells were seeded in 96-well microplates (2 × 10^4^ cells/well). Subsequently, 200 μL/well of Minimum Essential Medium Eagle (MEM) with 1% FBS containing different compounds at concentrations ranging from 100 to 0.1 μM was added to the wells. Cell controls, a cell death control (10% v/v Triton X-100), and a vehicle control (0.5% v/v DMSO) were included on the same assay plate. After the incubation, the supernatant was discarded, and alamarBlue reagent (Thermo Fisher Scientific, USA) (10%) was added to the cells and incubated at 37 °C with 5% CO_2_ for 4 h in the dark. All conditions were performed in internal triplicate and reproduced in two independent experiments (n = 6). Cell viability was assessed by measuring absorbance at 577 nm using a Multiskan GO UV–Vis spectrophotometer (Thermo Fisher Scientific, USA), and results, expressed as percentages relative to control, were analyzed statistically using GraphPad Prism 9.0.

### 4.12 Evaluation of SARS-CoV-2 M^pro^ Inhibition by Chalcone Derivatives

Enzymatic assays were conducted in black flat-bottom 96-well microplates, and fluorescence was measured at 320/405 nm using a Cytation 5 Cell Imaging Multi-Mode Reader (Biotek, Vermont, USA). SARS-CoV-2 M^pro^ activity was assessed using 10 μM of the fluorogenic substrate MCA-AVLQSGFR-Lys­(Dnp)-Lys-NH_2_ trifluoroacetate (Sigma-Aldrich, USA and Canada). Proteolysis of the substrate was performed in 20 μM Tris-HCl buffer (pH 7.5) containing 1 mM EDTA, 0.5% DMSO, 5 mM DTT, 0.01% Triton X-100, and 20 nM M^pro^, in a total reaction volume of 100 μL. The compounds were preincubated with the enzyme for 10 min, followed by the addition of a substrate-containing solution, and the reaction rate was immediately measured for 5 min at 37 °C. Due to the low solubility of the compounds in aqueous medium, the screening was performed at 20 μM, and their relative inhibition was calculated based on the initial reaction rate in the presence of DMSO. Expression and purification of recombinant SARS-CoV-2 M^pro^ were carried out according to a previously established protocol. All experiments were conducted in triplicate and repeated in two independent assays.^95^

### 4.13 Investigation of the Virucidal Potential of Synthetic Flavonoids SARS-CoV-2

The compounds were incubated with 10^5^ plaque-forming units (PFU) SARS-CoV-2 for 1 h at 37 °C, at 50 μM. In parallel, SARS-CoV-2 was added to DMEM and kept as a negative control. After 1 h of incubation, the solutions were diluted from 10^–1^ to 10^–6^ to perform the viral titration.

## Supplementary Material



## Data Availability

All data sets generated for this study are included in the article and its Supporting Information.

## Notes

Mechanisms of cathepsin B/L inhibition by compounds **1a**–**f12b** in the presence or absence of 10 μM heparin; microscopic analysis of the wells in the solubility assay for compounds **f12a**, **f12b**, **f12c**, and **2c**; cytotoxicity assays for compounds **1a**–**2c** at concentrations ranging from 0.01 to 100 μM against Vero CCL-81 and L929 cells; spectroscopic data of synthetic compounds (PDF)
