# Supplementary material for: Synthetic Flavonoid Analogues as CatB/CatL Inhibitors with Potential Anti-SARS-CoV‑2 Activity
Source: ACS Omega. 2026 Oct 6;11(41):63677–91. doi: 10.1021/acsomega.6c07800 (PMC13688569; doi:10.1021/acsomega.6c07800)
Supplement: Supplementary file 1 [file ao6c07800_si_001.pdf]

Synthetic Flavonoid Analogues as CatB/CatL Inhibitors with Potential Anti-SARS-CoV-2 Activity

Estela M. G. Lourenço[1,4], Marli de F. C. Emiliano[2], Silvana Ribeiro[2], Felipe A. Clarindo[3], Luan d. S. Vianna[2], Thaís de F. S. Moraes[3], Erik V. de S. Reis[3], Aline A. de Souza[2], Luiza V. Barreto[1], Mauricio F. M. Machado[2], Adilson Beatriz[4], Letícia G. de Pontes[1,5], Mariana O. de Godoy[6], Glaucius Oliva[6], Rafael V. C. Guido[6], Jordana G. A. Coelho-dos-Reis[3], Wagner A. de S. Judice[2], Dênis P. de Lima[4] and Rafaela S. Ferreira[1,*]

[1] Laboratório de Modelagem Molecular e Planejamento de Fármacos (LMMPF), Departamento de Bioquímica e Imunologia, Instituto de Ciências Biológicas, Universidade Federal de Minas Gerais, Av. Antônio Carlos, 6627, Pampulha, 31270-901, Belo Horizonte, MG, Brazil

[2] Interdisciplinary Center for Biochemical Research (CIIB), University of Mogi das Cruzes (UMC), Av. Dr. Cândido Xavier de Almeida Souza, 200, Centro Cívico, 08780-911, Mogi das Cruzes, SP, Brazil

[3] ProsPeRa Research Group, Grupo de Prospecção e Pesquisas em Resposta Imune e Antivirais, Departamento de Microbiologia, Instituto de Ciências Biológicas, Universidade Federal de Minas Gerais, Av. Antônio Carlos, 6627, Pampulha, 31270-901, Belo Horizonte, MG, Brazil

[4] Laboratório de Síntese e Transformação de Moléculas Orgânicas (SINTMOL), Instituto de Química, Universidade Federal de Mato Grosso do Sul, Avenida Senador Filinto Müller, 1555, Cidade Universitária, 79074-460, Campo Grande, MS, Brazil

[5] Laboratório de Biodados, Departamento de Bioquímica e Imunologia, Instituto de Ciências Biológicas, Universidade Federal de Minas Gerais, Av. Antônio Carlos, 6627, Pampulha, 31270-901, Belo Horizonte, MG, Brazil

[6] Instituto de Física de São Carlos (IFSC), Universidade de São Paulo (USP), Av. Trabalhador São-carlense, 400, 13566-590, São Carlos, SP, Brazil

*** Correspondence:**

Rafaela Salgado Ferreira

rafaelasf@icb.ufmg.br

Summary

Figure S1: Mechanism of cathepsin B inhibition by **1a** in the absence and presence of 10 µM heparin.......... 3
Figure S2: Mechanism of cathepsin B inhibition by **1b** in the absence and presence of 10 µM heparin.......... 4
Figure S3: Mechanism of cathepsin B inhibition by **1c** in the absence and presence of 10 µM heparin.......... 5
Figure S4: Mechanism of cathepsin B inhibition by compound EM-2A in the absence and presence of 10 µM heparin.......... 6
Figure S5: Mechanism of cathepsin B inhibition by 2b in the absence and presence of 10 µM heparin.......... 7
Figure S6: Mechanism of cathepsin B inhibition by **2c** in the absence and presence of 10 µM heparin.......... 8
Figure S7: Mechanism of cathepsin B inhibition by **f12a** in the absence of heparin.......... 9
Figure S8: Mechanism of cathepsin B inhibition by **f12b** in the absence of heparin. 10
Figure S9: Mechanism of cathepsin B inhibition by **f12c** in the absence and presence of 10 µM heparin.......... 11
Figure S10: Mechanism of cathepsin L inhibition by **1a** in the absence and presence of 10 µM heparin.......... 12
Figure S11: Mechanism of cathepsin L inhibition by **1b** in the absence and presence of 10 µM heparin.......... 13
Figure S12: Mechanism of cathepsin L inhibition by **1c** in the absence and presence of 10 µM heparin.......... 14
Figure S13: Mechanism of cathepsin L inhibition by **2a** in the absence and presence of 10 µM heparin.......... 15
Figure S14: Mechanism of cathepsin L inhibition by **2b** in the absence and presence of 10 µM heparin.......... 16
Figure S15: Mechanism of cathepsin L inhibition by 2c in the absence and presence of 10 µM heparin.......... 17
Figure S16: Mechanism of cathepsin L inhibition by f12a in the absence and presence of 10 µM heparin.......... 18
Figure S17: Mechanism of cathepsin L inhibition by **f12b** in the absence and presence of 10 µM heparin.......... 19
Figure S18: Mechanism of cathepsin L inhibition by **f12c** in the absence and presence of 10 µM heparin.......... 20
Figure S19: Microscopic analysis of the wells in the solubility assay for compounds **f12a** (**A**), **f12b** (**B**), **f12c** (**C**), and **2c** (**D**). Vero-CCL81 cells (3×10^4 cells/well) were seeded in 96-well plates and incubated in Minimum Essential Medium (MEM) supplemented with 1% FBS during the assay. 21
Figure S20: Cytotoxicity assays for compounds **1a**–**2c** at concentrations ranging from 0.01 to 100 µM against Vero CCL-81 (**A**) and L929 (**B**) cells. All conditions were performed in internal triplicate and reproduced in two independent experiments (n = 6). 22

Spectroscopic data.......... 23
NMR spectra (Figures S21-S38).......... 23

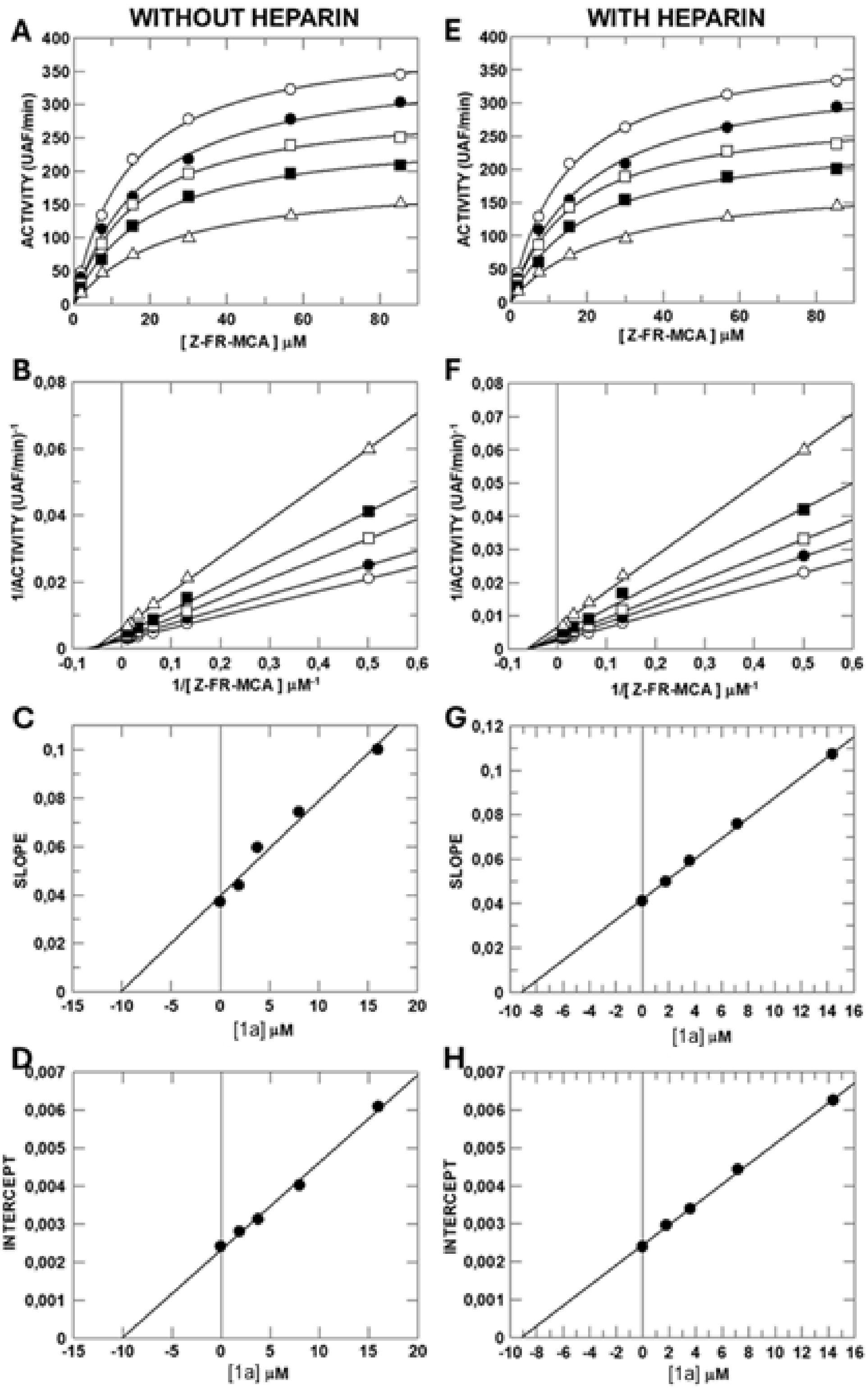

Figure S1: Mechanism of cathepsin B inhibition by **1a** in the absence and presence of 10 μM heparin. (A, E) Michaelis-Menten plots; (B, F) Double-reciprocal (Lineweaver-Burk) plots; (C, G) Slope replots;(D, H) Intercept replots. In A and B:○→ control; ●→ 1.9 μM; □→ 3.8 μM; ■→ 8 μM; △→ 16 μM. In E and F: ○→ control; ●→ 1.8 μM; □→ 3.6 μM; ■→ 7.2 μM; △→ 14.4 μM

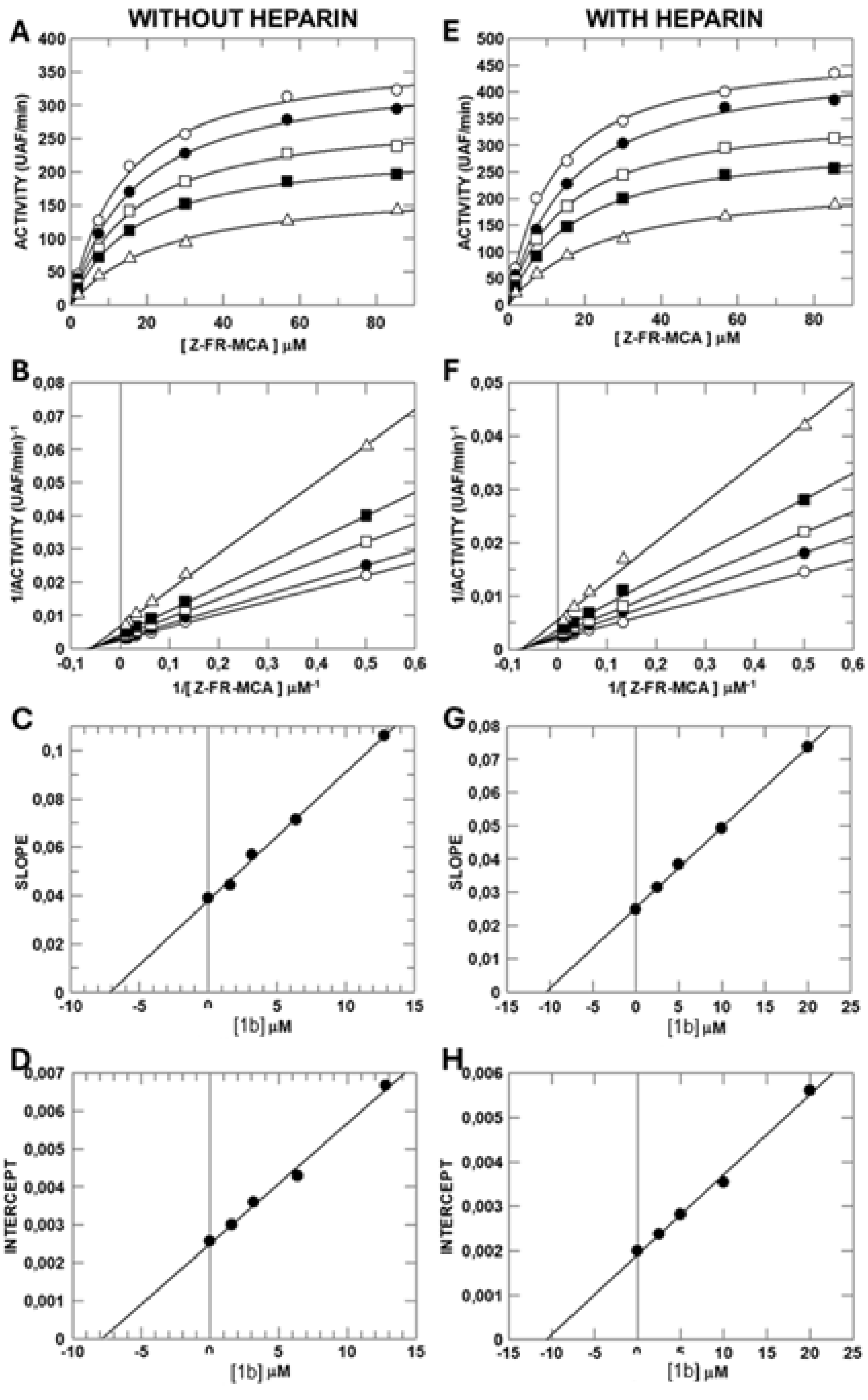

Figure S2: Mechanism of cathepsin B inhibition by **1b** in the absence and presence of 10 µM heparin. (A, E) Michaelis-Menten plots; (B, F) Double-reciprocal (Lineweaver-Burk) plots; (C, G) Slope replots; (D, H) Intercept replots. In A and B: ○→ control; ●→ 1.6 µM; □→ 3.2 µM; ■→ 6.4 µM; △→ 12.8 µM. In E and F: ○→ control; ●→ 2.5 µM; □→ 5 µM; ■→ 10 µM; △→ 20 µM.

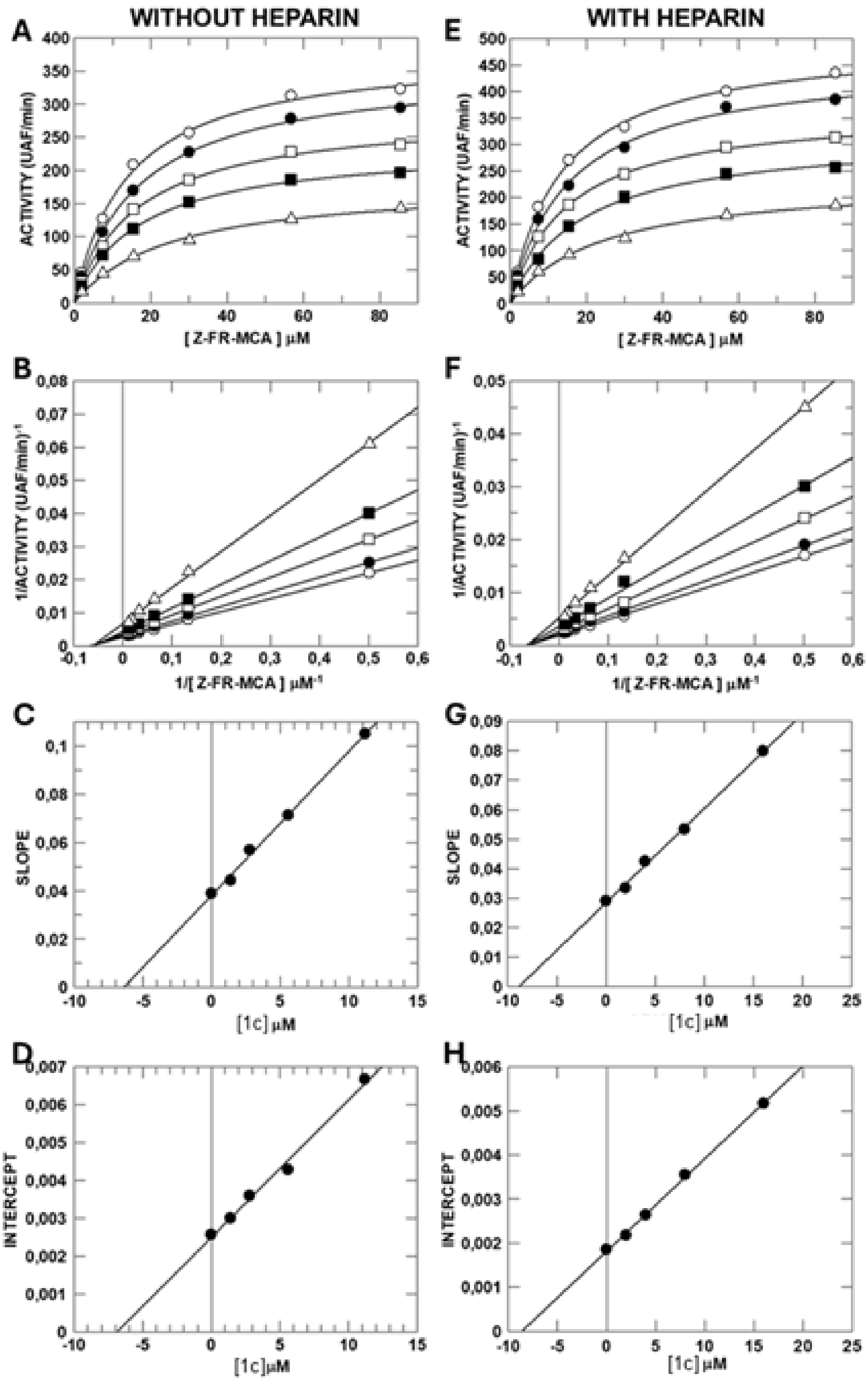

Figure S3: Mechanism of cathepsin B inhibition by **1c** in the absence and presence of 10 µM heparin. (A, E) Michaelis-Menten plots; (B, F) Double-reciprocal (Lineweaver-Burk) plots; (C, G) Slope replots; (D, H) Intercept replots. In A and B: ○→ control; ●→ 1.4 µM; □→ 2.8 µM; ■→ 5.6 µM; △→ 11.2 µM. In E and F: ○→ control; ●→ 2 µM; □→ 4 µM; ■→ 8 µM; △→ 16 µM.

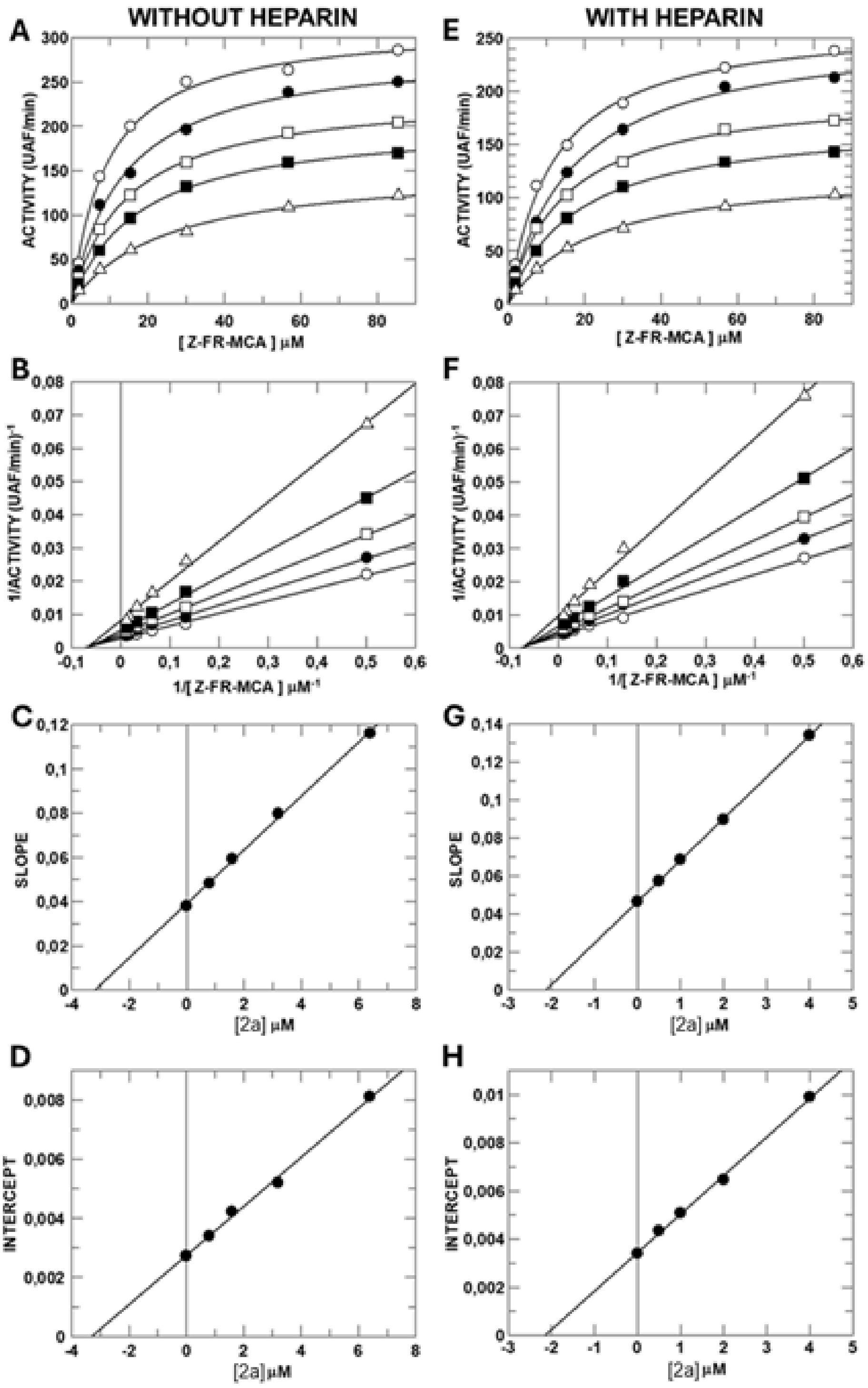

Figure S4: Mechanism of cathepsin B inhibition by compound 2a in the absence and presence of 10 μM heparin. (A, E) Michaelis-Menten plots; (B, F) Double-reciprocal (Lineweaver-Burk) plots; (C, G) Slope replots; (D, H) Intercept replots. In A and B: ○→ control; ●→ 0.8 μM; □→ 1.6 μM; ■→ 3.2 μM; △→ 6.4 μM. In E and F: ○→ control; ●→ 0.5 μM; □→ 1 μM; ■→ 2 μM; △→ 4 μM.

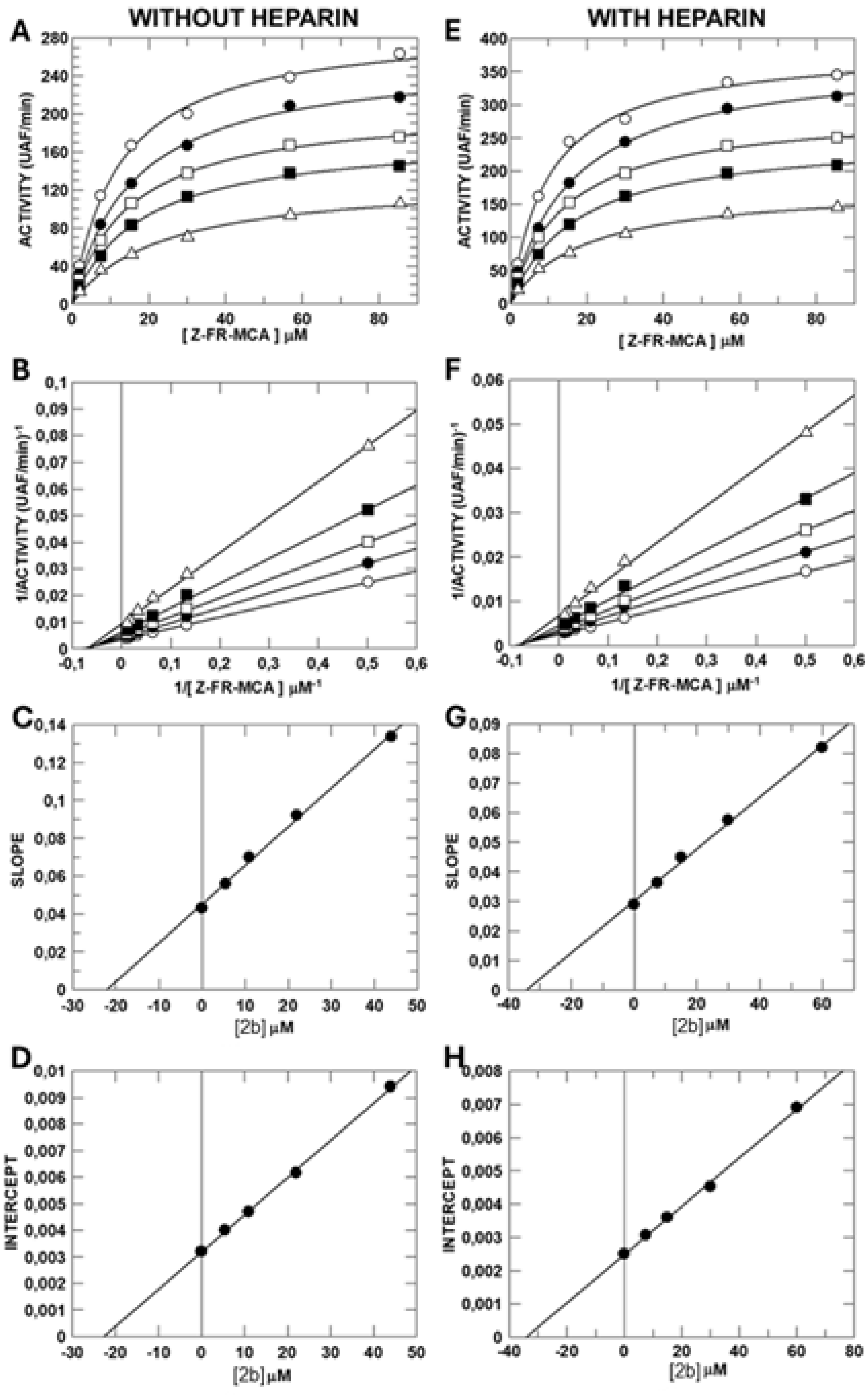

Figure S5: Mechanism of cathepsin B inhibition by 2b in the absence and presence of 10 µM heparin. (A, E) Michaelis-Menten plots; (B, F) Double-reciprocal (Lineweaver-Burk) plots; (C, G) Slope replots; (D, H) Intercept replots. In A and B: ○→ control; ●→ 5.5 µM; □→ 11 µM; ■→ 22 µM; △→ 44 µM. In E and F: ○→ control; ●→ 7.5 µM; □→ 15 µM; ■→ 30 µM; △→ 60 µM.

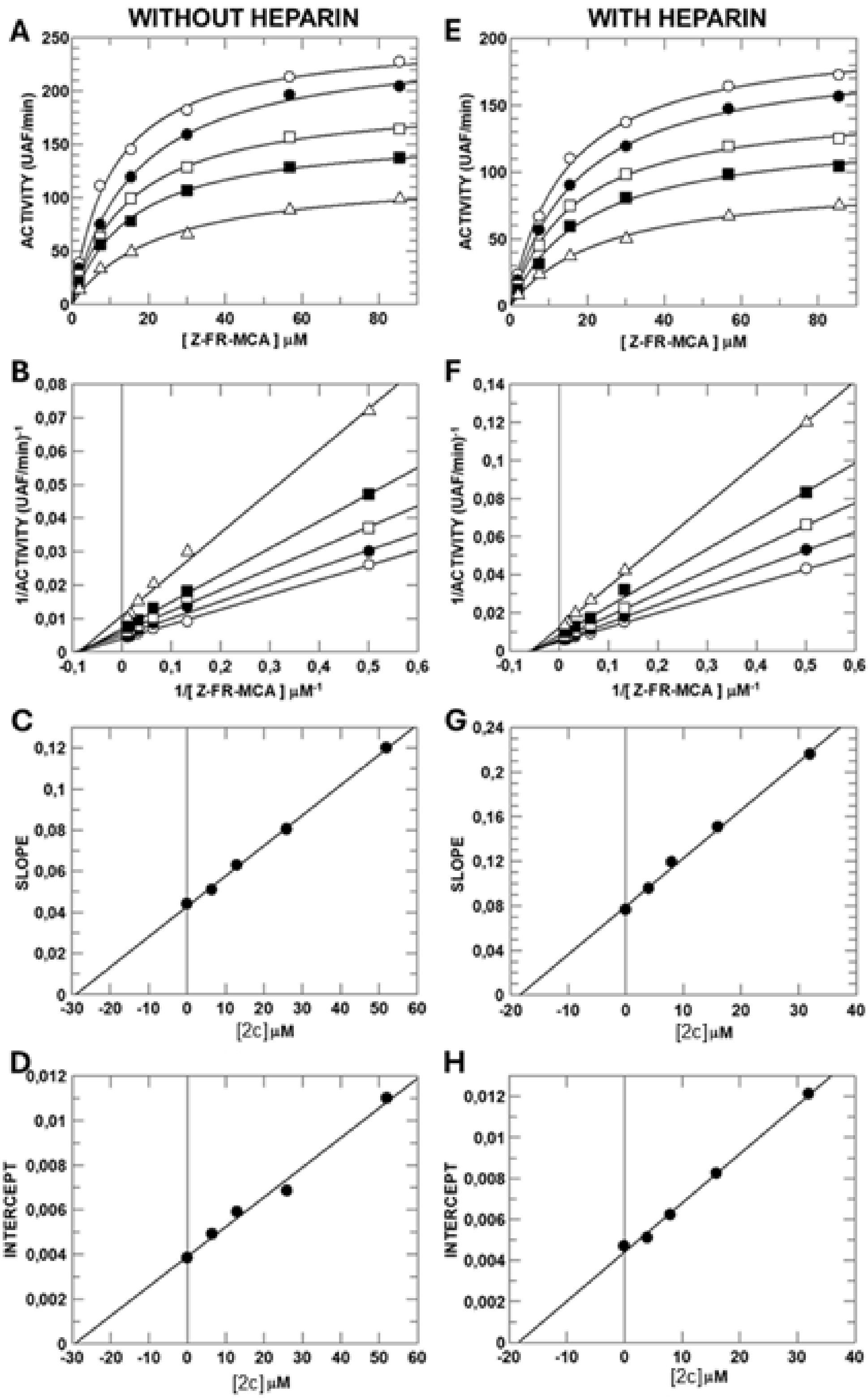

Figure S6: Mechanism of cathepsin B inhibition by **2c** in the absence and presence of 10 μM heparin. (A, E) Michaelis-Menten plots; (B, F) Double-reciprocal (Lineweaver-Burk) plots; (C, G) Slope replots; (D, H) Intercept replots. In A and B: ○→ control; ●→ 6.5 μM; □→ 13 μM; ■→ 26 μM; △→ 52 μM. In E and F: ○→ control; ●→ 4 μM; □→ 8 μM; ■→ 16 μM; △→ 32 μM.

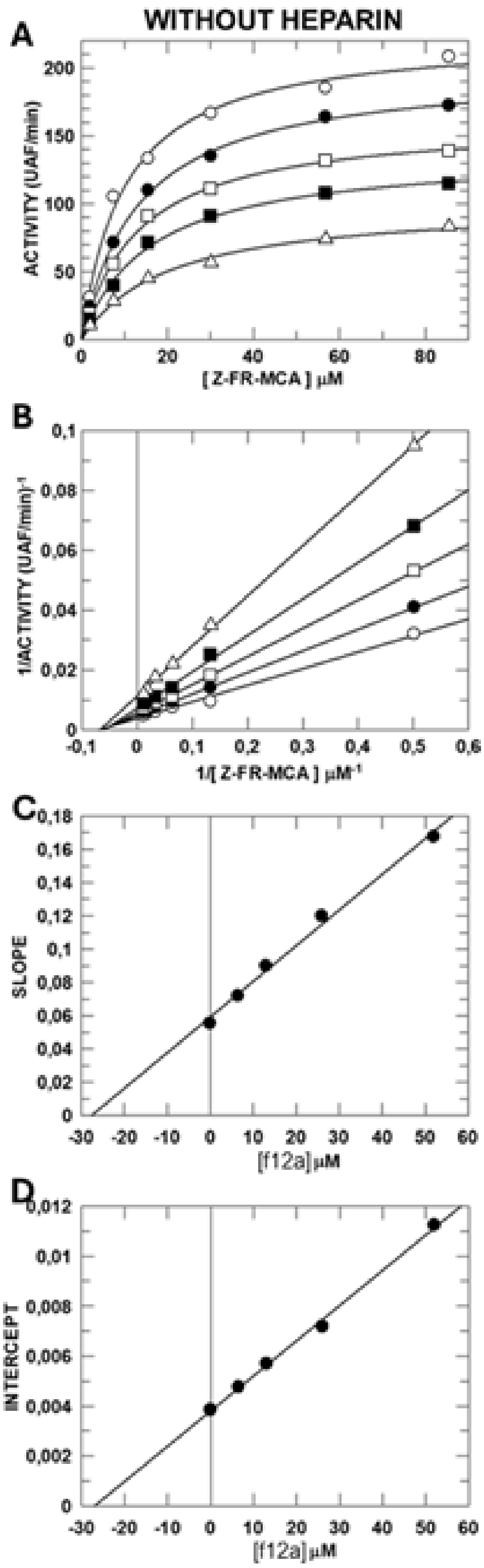

Figure S7: Mechanism of cathepsin B inhibition by **f12a** in the absence of heparin. (A) Michaelis-Menten plots; (B) Double-reciprocal (Lineweaver-Burk) plots; (C) Slope replots; (D) Intercept replots. In A and B: ○→ control; ●→ 6.5 µM; □→ 13 µM; ■→ 26 µM; △→ 52 µM.

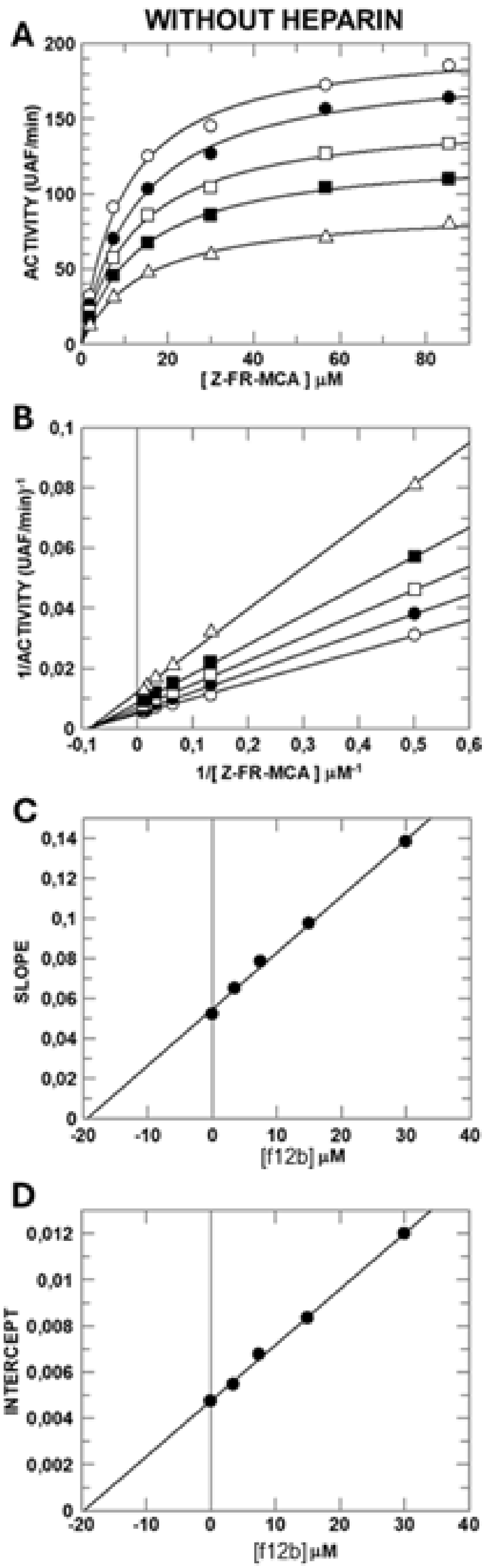

Figure S8: Mechanism of cathepsin B inhibition by **f12b** in the absence of heparin. (A) Michaelis-Menten plots; (B) Double-reciprocal (Lineweaver-Burk) plots; (C) Slope replots; (D) Intercept replots. In A and B: ○→ control; ●→ 3.5 µM; □→ 7.5 µM; ■→ 15 µM; △→ 30 µM.

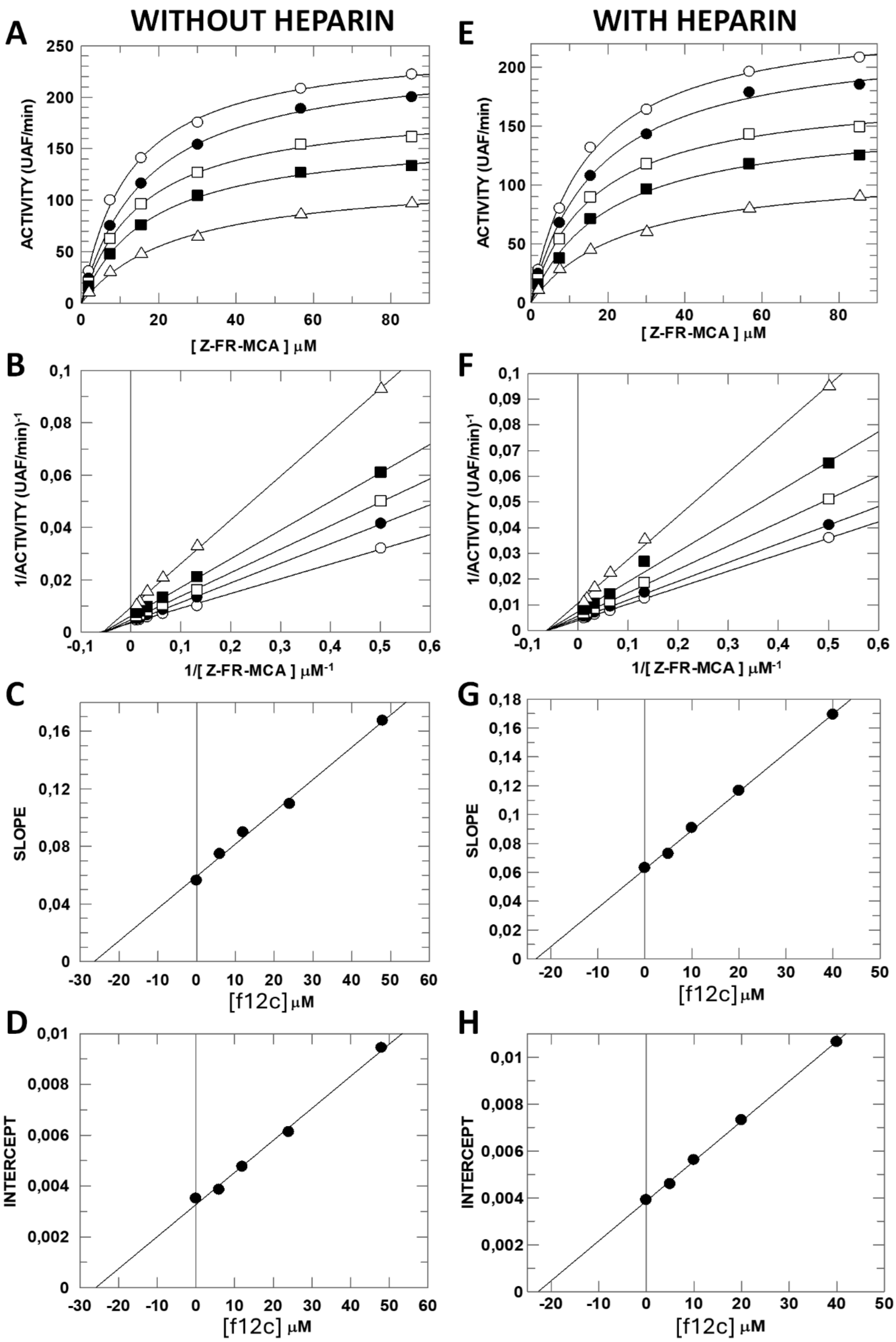

Figure S9: Mechanism of cathepsin B inhibition by **f12c** in the absence and presence of 10 μM heparin. (A, E) Michaelis-Menten plots; B, F) Double-reciprocal (Lineweaver-Burk) plots; C, G) Slope replots; D, H) Intercept replots. In A and B: ○→ control; ●→ 6 μM; □→ 12 μM; ■→ 24 μM; △→ 48 μM. In E and F: ○→ control; ●→ 5 μM; □→ 10 μM; ■→ 20 μM; △→ 40 μM.

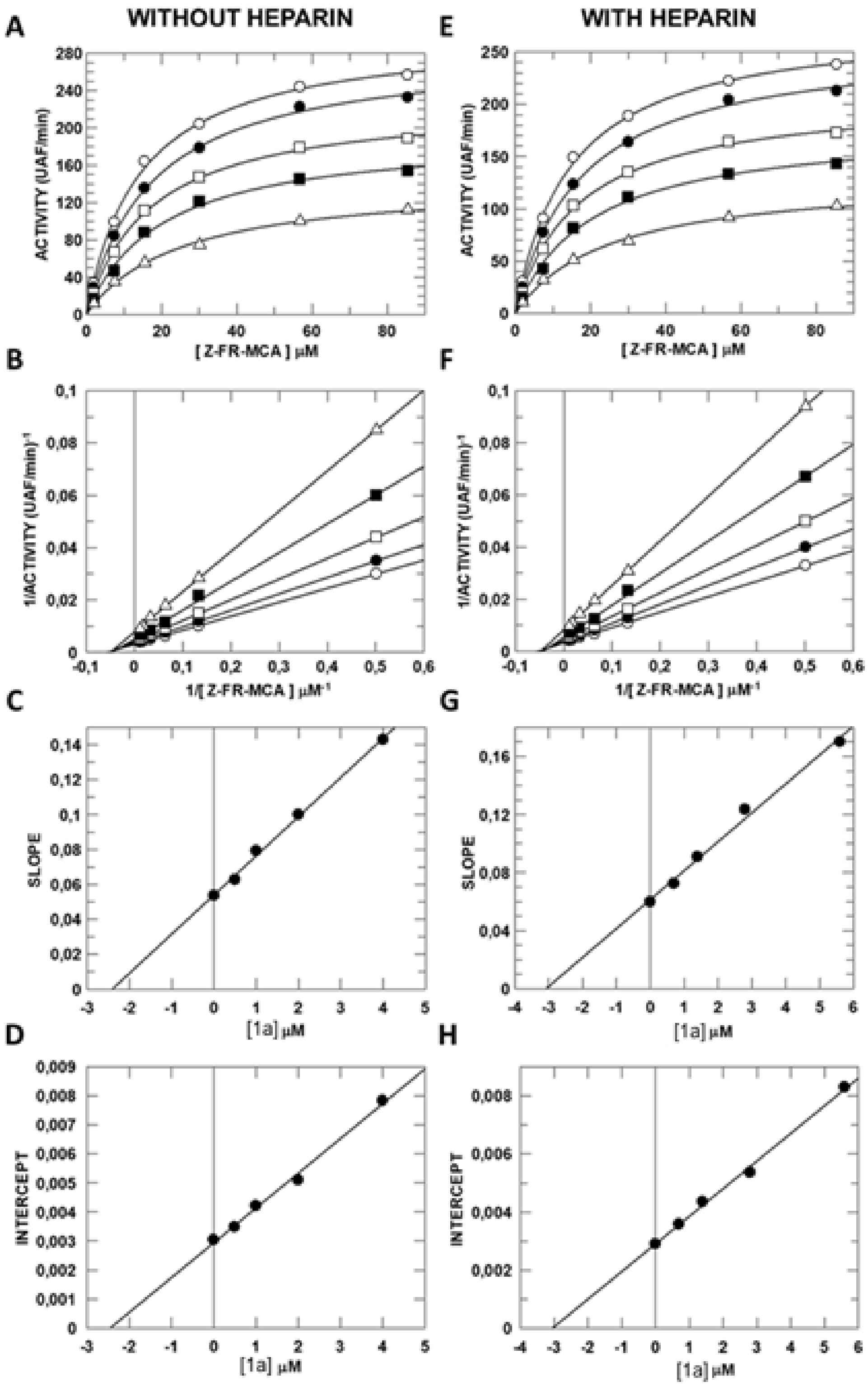

Figure S10: Mechanism of cathepsin L inhibition by **1a** in the absence and presence of 10 µM heparin. (A, E) Michaelis-Menten plots; (B, F) Double-reciprocal (Lineweaver-Burk) plots; (C, G) Slope replots; (D, H) Intercept replots. In A and B:○→ control; ●→ 0.5 µM; □→ 1 µM; ■→ 2 µM; △→ 4 µM. In E and F: ○→ control; ●→ 0.7 µM; □→ 1.4 µM; ■→ 2.8 µM; △→ 5.6 µM.

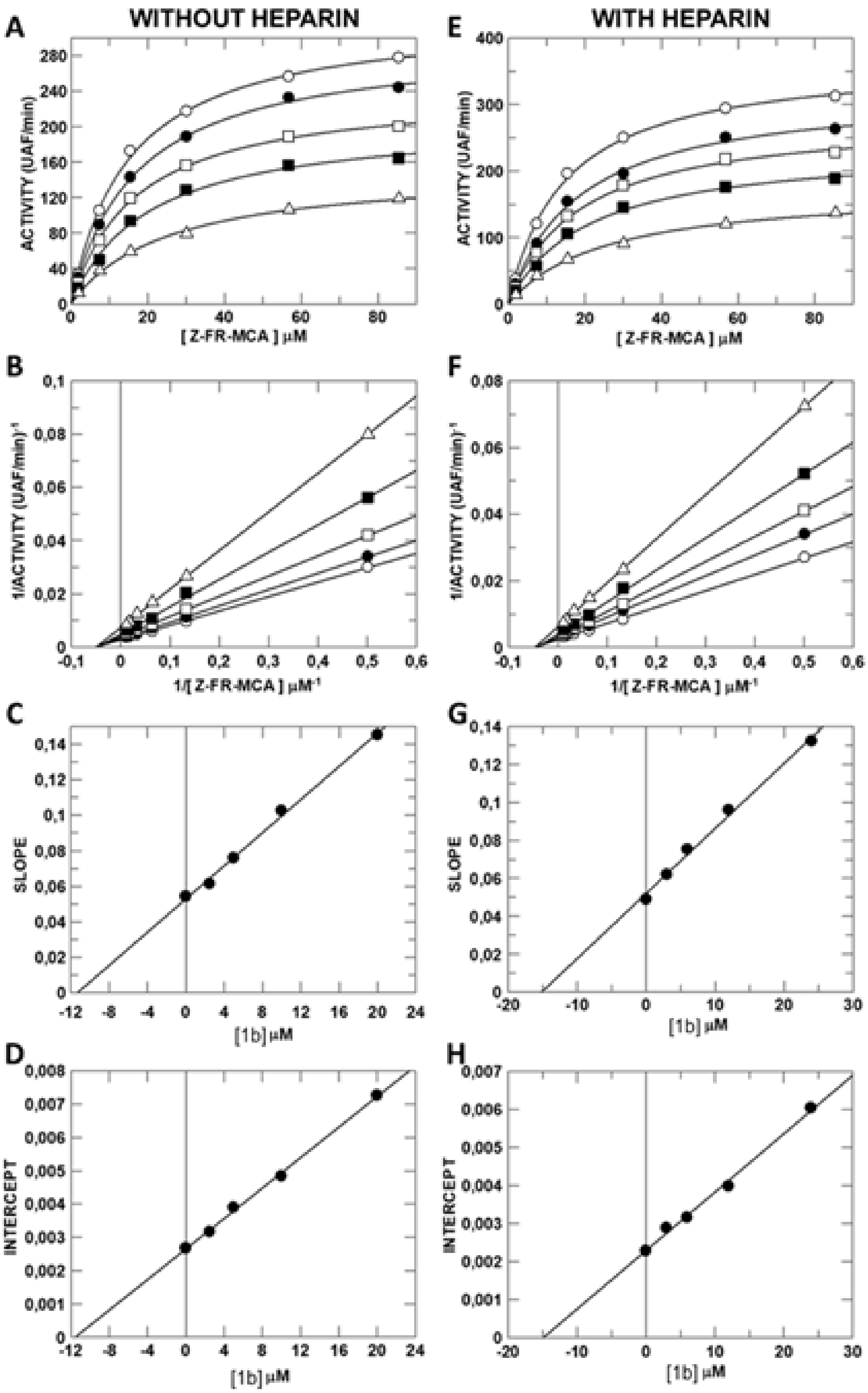

Figure S11: Mechanism of cathepsin L inhibition by **1b** in the absence and presence of 10 μM heparin. (A, E) Michaelis-Menten plots; (B, F) Double-reciprocal (Lineweaver-Burk) plots; (C, G) Slope replots; (D, H) Intercept replots. In A and B: ○→ control; ●→ 2.5 μM; □→ 5 μM; ■→ 10 μM; △→ 20 μM. In E and F: ○→ control; ●→ 3 μM; □→ 6 μM; ■→ 12 μM; △→ 24 μM.

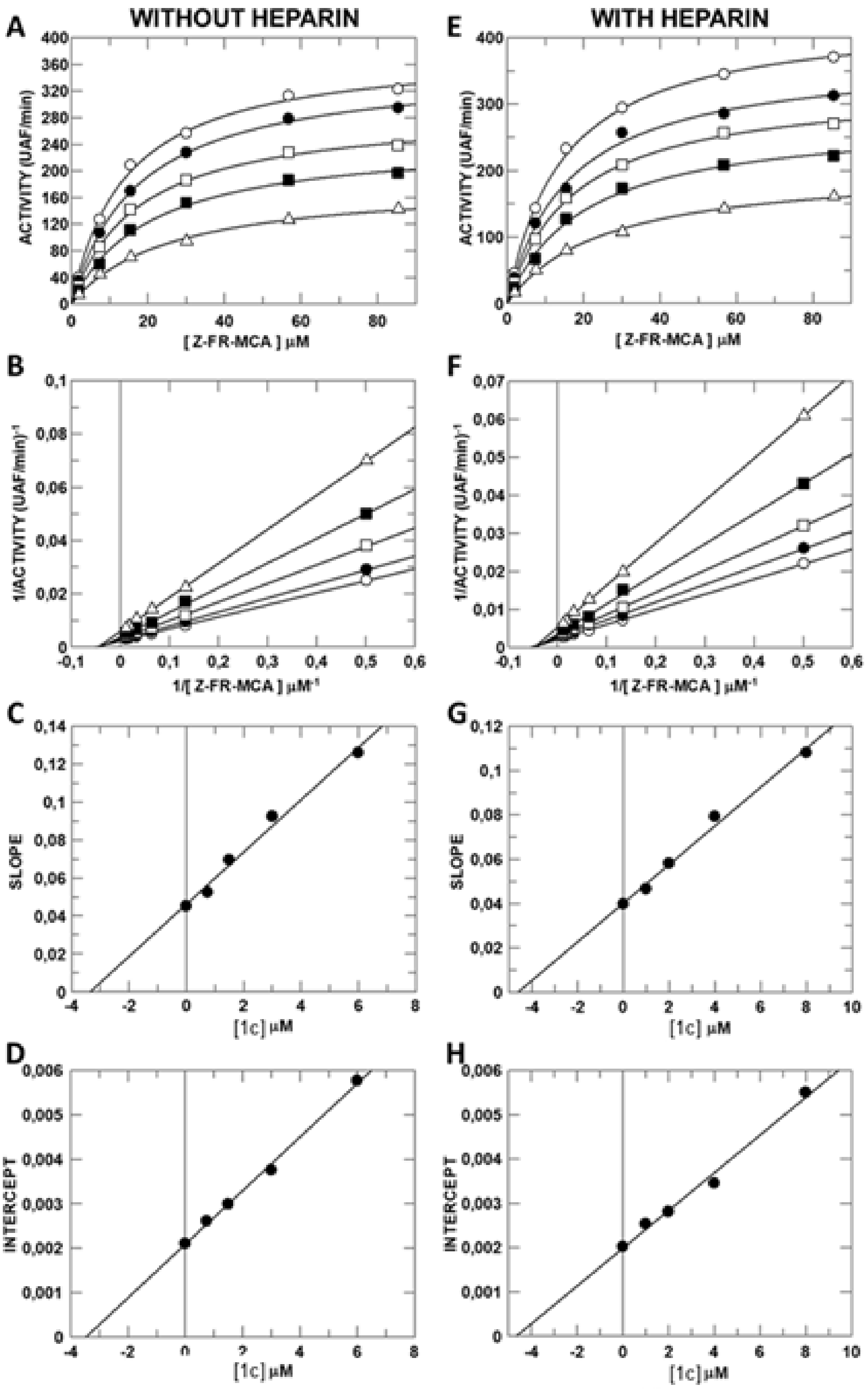

Figure S12: Mechanism of cathepsin L inhibition by **1c** in the absence and presence of 10 µM heparin. (A, E) Michaelis-Menten plots; (B, F) Double-reciprocal (Lineweaver-Burk) plots; (C, G) Slope replots; (D, H) Intercept replots. In A and B: ○→ control; ●→ 0.75 µM; □→ 1.5 µM; ■→ 3 µM; △→ 6 µM. In E and F: ○→ control; ●→ 1 µM; □→ 2 µM; ■→ 4 µM; △→ 8 µM.

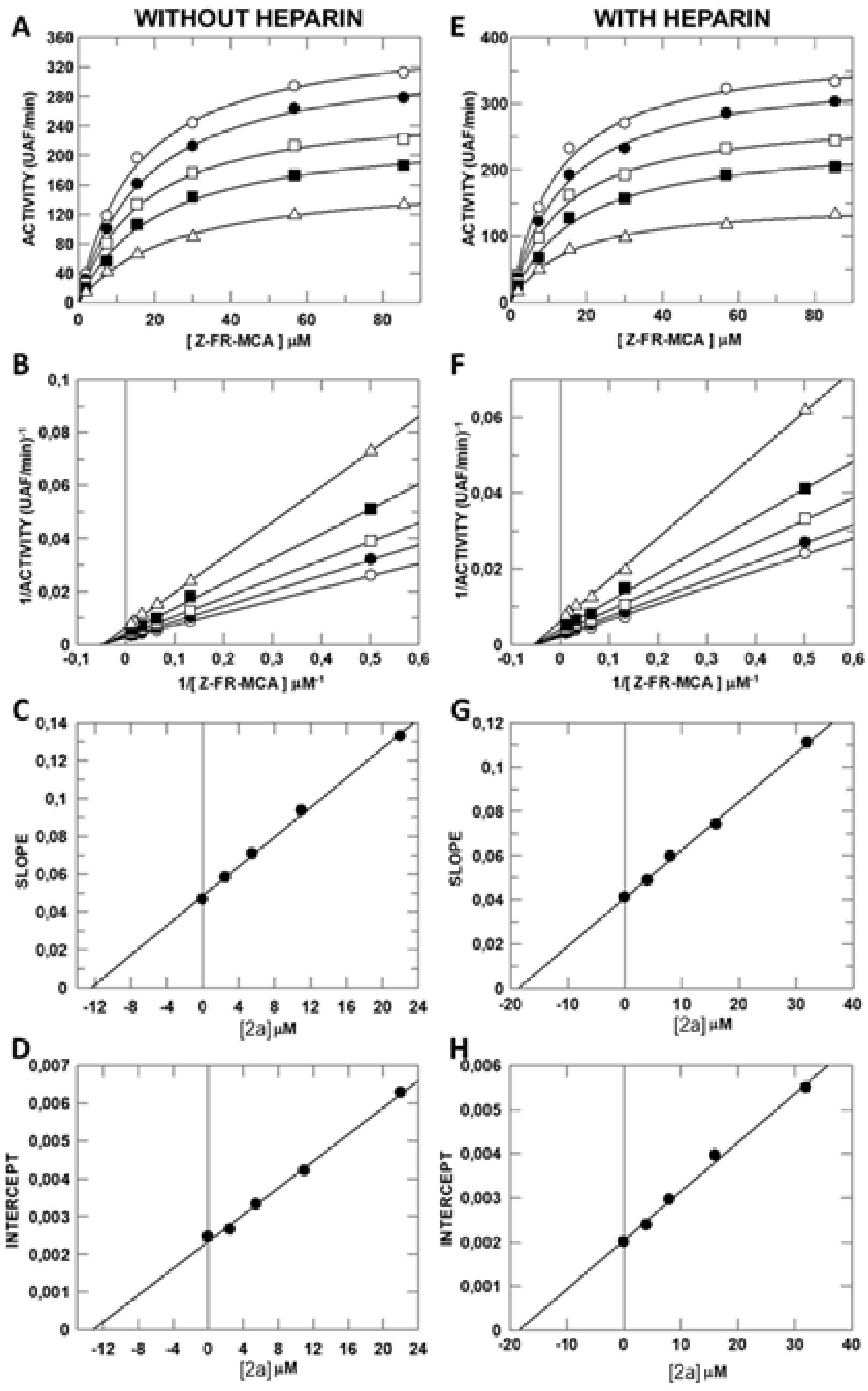

Figure S13: Mechanism of cathepsin L inhibition by **2a** in the absence and presence of 10 µM heparin. (A, E) Michaelis-Menten plots; (B, F) Double-reciprocal (Lineweaver-Burk) plots; (C, G) Slope replots; (D, H) Intercept replots. In A and B: ○→ control; ●→ 2.5 µM; □→ 5.5 µM; ■→ 11 µM; △→ 22 µM. In E and F: ○→ control; ●→ 1 µM; □→ 8 µM; ■→ 16 µM; △→ 32 µM.

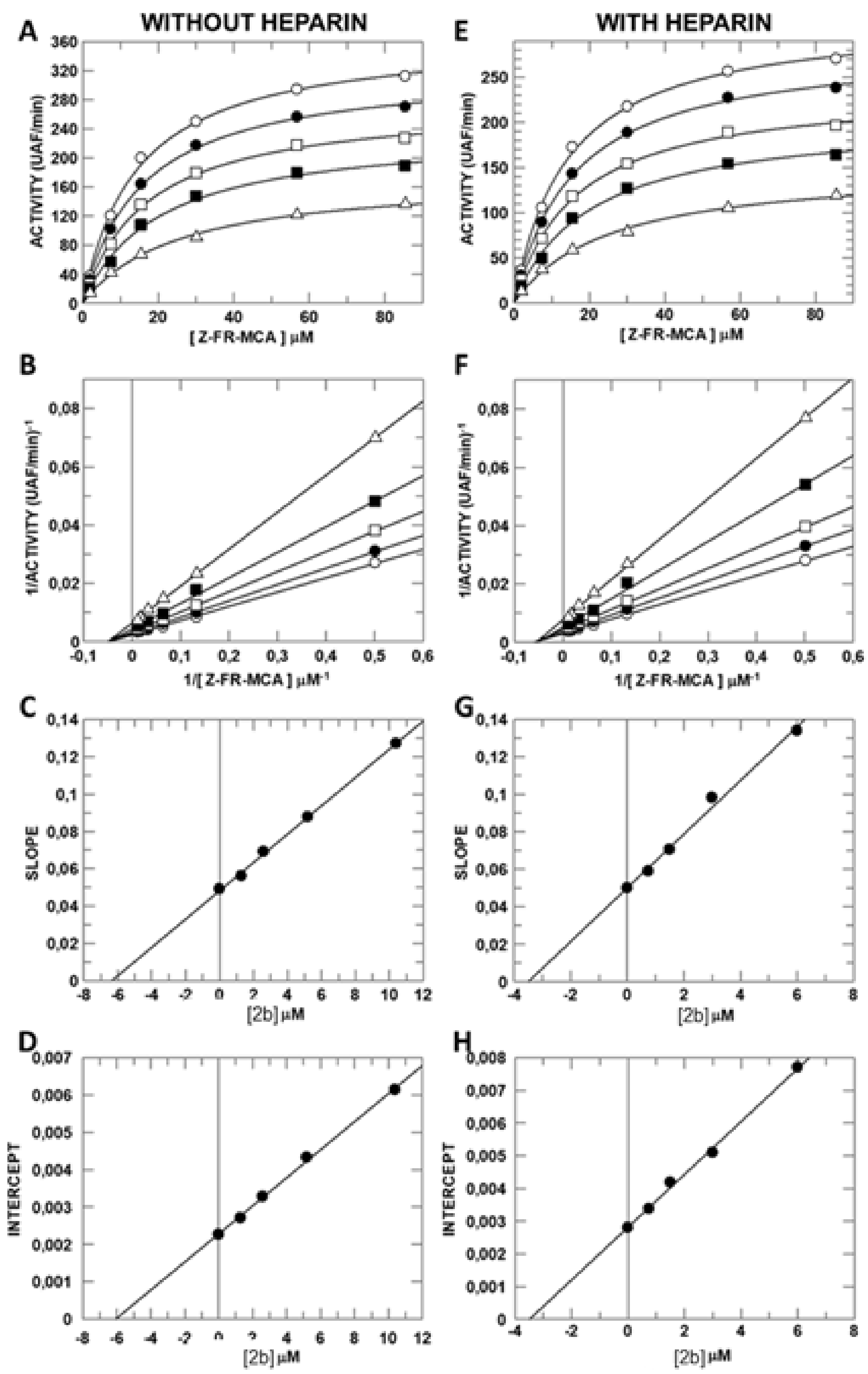

Figure S14: Mechanism of cathepsin L inhibition by **2b** in the absence and presence of 10 μM heparin. (A, E) Michaelis-Menten plots; (B, F) Double-reciprocal (Lineweaver-Burk) plots; (C, G) Slope replots; (D, H) Intercept replots. In A and B: ○→ control; ●→ 1.3 μM; □→ 2.6 μM; ■→ 5.2 μM; △→ 10.4 μM. In E and F: ○→ control; ●→ 0.75 μM; □→ 1.5 μM; ■→ 3 μM; △→ 6 μM.

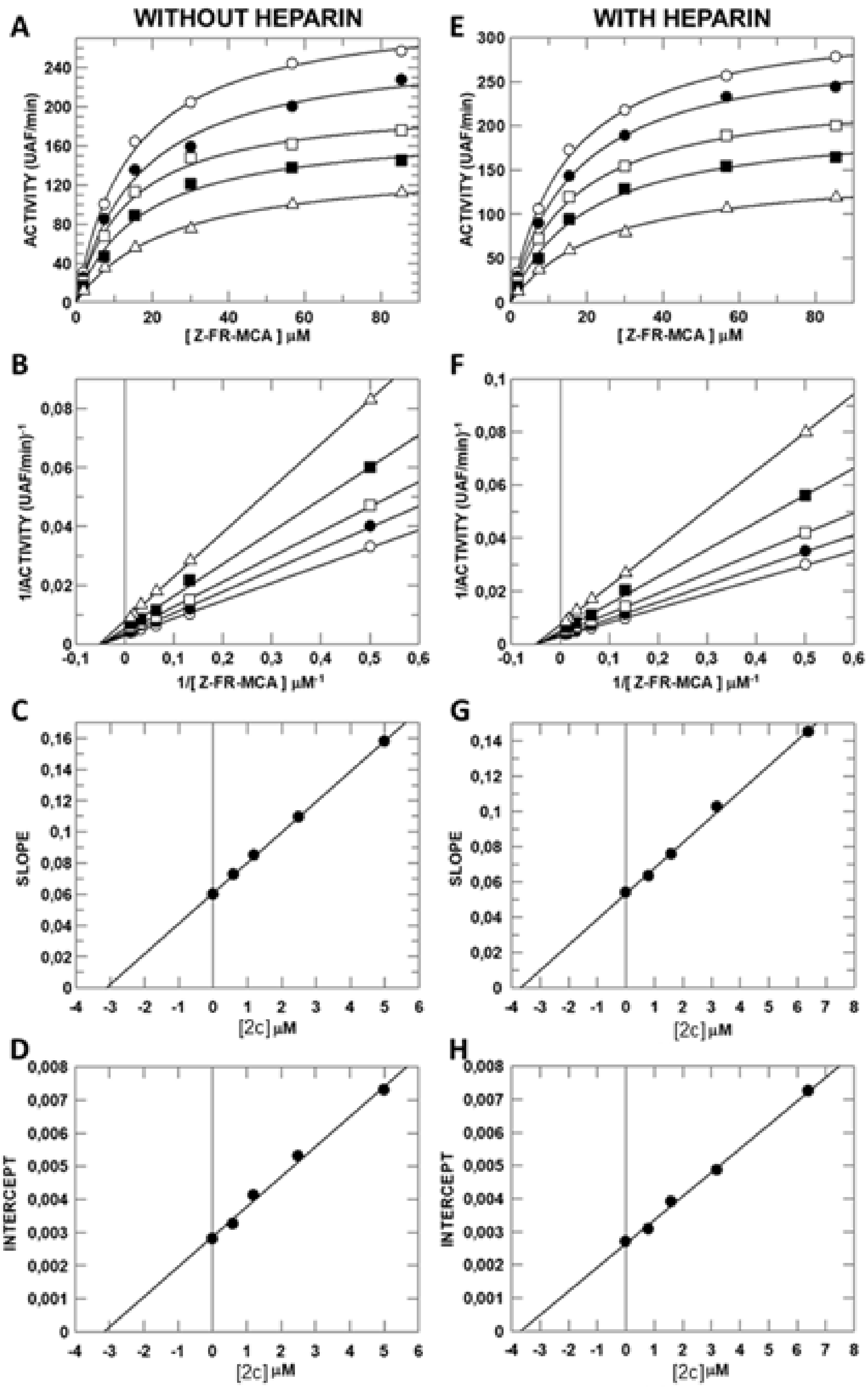

Figure S15: Mechanism of cathepsin L inhibition by 2c in the absence and presence of 10 µM heparin. (A, E) Michaelis-Menten plots; (B, F) Double-reciprocal (Lineweaver-Burk) plots; (C, G) Slope replots; (D, H) Intercept replots. In A and B: ○→ control; ●→ 0.6 µM; □→ 1.2 µM; ■→ 2.5 µM; △→ 5 µM. In E and F: ○→ control; ●→ 0.8 µM; □→ 1.6 µM; ■→ 3.2 µM; △→ 6.4 µM.

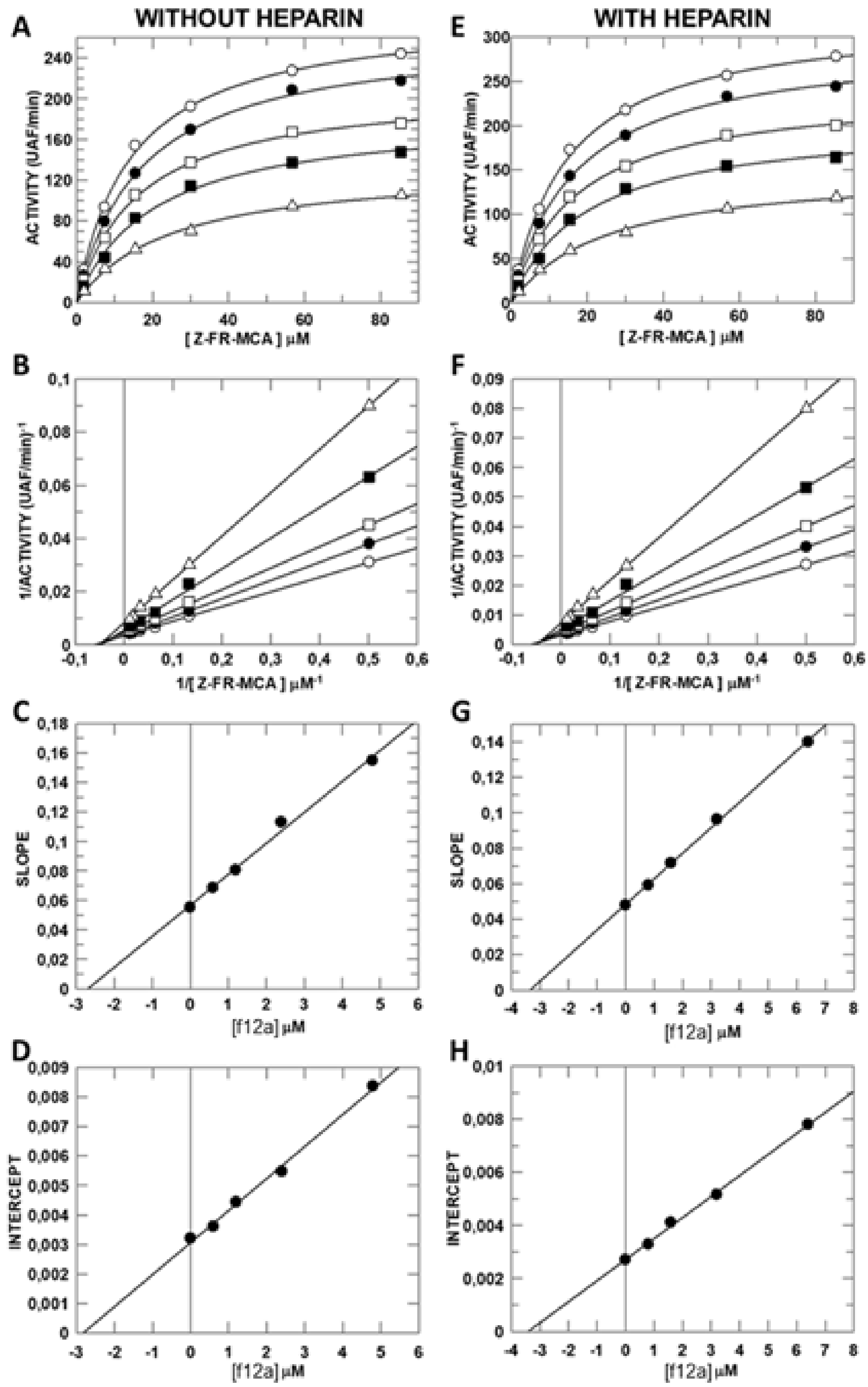

Figure S16: Mechanism of cathepsin L inhibition by f12a in the absence and presence of 10 µM heparin. (A, E) Michaelis-Menten plots; (B, F) Double-reciprocal (Lineweaver-Burk) plots; (C, G) Slope replots; (D, H) Intercept replots. In A and B: ○→ control; ●→ 0.6 µM; □→ 1.2 µM; ■→ 2.4 µM; △→ 4.8 µM. In E and F: ○→ control; ●→ 0.8 µM; □→ 1.6 µM; ■→ 3.2 µM; △→ 6.4 µM.

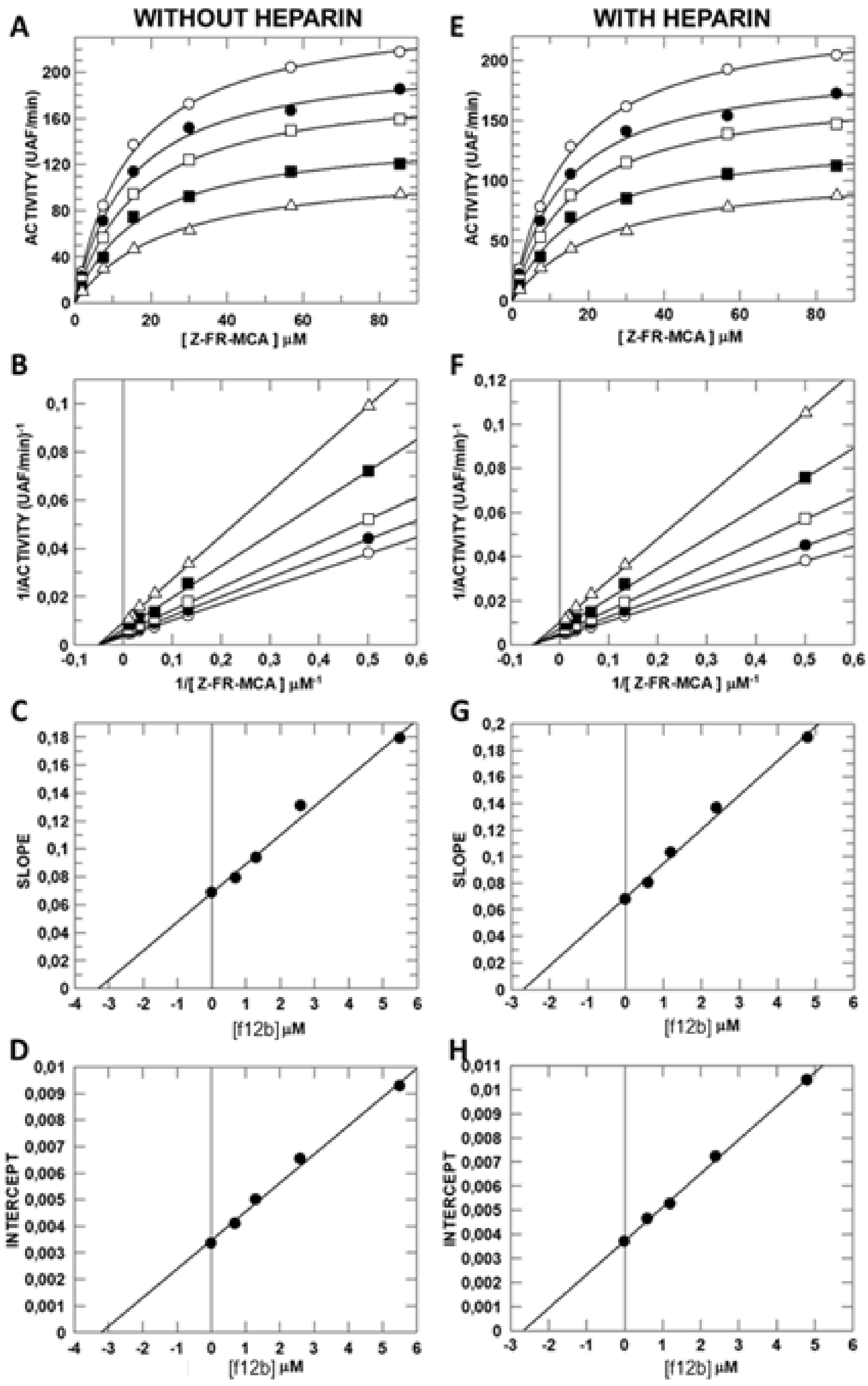

Figure S17: Mechanism of cathepsin L inhibition by **f12b** in the absence and presence of 10 μM heparin. (A, E) Michaelis-Menten plots; (B, F) Double-reciprocal (Lineweaver-Burk) plots; (C, G) Slope replots; (D, H) Intercept replots. In A and B: ○→ control; ●→ 0.7 μM; □→ 1.3 μM; ■→ 2.6 μM; △→ 5.5 μM. In E and F: ○→ control; ●→ 0.8 μM; □→ 1.6 μM; ■→ 3.2 μM; △→ 6.4 μM.

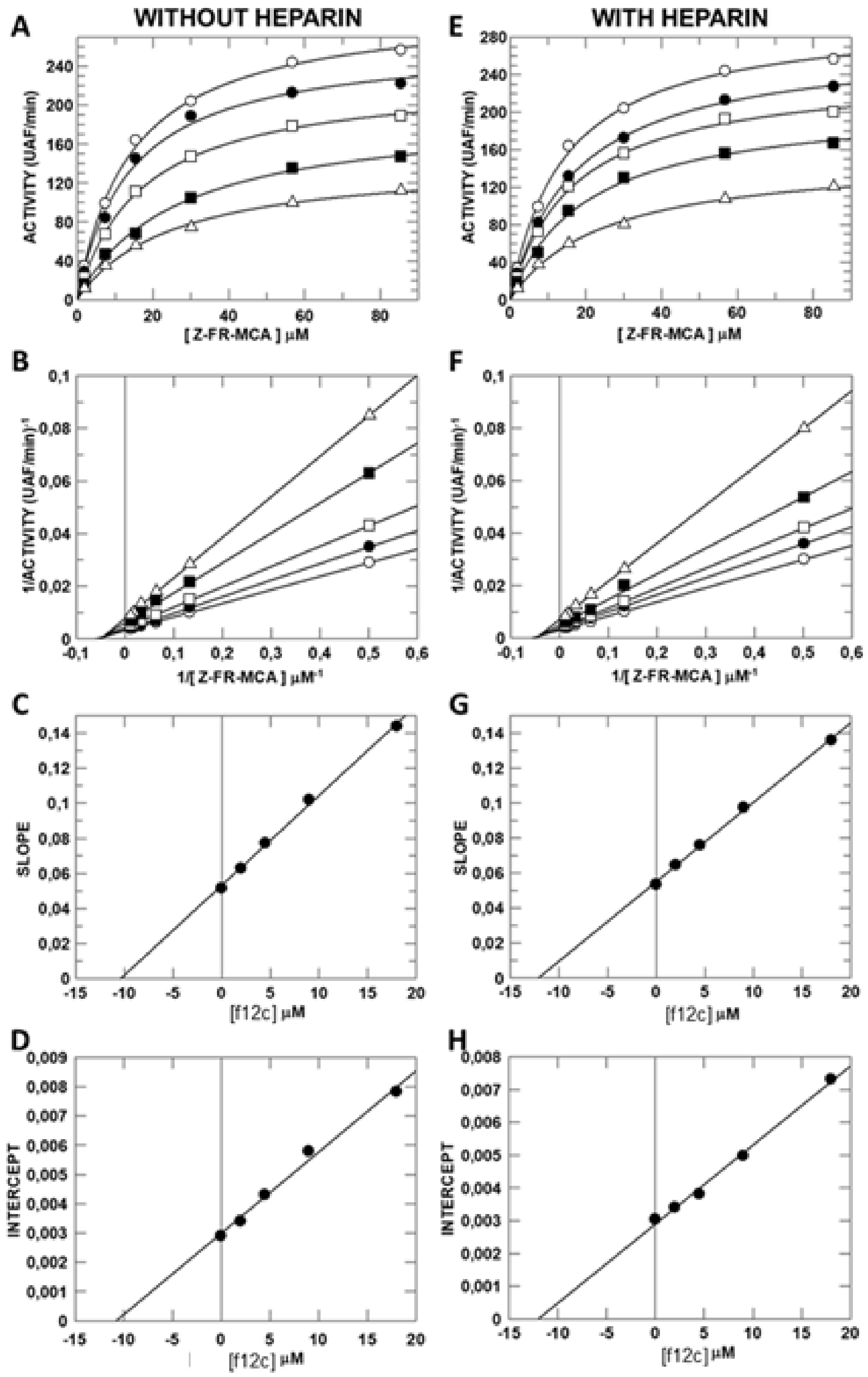

Figure S18: Mechanism of cathepsin L inhibition by **f12c** in the absence and presence of 10 µM heparin. (A, E) Michaelis-Menten plots; (B, F) Double-reciprocal (Lineweaver-Burk) plots; (C, G) Slope replots; (D, H) Intercept replots. In A and B: ○→ control; ●→ 2 µM; □→ 4.5 µM; ■→ 9 µM; △→ 18 µM. In E and F: ○→ control; ●→ 2 µM; □→ 4.5 µM; ■→ 9 µM; △→ 18 µM.

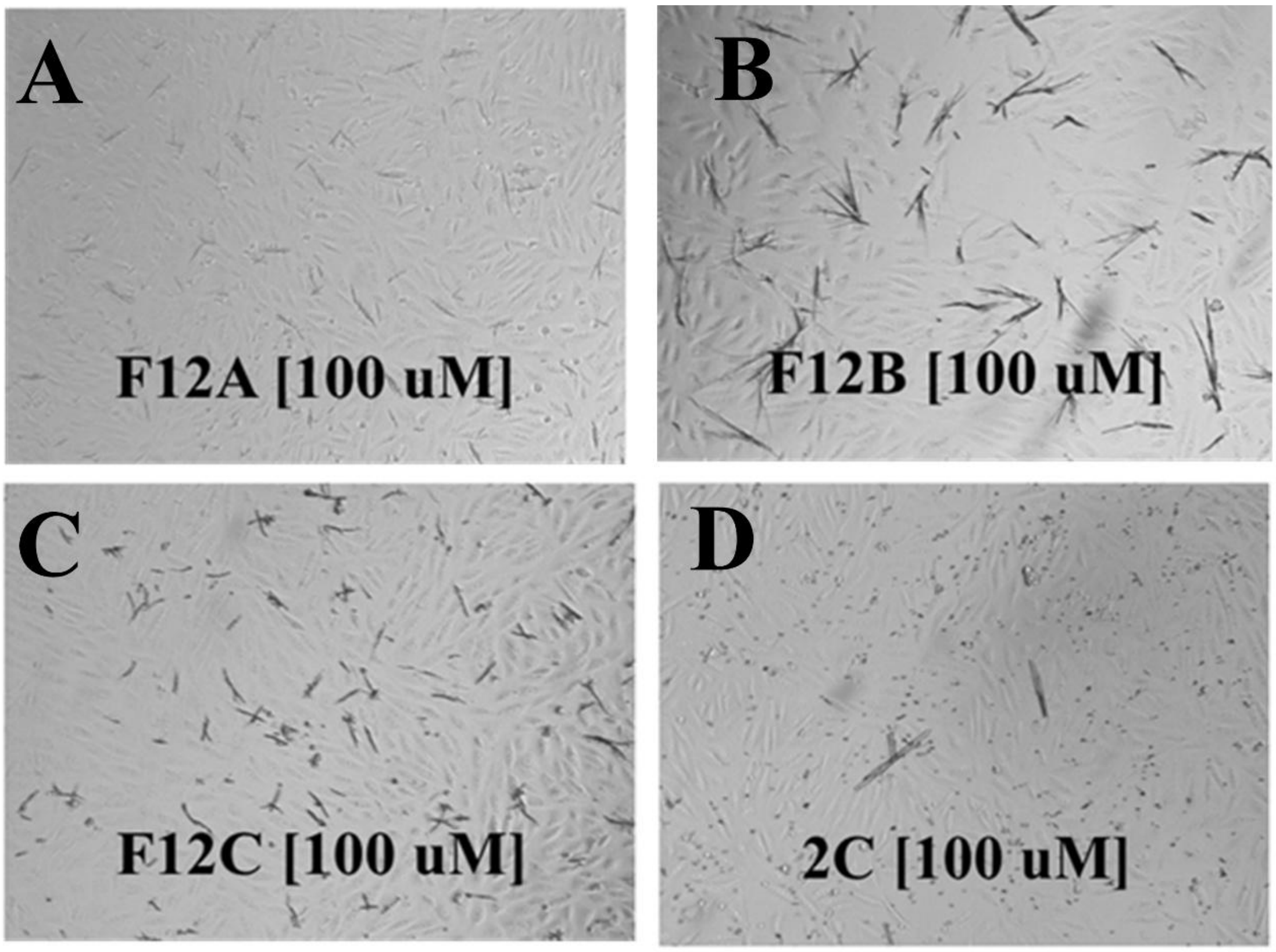

Figure S19: Microscopic analysis of the wells in the solubility assay for compounds **f12a** (**A**), **f12b** (**B**), **f12c** (**C**), and **2c** (**D**). Vero-CCL81 cells (3×10^4 cells/well) were seeded in 96-well plates and incubated in Minimum Essential Medium (MEM) supplemented with 1% FBS during the assay.

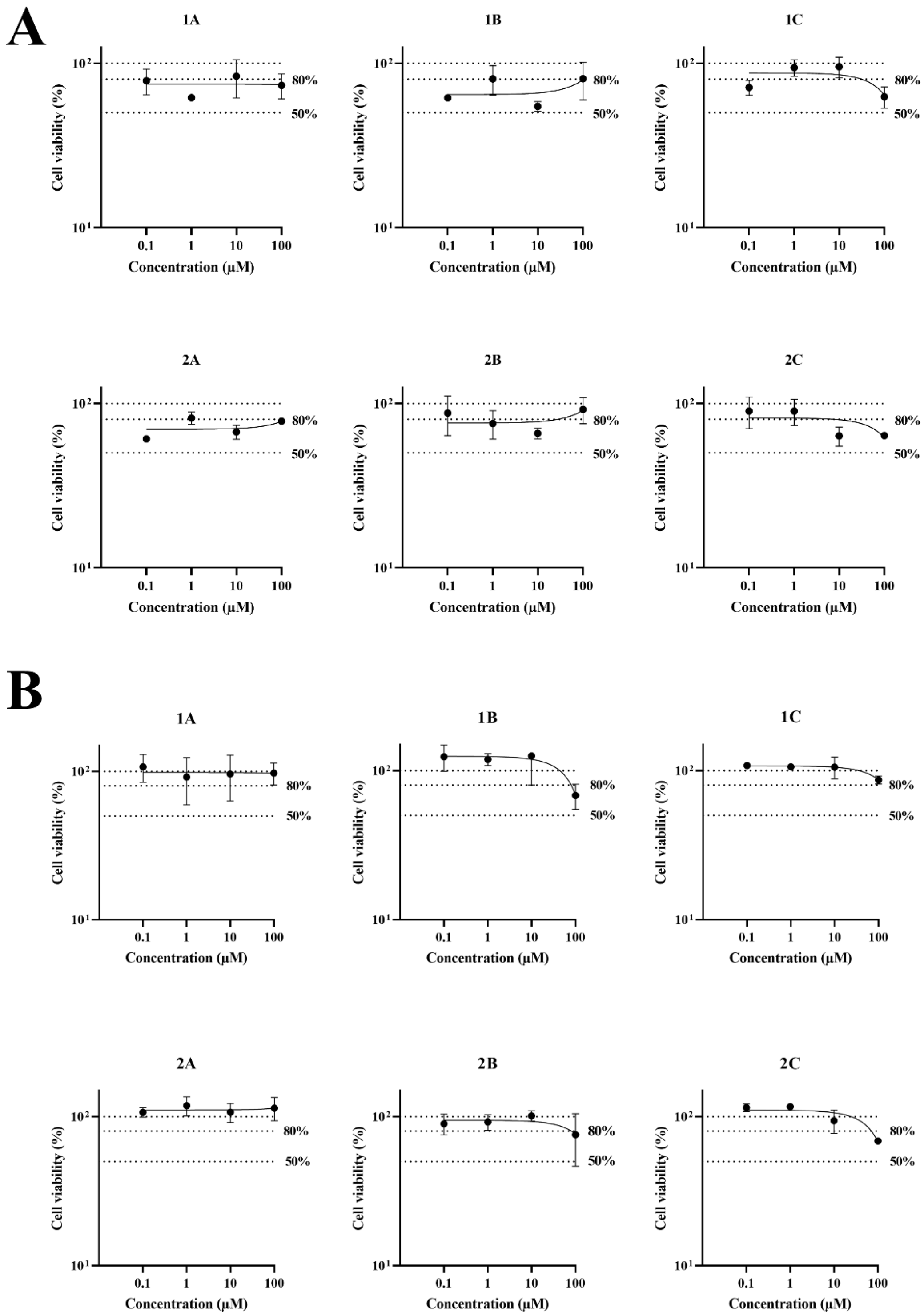

Figure S20: Cytotoxicity assays for compounds **1a**–**2c** at concentrations ranging from 0.01 to 100 µM against Vero CCL-81 (**A**) and L929 (**B**) cells. All conditions were performed in internal triplicate and reproduced in two independent experiments (n = 6).

Spectroscopic data

(*E*)-1-(4-chlorophenyl)-3-(4-methoxyphenyl)prop-2-en-1-one (1a): pale yellow crystal. Yield 67.11%. Mp 118–120 °C. $R_f = 0.535$. ^{1}H-NMR ($CDCl_3$, 300 MHz) δ ppm 7.96 (2H, d, J = 8.6, ArH), 7.81 (1H, d, J = 15.6 Hz, C=CH), 7.61 (2H, d, J = 8.7, ArH), 7.47 (2H, d, J = 8.6, ArH), 7.38 (1H, d, J = 15.6 Hz, C=CH), 6.95 (2H, d, J = 8.7, ArH), 3.85 (3H, s, –OCH_3); ^{13}C-NMR ($CDCl_3$, 75 MHz) δ ppm 189.29, 161.97, 145.32, 139.05, 136.92, 130.45, 129.94, 128.98, 127.54, 119.27, 114.58, 55.54. MALDI-TOF MS (negative mode) m/z: 272.253 ($[M]^{-\bullet}$).

(*E*)-3-(benzo[d][1,3]dioxol-5-yl)-1-(4-chlorophenyl)prop-2-en-1-one (1b): pale yellow crystal. Yield 86.19%. Mp 126–130 °C. $R_f = 0.56$. ^{1}H-NMR ($CDCl_3$, 300 MHz) δ ppm 7.96 (2H, d, J = 8.5, ArH), 7.76 (1H, d, J = 15.6 Hz, C=CH), 7.48 (2H, d, J = 8.5, ArH), 7.33 (1H, d, J = 15.6 Hz, C=CH), 7.15 (1H, d, J = 1.6 Hz, ArH), 7.13 (1H, dd, J = 8.1, ArH), 6.85 (1H, dd, J = 8.1, ArH), 6.03 (2H, s, –OCH_2O); ^{13}C-NMR ($CDCl_3$, 75 MHz) δ ppm 188.69, 149.80, 148.16, 144.88, 138.74, 136.38, 129.52, 128.87, 125.13, 119.16, 108.41, 106.35, 101.40. MALDI-TOF MS (negative mode) m/z: 286.755 ($[M]^{-\bullet}$).

(*E*)-1-(4-chlorophenyl)-3-(4-(dimethylamino)phenyl)prop-2-en-1-one (1c): yellow amorphous powder. Yield 73.26%. Mp 138–140 °C. $R_f = 0.40$. ^{1}H-NMR ($CDCl_3$, 300 MHz) δ ppm 7.96 (2H, d, J = 8.6, ArH), 7.82 (1H, d, J = 15.5 Hz, C=CH), 7.55 (2H, d, J = 8.9, ArH), 7.46 (2H, d, J = 8.6, ArH), 7.30 (1H, d, J = 15.5 Hz, C=CH), 6.70 (2H, d, J = 8.9, ArH), 3,04 (6H, s, –$N(CH_3)_2$); ^{13}C-NMR ($CDCl_3$, 75 MHz) δ ppm 189.94, 151.86, 146.08, 138.12, 137.10, 130.25, 129.43, 128.42, 122.13, 115.90, 111.50, 39.81. MALDI-TOF MS (negative mode) m/z: 284.850 ($[M - H]^-$).

(*E*)-1-(2-hydroxyphenyl)-3-(4-methoxyphenyl)prop-2-en-1-one (2a): yellow amorphous powder. Yield 76.53%. Mp 90–94 °C. $R_f = 0.60$. ^{1}H-NMR ($CDCl_3$, 300 MHz) δ ppm 12.96 (1H, s, OH), 7.92 (1H, dd, J = 2.3, 8.6 Hz, ArH), 7.91(1H, d, J = 15.5 Hz, C=CH), 7.62 (2H, d, J = 8.6 Hz, ArH), 7.55 (1H, d, J = 15.5 Hz, C=CH), 7.50–7.45 (1H, m, ArH), 7.02 (1H, d, J = 8.4 Hz, ArH), 6.95 (2H, d, J = 8.4 Hz, ArH), 7.00–6.90 (1H, m, ArH), 3,86 (3H, s, –OCH_3); ^{13}C-NMR ($CDCl_3$, 75 MHz) δ ppm 193.35, 163.23, 161.72, 145.04, 135.82, 130.24, 129.22, 127.02, 119.80, 118.44, 118.25, 117.25, 114.20, 55.12. MALDI-TOF MS (negative mode) m/z: 254.807 ($[M]^{-\bullet}$).

(*E*)-3-(benzo[d][1,3]dioxol-5-yl)-1-(2-hydroxyphenyl)prop-2-en-1-one (2b): yellow amorphous powder. Yield 71.43%. Mp 138–140 °C. $R_f = 0.64$. ^{1}H-NMR ($CDCl_3$, 300 MHz) δ ppm 12.89 (1H, s, OH), 7.90 (1H, 1.7, 8.1 Hz, ArH), 7.86 (1H, d, J = 15.5 Hz, ArH), 7.50 (1H, ddd, J = 1.7, 7.9, 8.1 Hz, ArH), 7.50 (1H, d, J = 15.5 Hz, ArH), 7.17 (1H, d, J = 1.7 Hz, ArH), 7.15 (1H, dd, J = 1.7, 8.1 Hz, ArH), 7.03 (1H, d, J = 8.1 Hz, ArH), 6.92 (1H, t, J = 7.2, 7.5, Hz, ArH), 6.86 (1H, d, J = 7.9 Hz, ArH), 6.03 (2H, s, –OCH_2O); ^{13}C-NMR ($CDCl_3$, 75 MHz) δ ppm 193.23, 163.25, 150.0, 148.20, 145.02, 135.91, 129.21, 128.77, 125.44, 119.76, 118.48, 118.29, 117.69, 108.44, 106.44, 101.45. MALDI-TOF MS (negative mode) m/z: 268.805 ($[M]^{-\bullet}$).

(*E*)-3-(4-(dimethylamino)phenyl)-1-(2-hydroxyphenyl)prop-2-en-1-one (2c): purple crystals. Yield 82.34%. Mp 178–181 °C. $R_f = 0.71$. ^{1}H-NMR ($CDCl_3$, 300 MHz) δ ppm 13.23 (1H, s, OH), 7.93 (1H, d, J = 15.4, C=CH), 7.90 (1H, d, J = 5.2 Hz, ArH), 7.57 (2H,

d, J = 8.9 Hz, ArH), 7.48 (1H, d, J = 5.2 Hz, ArH), 7.46 (1H, d, J = 15.4, C=CH), 7.01 (1H, d, J = 7.9 Hz, ArH), 6.94 (1H, t, J = 7.7 Hz, ArH), 6.69 (2H, d, J = 8.9 Hz, ArH), 3.04 (6H, s, –$N(CH_3)_2$); ^{13}C-NMR ($CDCl_3$, 75 MHz) δ ppm 193.18, 163.16, 152.02, 146.25, 135.32, 130.55, 129.06, 121.97, 120.08, 118.26, 118.13, 113.90, 111.48, 39.76. MALDI-TOF MS (negative mode) m/z: 267.885 ($[M]^{-\bullet}$).

3-hydroxy-2-(4-methoxyphenyl)-4H-chromen-4-one (f12a): yellow amorphous powder. Yield 71.34%. Mp 233–235 °C. R_f = 0.39. ^{1}H-NMR (DMSO-*d6*, 500 MHz) δ ppm 9.24 (1H, s, OH), 8.20 (2H, dt, J = 8.9, 3.0, 2.1 Hz, ArH), 8.12 (1H, dd, J = 6.3, 1.5 Hz, ArH), 7.77 (1H, dt, J = 7.8, 1.0 Hz, ArH), 7.72 (1H, dd, J = 8.6, 1.0 Hz, ArH), 7.45 (1H, dt, J = 7.8, 1.0 Hz, ArH), 7.12 (2H, dt, J = 8.9, 3.0, 2.1 Hz, ArH), 3.85 (3H, s, –OCH_3); ^{13}C-NMR (DMSO-*d6*, 125 MHz) δ ppm 172.48, 160.37, 154.35, 145.52, 137.95, 133.25, 129.24, 124.57, 124.26, 123.48, 121.23, 118.09, 113.91, 55.21. MALDI-TOF MS (negative mode) m/z: 268.821 ($[M]^{-\bullet}$).

2-(benzo[d][1,3]dioxol-5-yl)-3-hydroxy-4H-chromen-4-one (f12b): yellow amorphous powder. Yield 69.87%. Mp 213–215 °C. R_f = 0.39. ^{1}H-NMR (DMSO-*d6*, 300 MHz) δ ppm 9.52 (1H, s, OH), 8.10 (1H, d, J = 8.1 Hz, ArH), 7.84 (1H, dd, J = 8.5, 1.6 Hz, ArH), 7.78–7.73 (3H, m, ArH), 7.47 (1H, td, J = 8.1, 6.4, 1.7 Hz, ArH), 7.11 (1H, d, J = 8.3 Hz, ArH), 6.12 (2H, s, –OCH_2O); ^{13}C-NMR (DMSO-*d6*, 75 MHz) δ ppm 172.68, 154.40, 148.54, 147.52, 145.10, 138.33, 133.54, 125.05, 124.69, 122.76, 121.25, 118.36, 108.43, 107.52, 101.67. MALDI-TOF MS (negative mode) m/z: 282.805 ($[M]^{-\bullet}$).

2-(4-(dimethylamino)phenyl)-3-hydroxy-4H-chromen-4-one (f12c): Orange crystals. Yield 79.40%. Mp 182–183 °C. R_f = 0.39. ^{1}H-NMR (DMSO-*d6*, 500 MHz) δ ppm 9.16 (1H, s, OH), 8.12 (1H, d, J = 8.8 Hz, ArH), 8.09 (1H, dd, J = 7.8, 1.4 Hz, ArH), 7.69–7.75 (2H, m, ArH), 7.44 (1H, t, J = 7.3, 7.4 Hz, ArH), 6.83 (2H, d, J = 8.8 Hz, ArH), 2.99 (6H, s, –$N(CH_3)_2$); ^{13}C-NMR (DMSO-*d6*, 125 MHz) δ ppm 171.98, 154.29, 151.03, 146.85, 137.29, 133.09, 128.99, 124.65, 124.32, 121.45, 118.15, 117.93, 111.40, 39.64. MALDI-TOF MS (negative mode) m/z: 281.820 ($[M]^{-\bullet}$).

NMR spectra

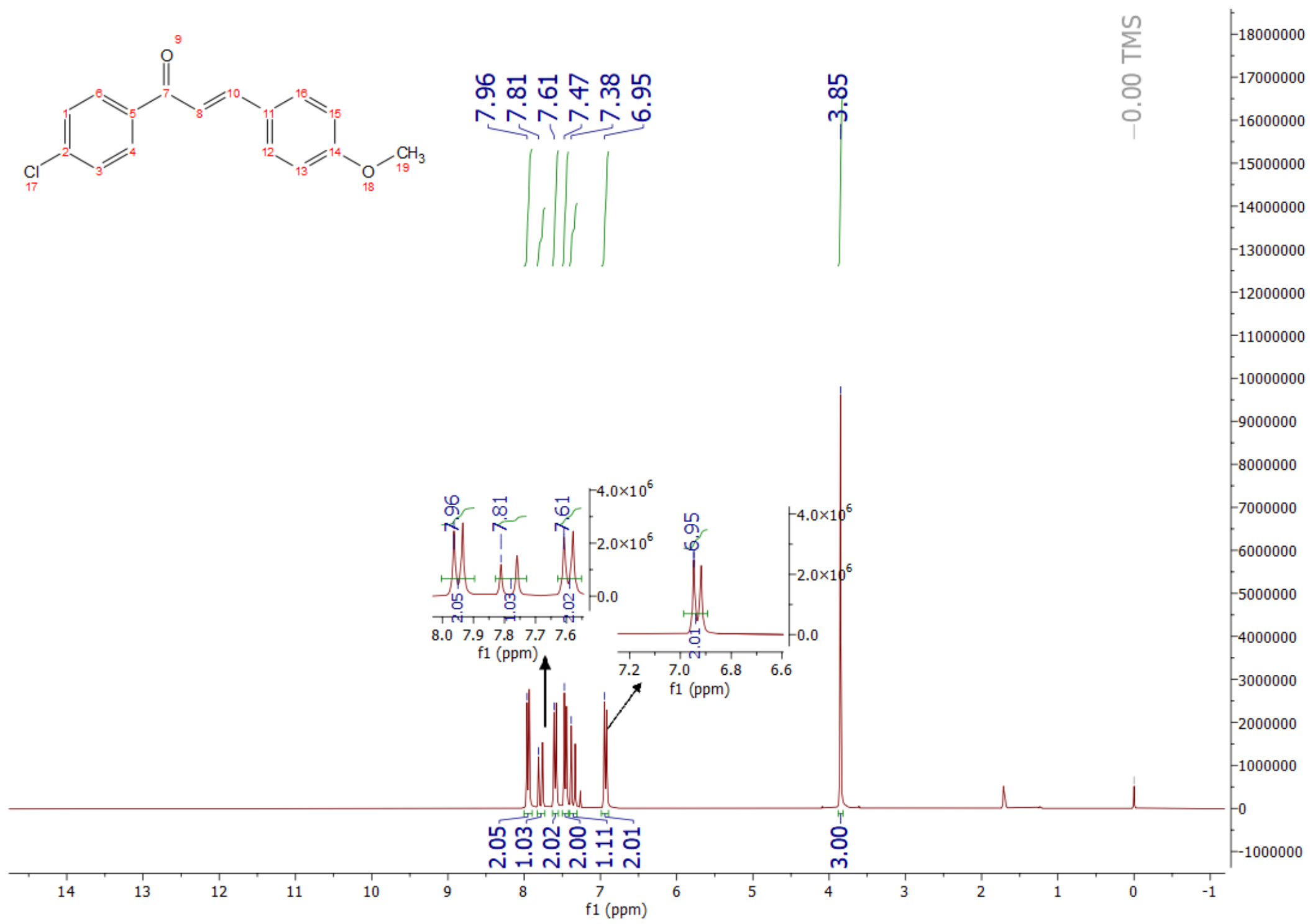

Figure S21: ^{1}H-NMR of compound **1a**

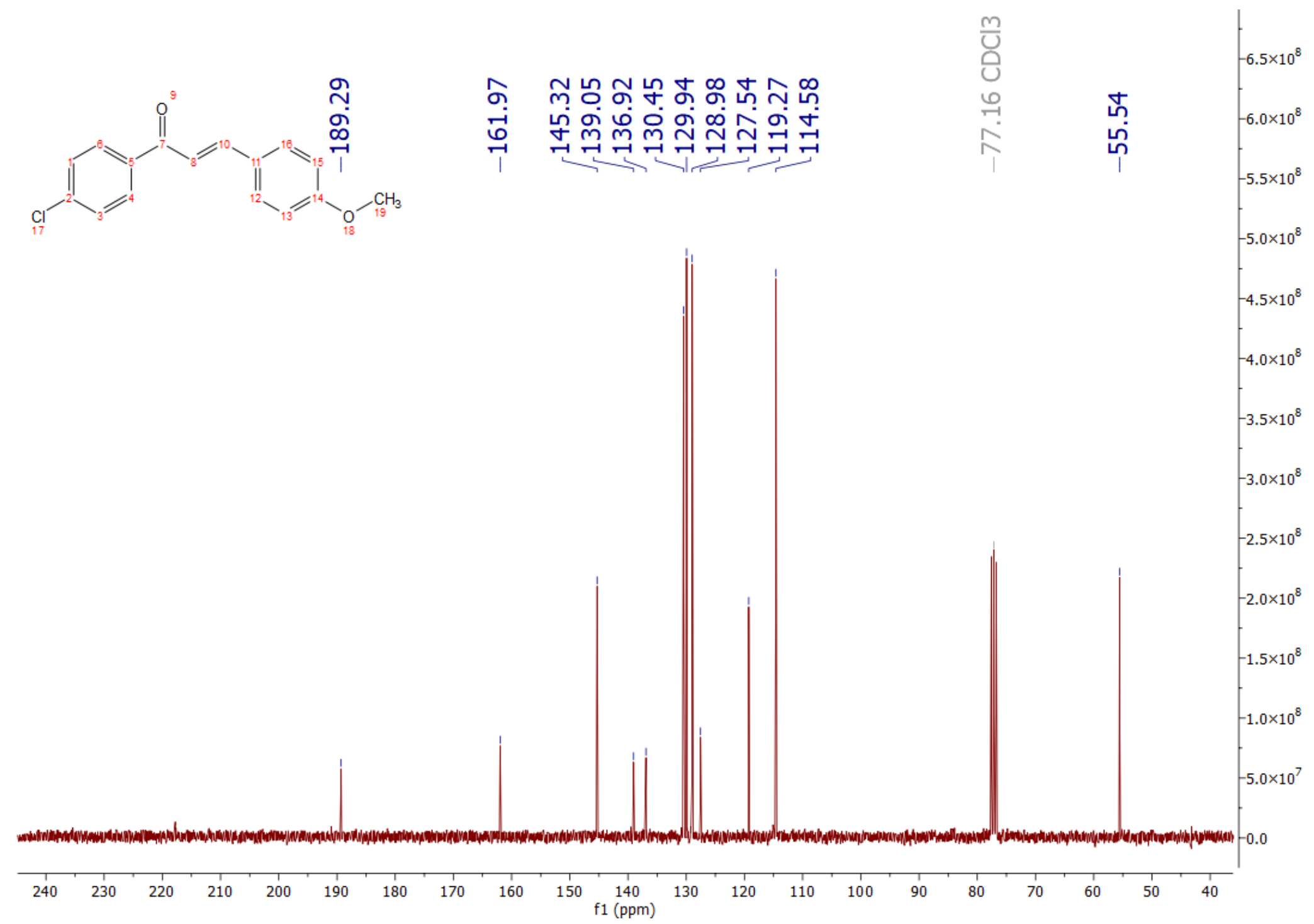

Figure S22: ^{13}C-NMR of compound **1a**

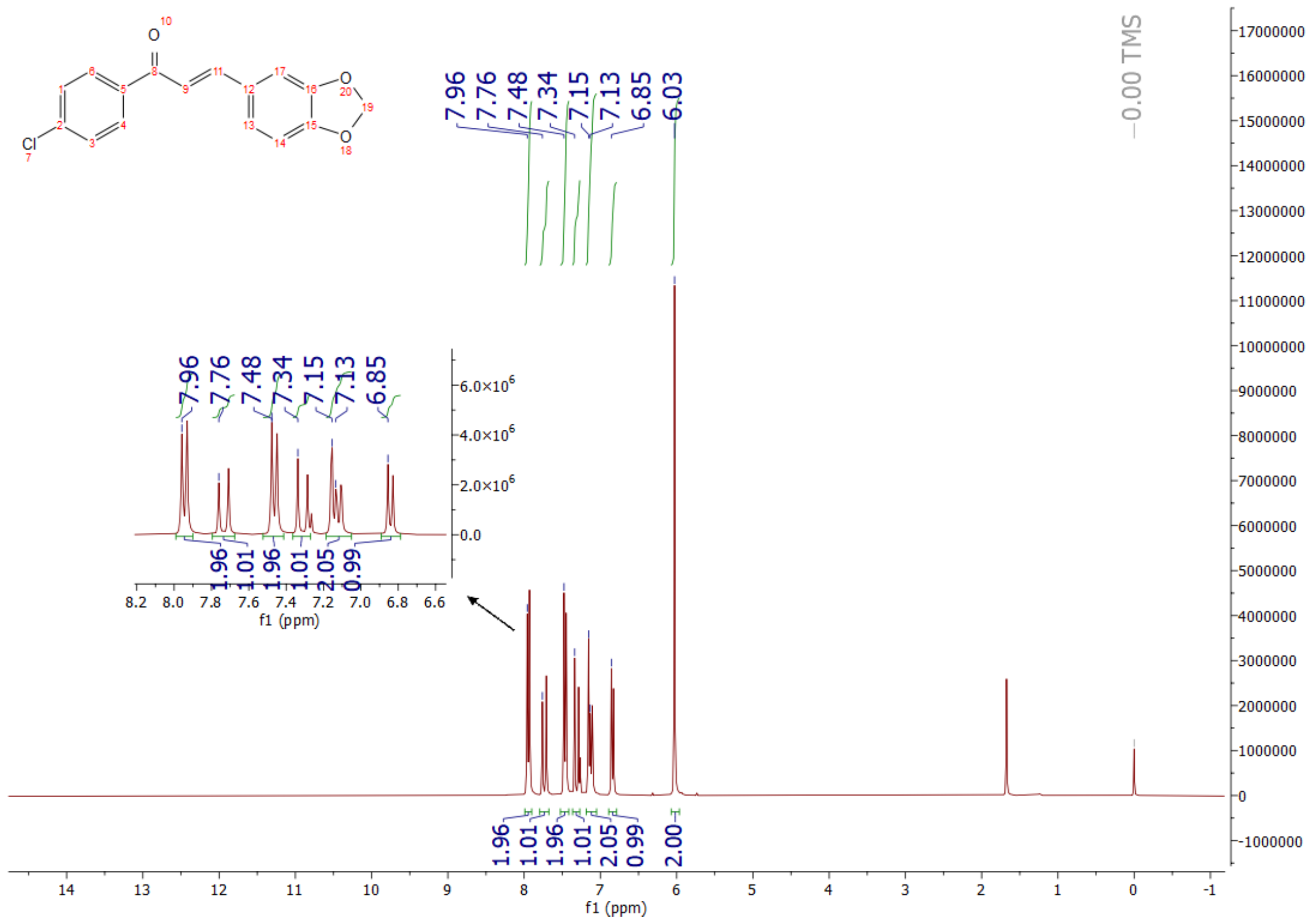

Figure S23: ^{1}H-NMR of compound **1b**

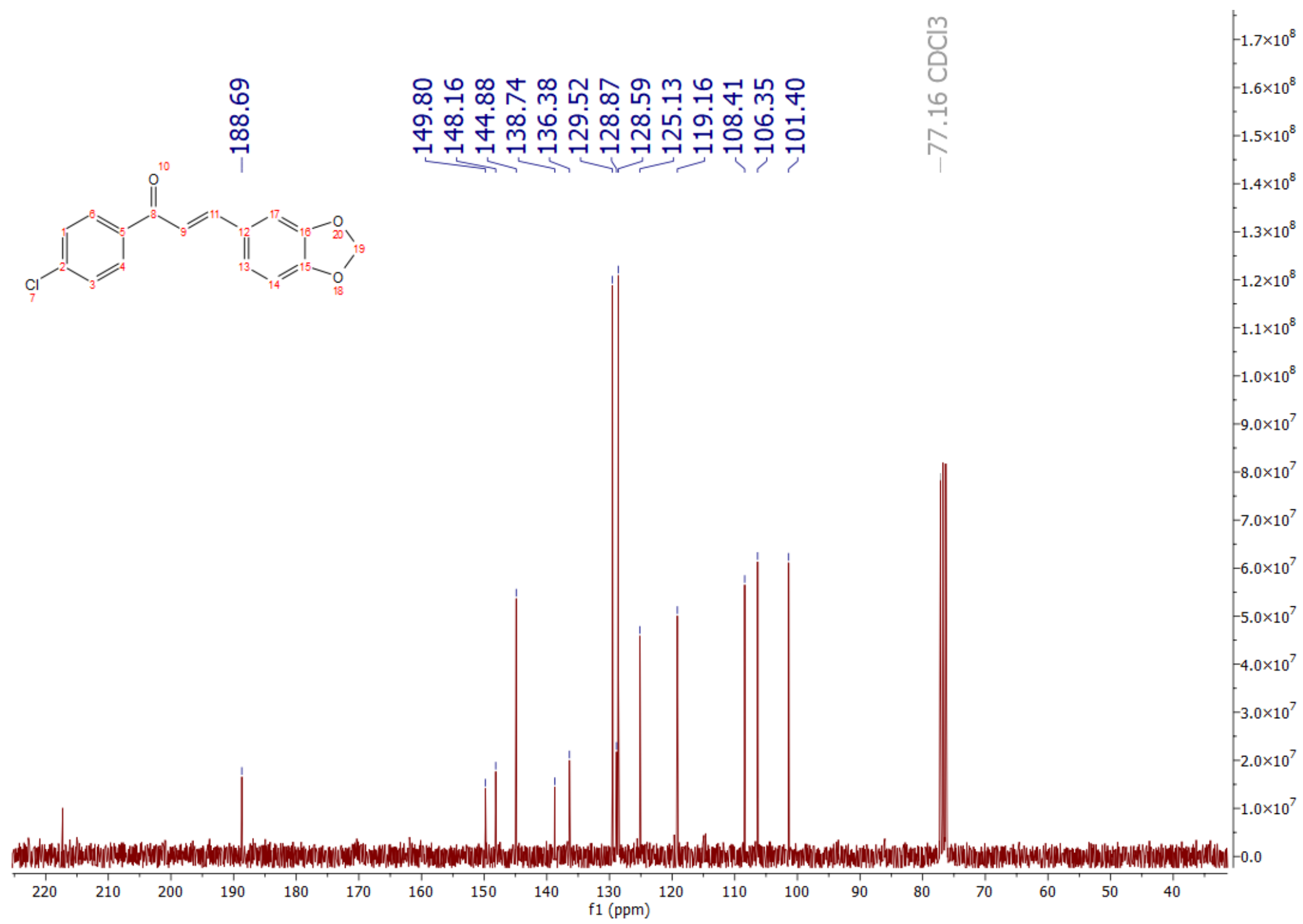

Figure S24: ^{13}C-NMR of compound **1b**

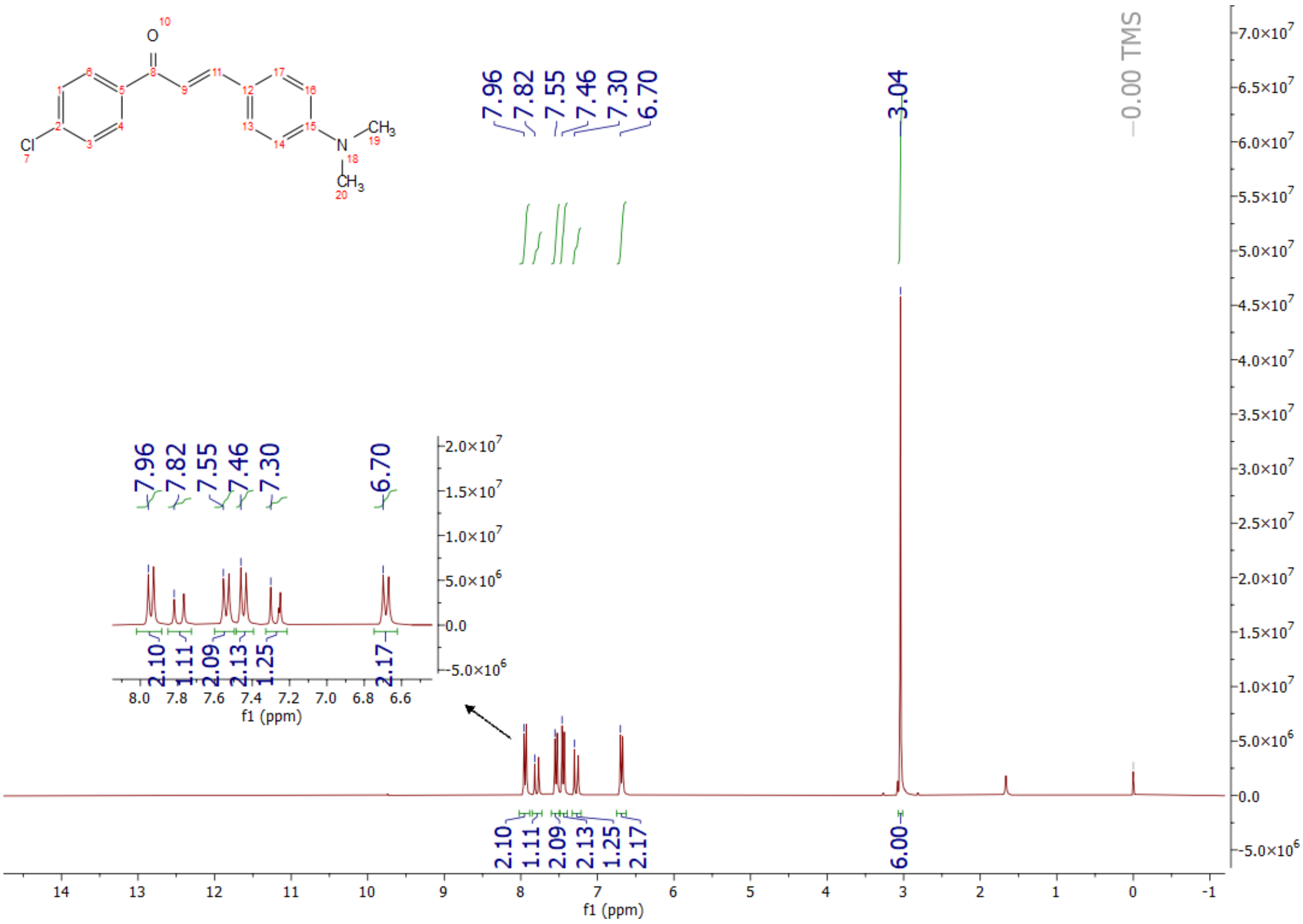

Figure S25: ^{1}H-NMR of compound **1c**

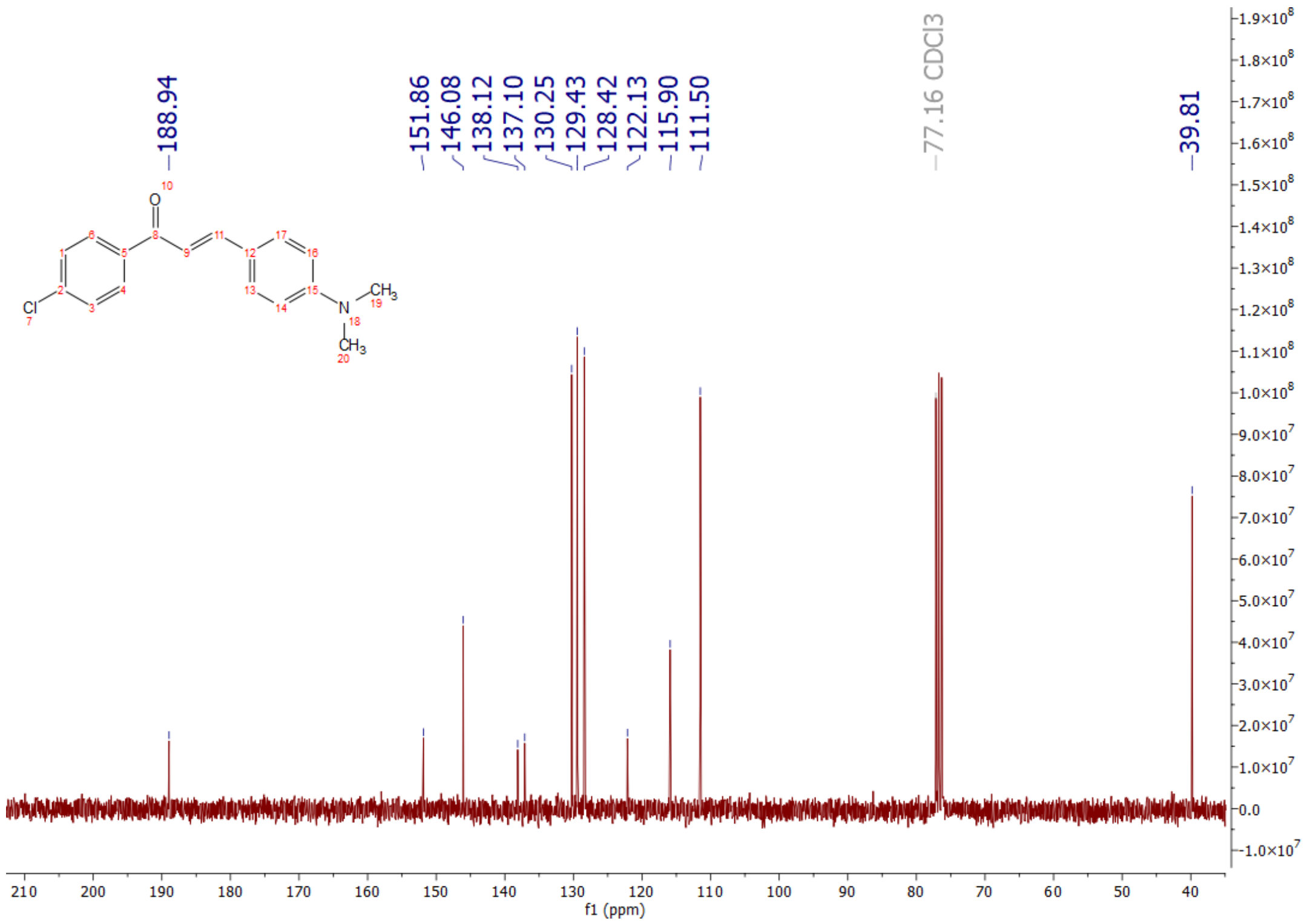

Figure S26: ^{13}C-NMR of compound **1c**

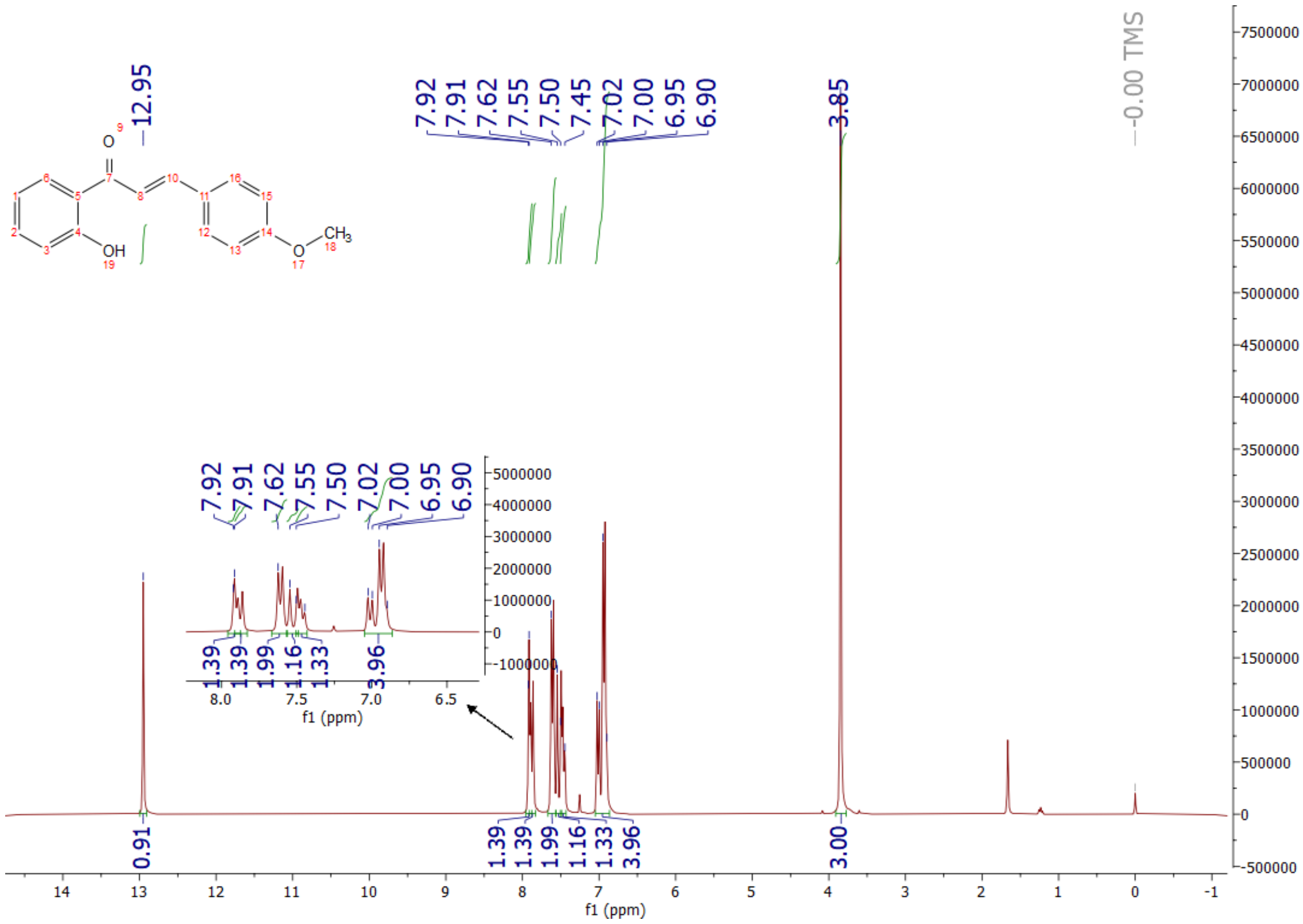

Figure S27: ^{1}H-NMR of compound **2a**

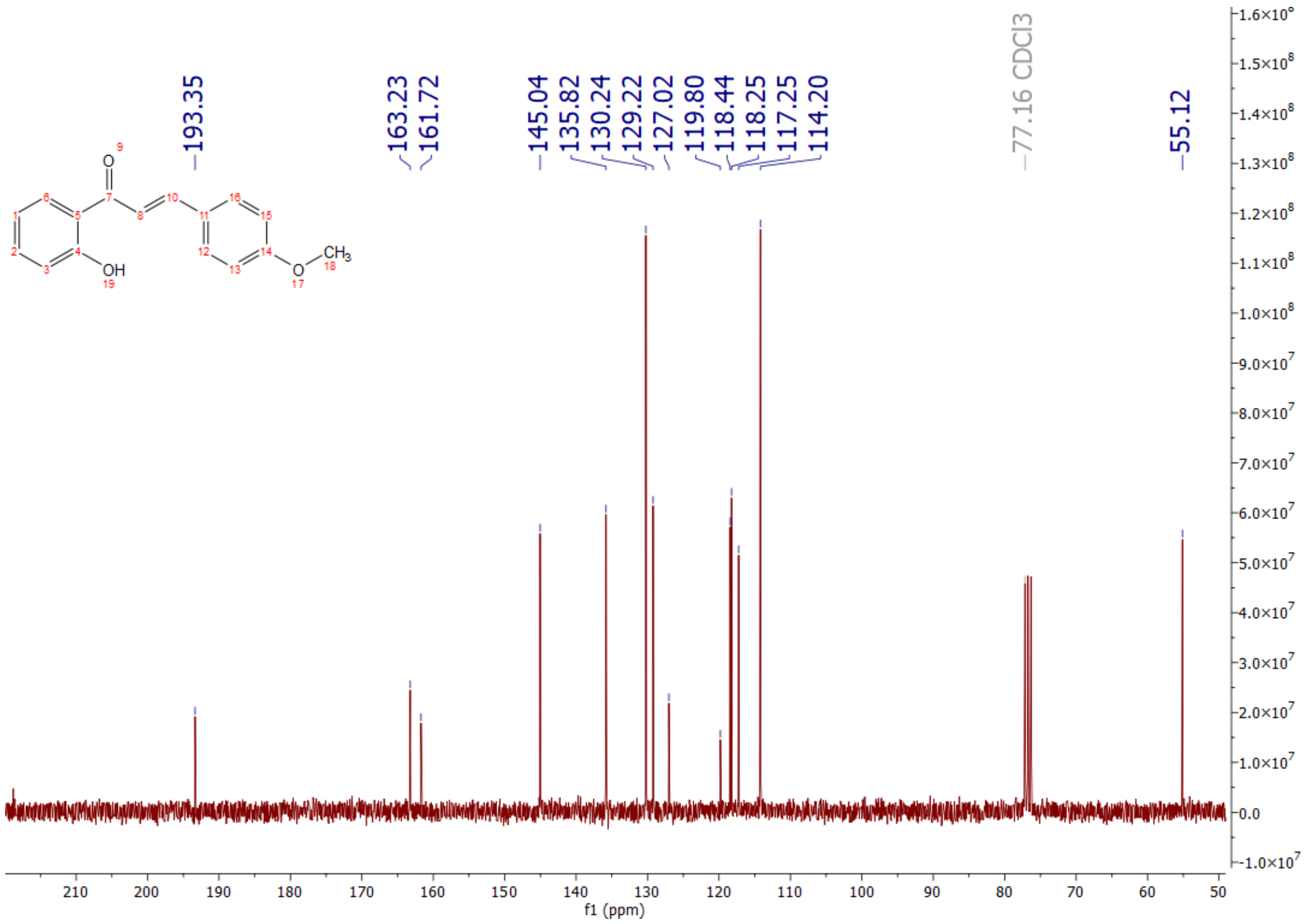

Figure S28: ^{13}C-NMR of compound **2a**

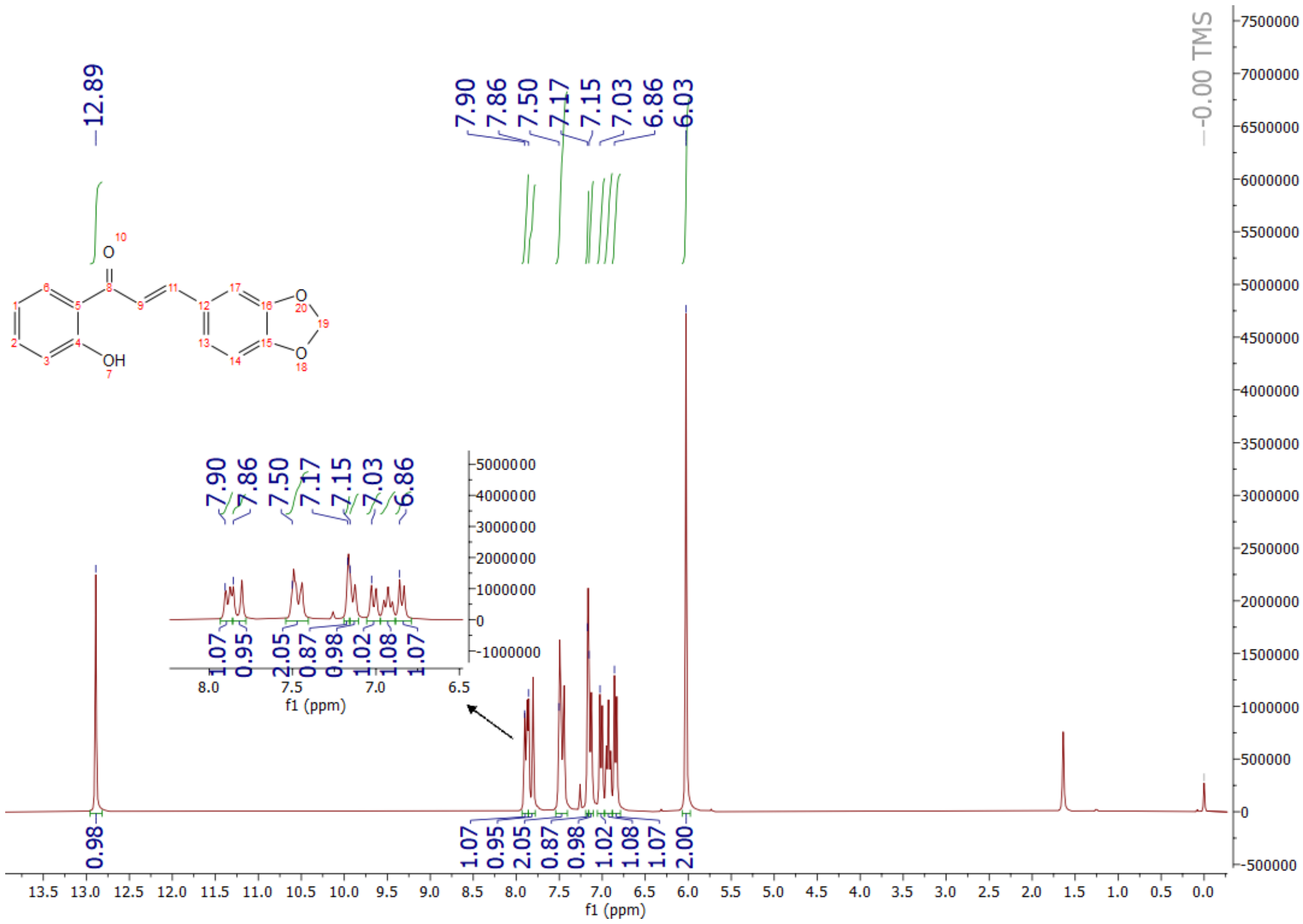

Figure S29: ^{1}H-NMR of compound **2b**

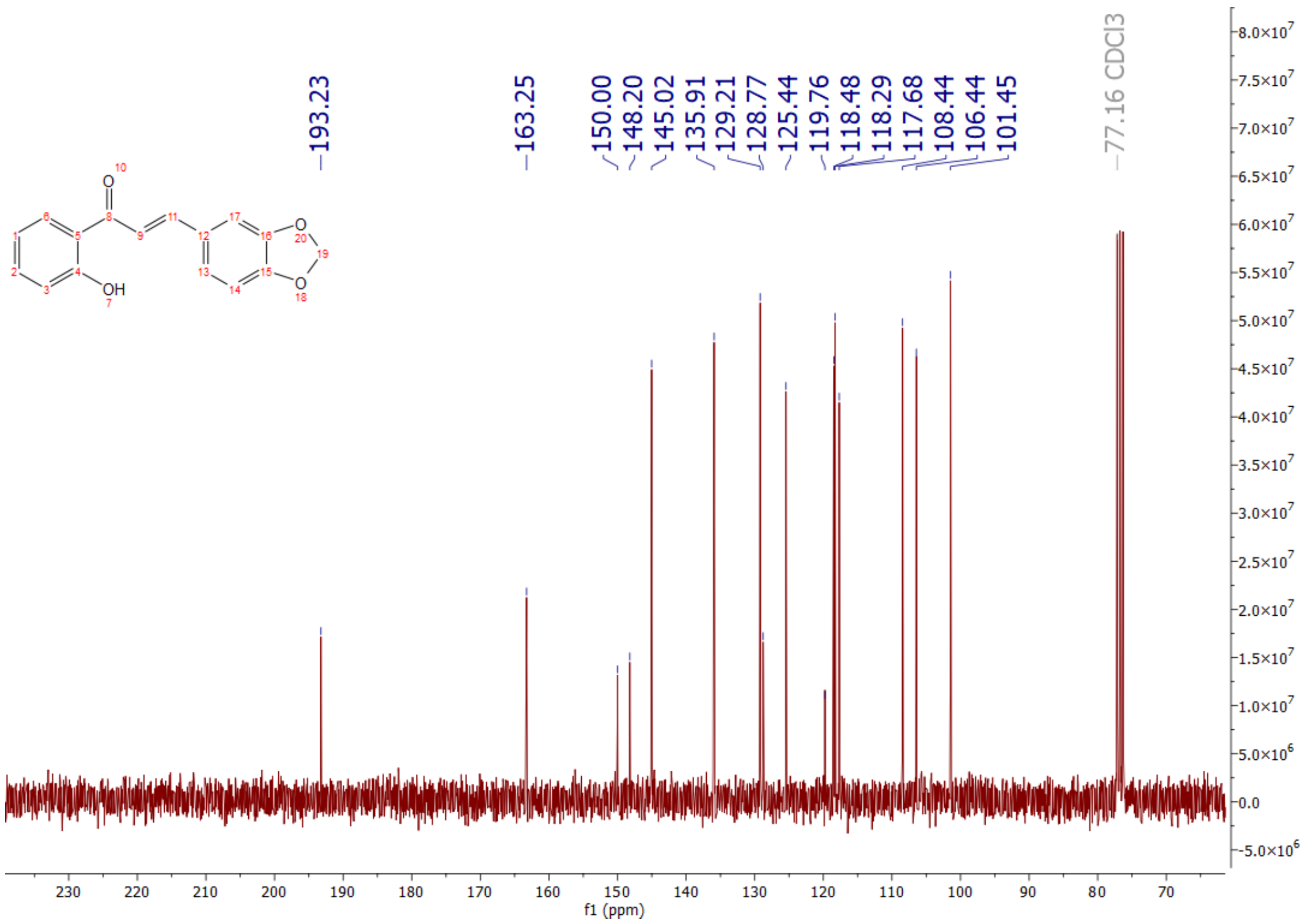

Figure S30: ^{13}C-NMR of compound **2b**

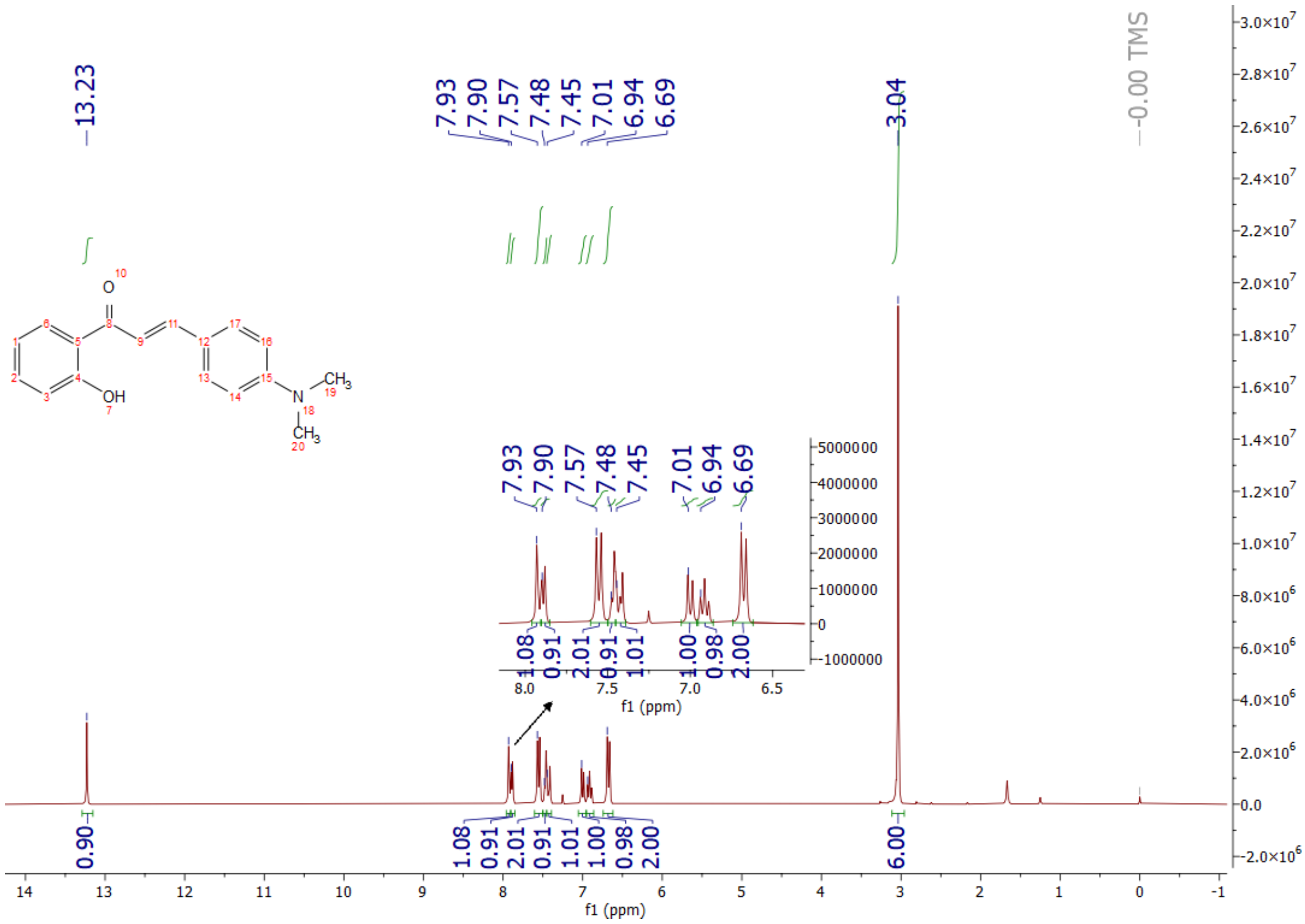

Figure S31: ^{1}H-NMR of compound **2c**

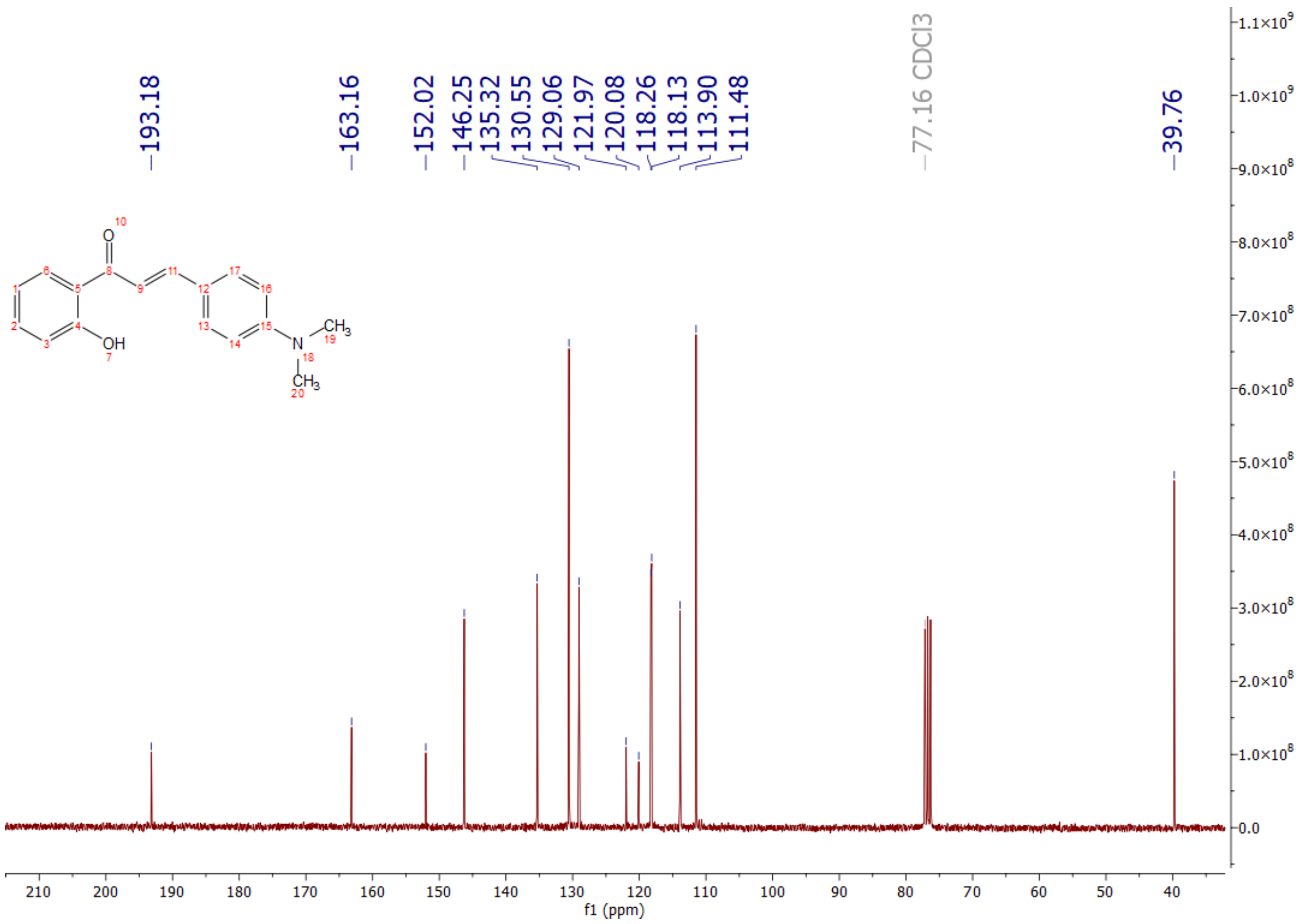

Figure S32: ^{13}C-NMR of compound **2c**

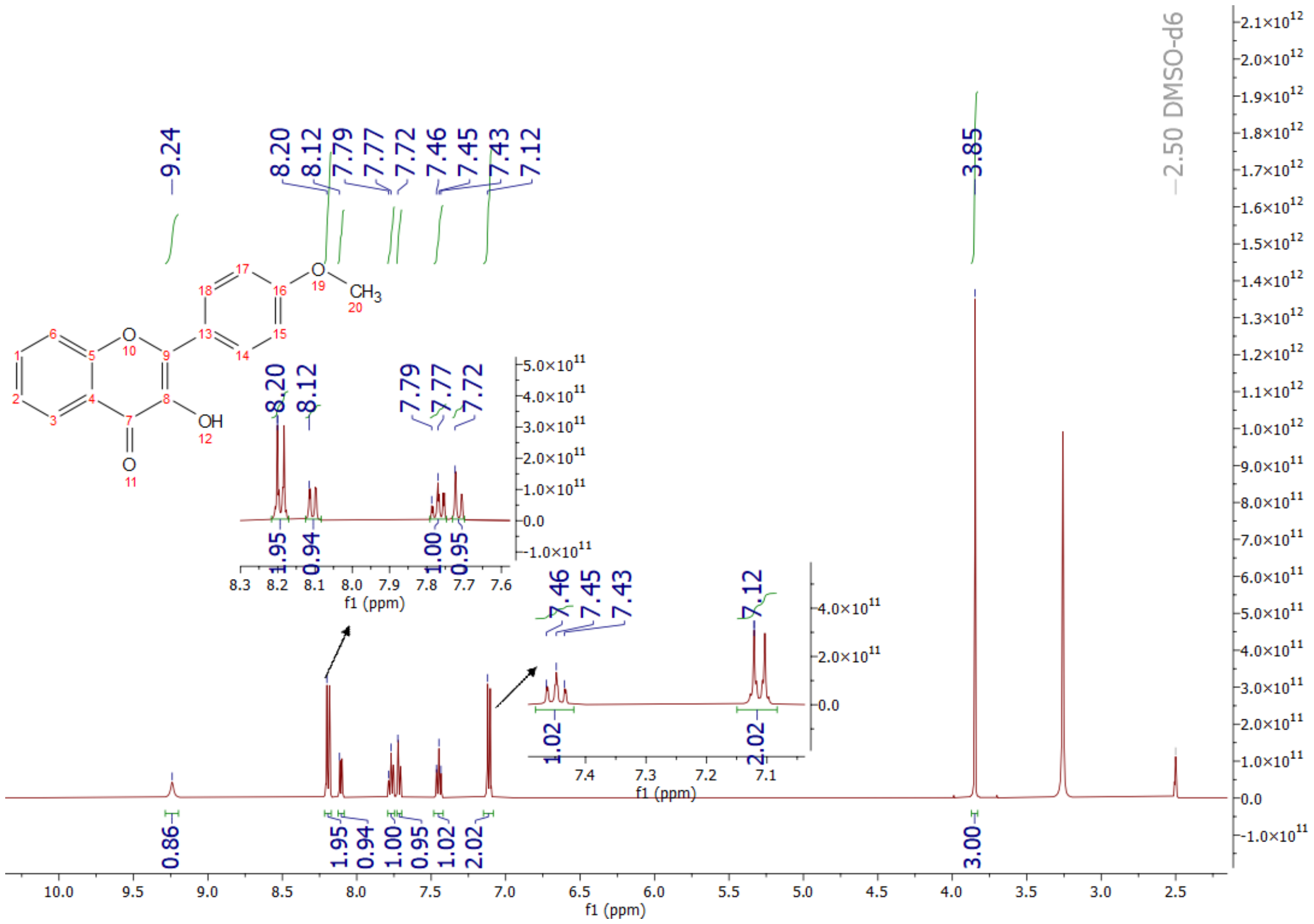

Figure S33: ^{1}H-NMR of compound **f12a**

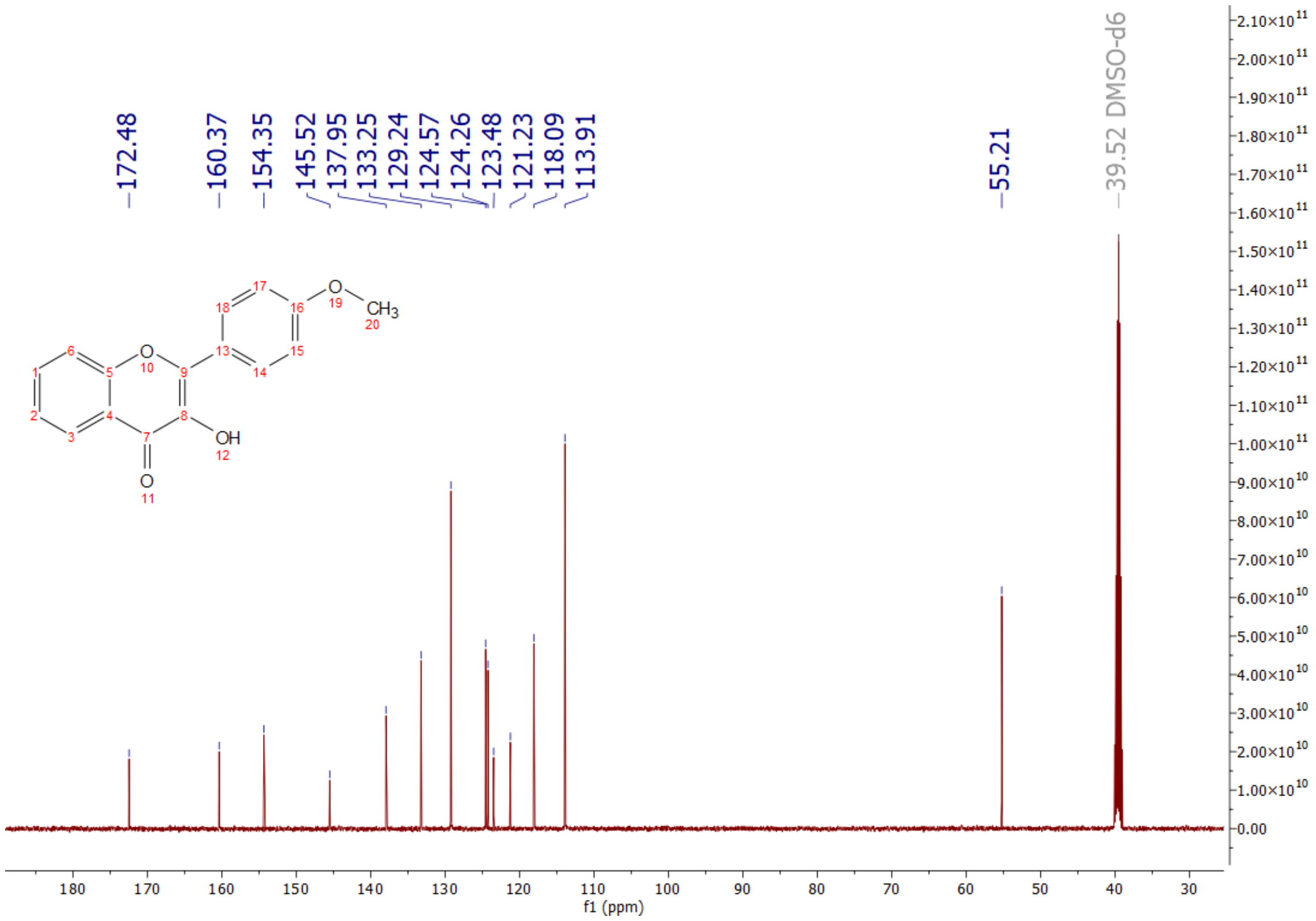

Figure S34: ^{13}C-NMR of compound **f12a**

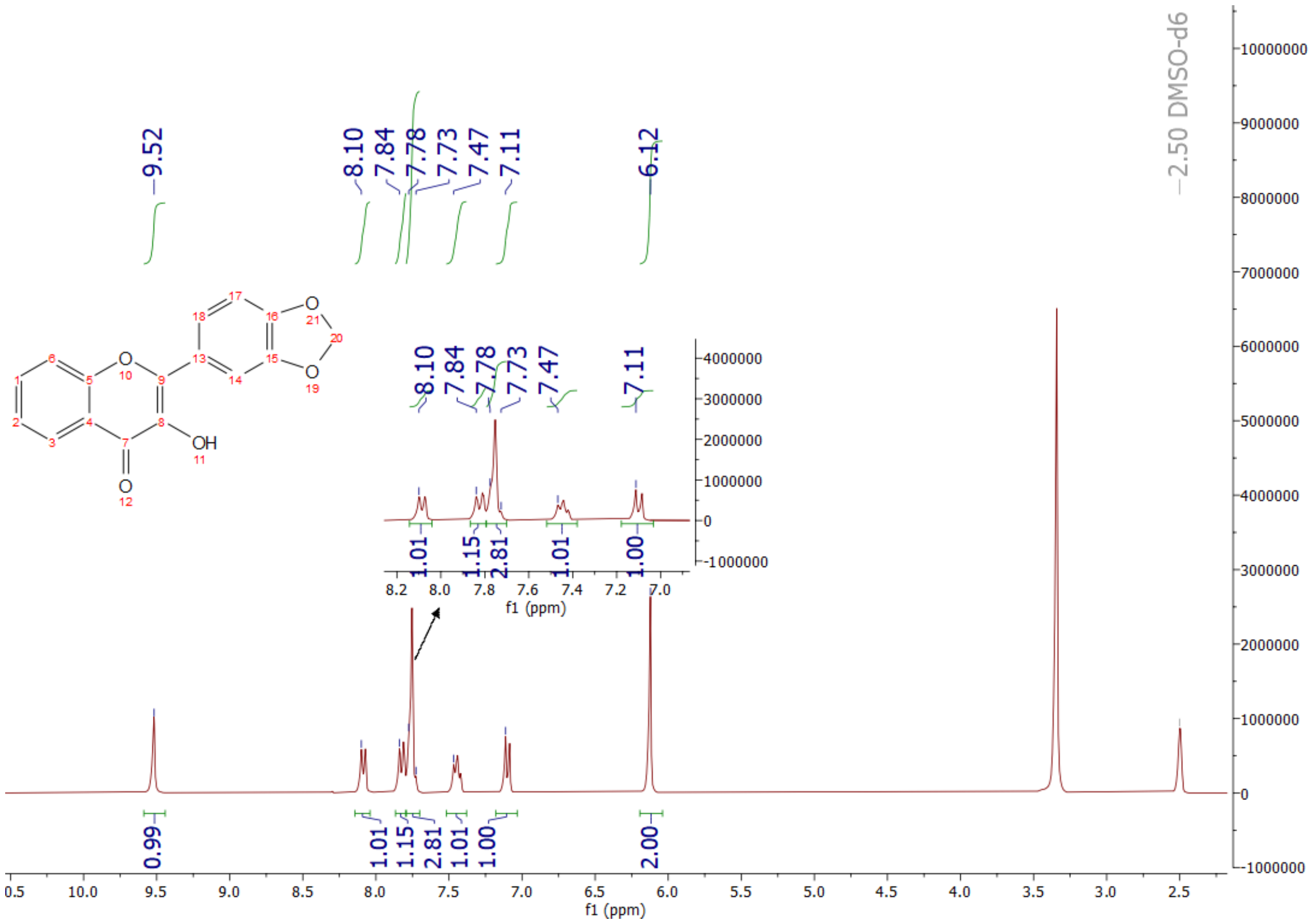

Figure S35: ^{1}H-NMR of compound **f12b**

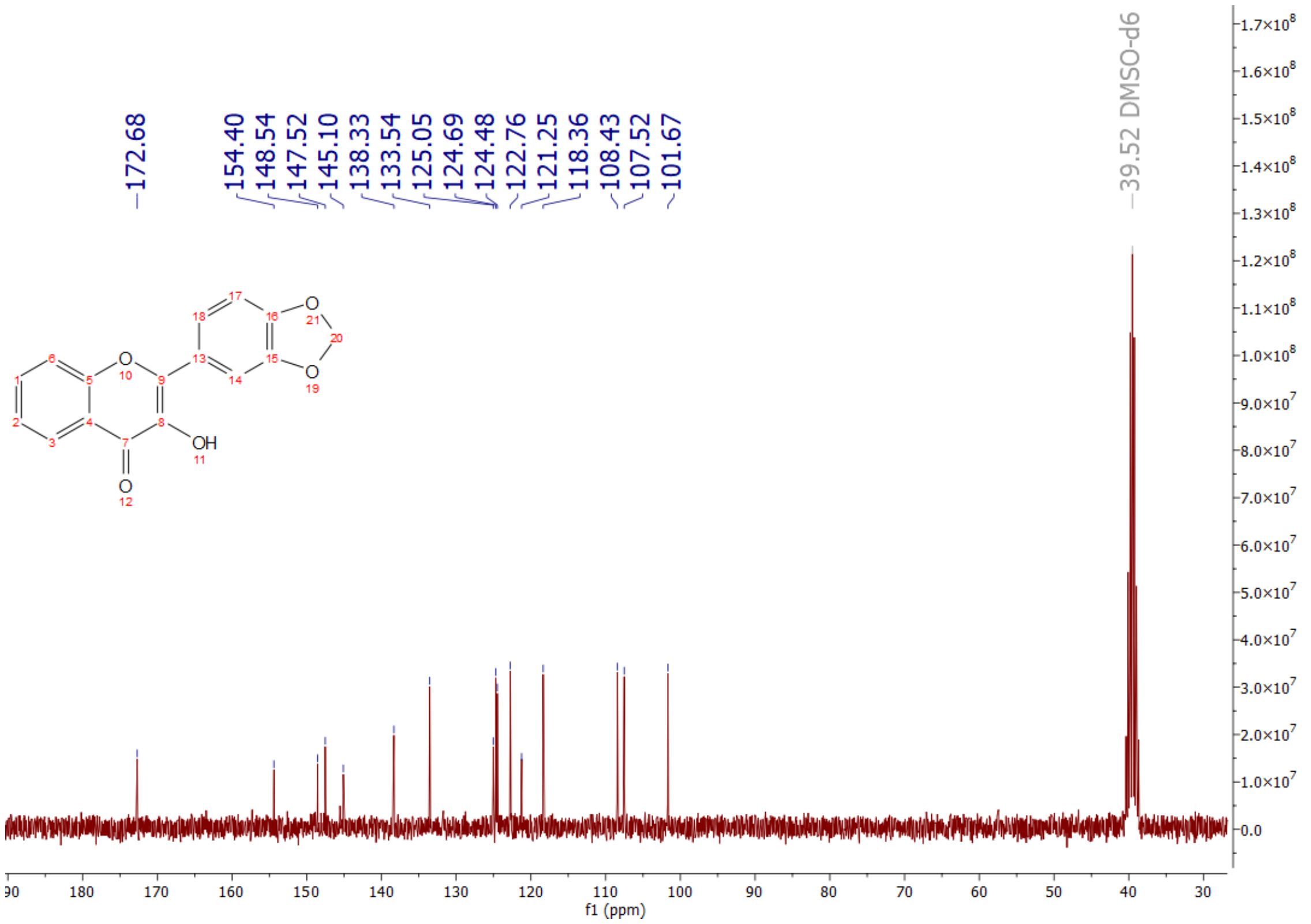

Figure S36: ^{13}C-NMR of compound **f12b**

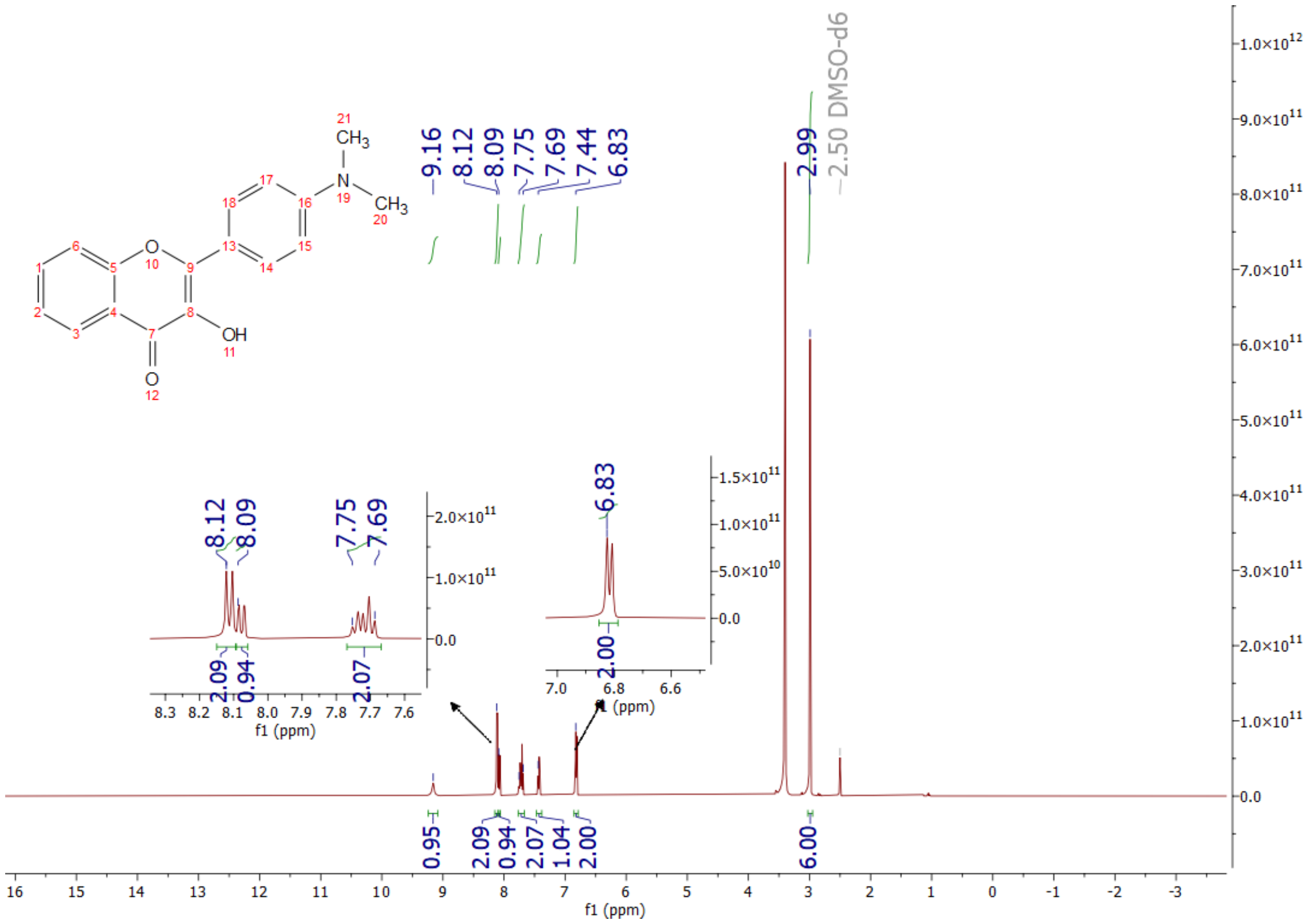

Figure S37: ^{1}H-NMR of compound **f12c**

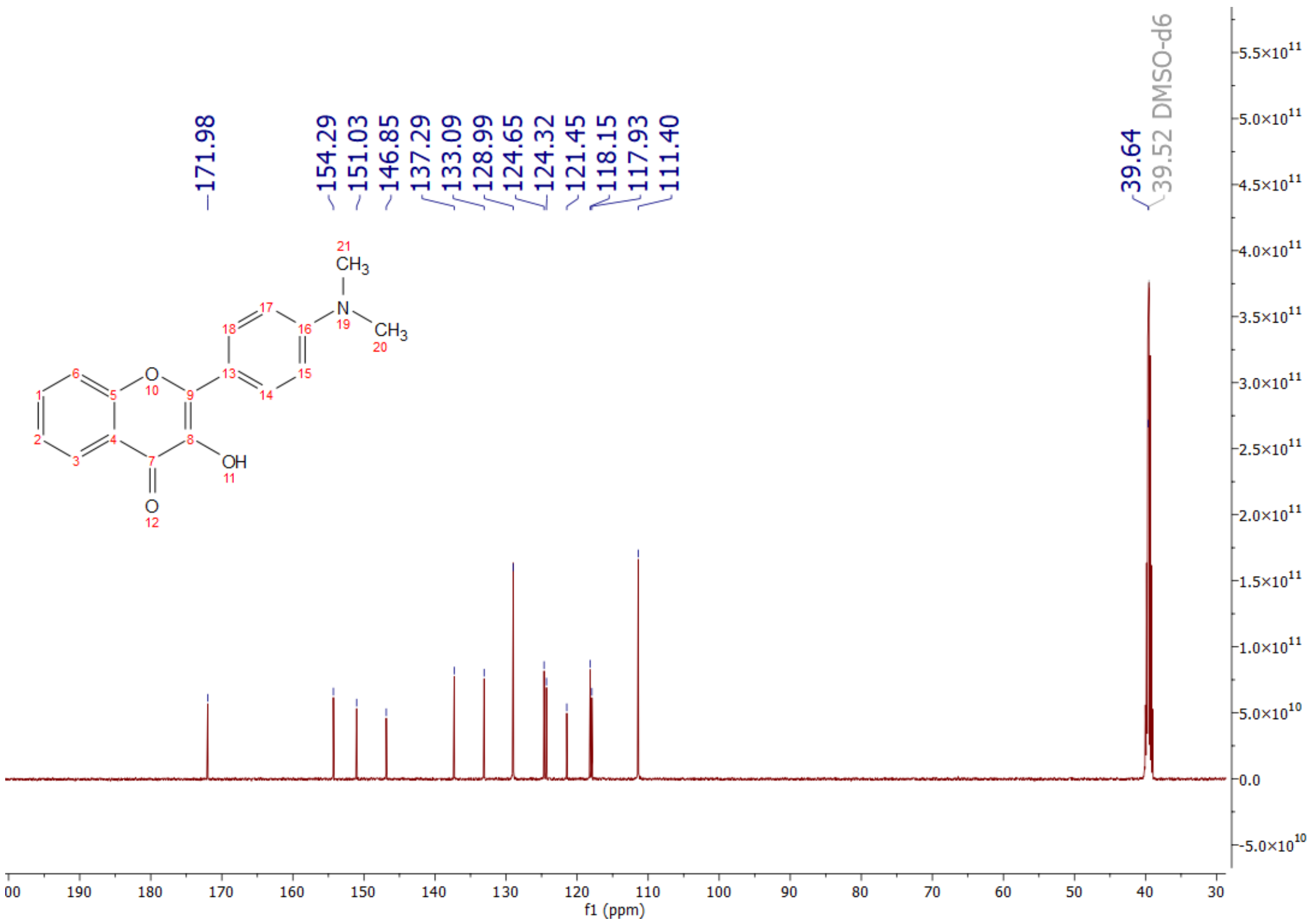

Figure S38: ^{13}C-NMR of compound **f12c**
